# Transformed Recombinant Enrichment Profiling Rapidly Identifies HMW1 as an Intracellular Invasion Locus in *Haemophilus influenzae*

**DOI:** 10.1371/journal.ppat.1005576

**Published:** 2016-04-28

**Authors:** Joshua Chang Mell, Cristina Viadas, Javier Moleres, Sunita Sinha, Ariadna Fernández-Calvet, Eric A. Porsch, Joseph W. St. Geme, Corey Nislow, Rosemary J. Redfield, Junkal Garmendia

**Affiliations:** 1 Department of Microbiology and Immunology, Institute for Molecular Medicine and Infectious Diseases, Center for Genomic Sciences, Drexel University College of Medicine, Philadelphia, Pennsylvania, United States of America; 2 Instituto de Agrobiotecnología, CSIC-Universidad Pública Navarra-Gobierno, Navarra, Spain; 3 Department of Pharmaceutical Sciences and the UBC Sequencing Centre, University of British Columbia, Vancouver, British Columbia, Canada; 4 Department of Pediatrics, Children’s Hospital of Philadelphia, Perelman School of Medicine, University of Pennsylvania, Philadelphia, Pennsylvania, United States of America; 5 Department of Zoology, University of British Columbia, Vancouver, British Columbia, Canada; 6 Centro de Investigación Biomédica en Red de Enfermedades Respiratorias (CIBERES), Madrid, Spain; Fred Hutchinson Cancer Research Center, UNITED STATES

## Abstract

Many bacterial species actively take up and recombine homologous DNA into their genomes, called natural competence, a trait that offers a means to identify the genetic basis of naturally occurring phenotypic variation. Here, we describe “transformed recombinant enrichment profiling” (TREP), in which natural transformation is used to generate complex pools of recombinants, phenotypic selection is used to enrich for specific recombinants, and deep sequencing is used to survey for the genetic variation responsible. We applied TREP to investigate the genetic architecture of intracellular invasion by the human pathogen *Haemophilus influenzae*, a trait implicated in persistence during chronic infection. TREP identified the HMW1 adhesin as a crucial factor. Natural transformation of the *hmw1* operon from a clinical isolate (86-028NP) into a laboratory isolate that lacks it (Rd KW20) resulted in ~1,000-fold increased invasion into airway epithelial cells. When a distinct recipient (Hi375, already possessing *hmw1* and its paralog *hmw2*) was transformed by the same donor, allelic replacement of *hmw2A*_*Hi375*_ by *hmw1A*_*86-028NP*_ resulted in a ~100-fold increased intracellular invasion rate. The specific role of *hmw1A*_*86-028NP*_ was confirmed by mutant and western blot analyses. Bacterial self-aggregation and adherence to airway cells were also increased in recombinants, suggesting that the high invasiveness induced by *hmw1A*_*86-028NP*_ might be a consequence of these phenotypes. However, immunofluorescence results found that intracellular *hmw1A*_*86-028NP*_ bacteria likely invaded as groups, instead of as individual bacterial cells, indicating an emergent invasion-specific consequence of *hmw1A*-mediated self-aggregation.

## Author Summary

Many bacteria are naturally competent, actively taking up DNA from their surroundings and incorporating it into their genomes by homologous recombination. This cellular process has had a large impact on the evolution of these species, for example by enabling pathogens to acquire virulence factors and antibiotic resistances from their relatives. But natural competence can also be exploited by researchers to identify the underlying genetic variation responsible for naturally varying phenotypic traits, similar to how eukaryotic geneticists use meiotic recombination during sexual reproduction to create genetically admixed populations. Here we exploited natural competence, phenotypic selection, and deep sequencing to rapidly identify the *hmw1* locus as a major contributor to intracellular invasion of airway epithelial cells by the human pathogen *Haemophilus influenzae*, a trait that likely allows bacterial cells to evade the immune system and therapeutic interventions during chronic infections. Genetic variation in this locus can strongly modulate bacterial intracellular invasion rates, and possession of a certain allele favors adhesion and self-aggregation, which appear to prompt bacteria to invade airway cells as groups, rather than as individuals. Overall, our findings indicate that targeting HMW1 could block the ability of *H*. *influenzae* to invade airway cells, which would make antibiotic therapy to treat chronic lung infections more effective. Furthermore, our new approach to identifying the genetic basis of natural phenotypic variation is applicable to a wide-range of phenotypically selectable traits within the widely distributed naturally competent bacterial species, including pathogenesis traits in many human pathogens.

## Introduction

Genetic mapping in bacteria historically relied on screening mutant libraries for loss-of-function mutations, followed by laborious isolation and identification of the disrupted loci. Recent innovations in mutagenesis approaches like TnSeq can accelerate the process and aid in characterizing gene function (*e*.*g*. [1,2]), yet such approaches have some limitations. For example: (a) many classes of genetic variation are not evaluated, (b) a suitable loss-of-function screen is typically required, and (c) such techniques ignore naturally occurring within-species phenotypic variation. An alternative is to emulate eukaryotic quantitative genetics approaches, which rely on sexual reproduction to map genetic variation. Rather than isolating the loci responsible for a specific phenotype with disruptive mutations, the QTL (quantitative trait locus) mapping approach identifies the loci and alleles that are directly relevant to phenotypic expression in natural populations.

Bacteria do not reproduce sexually, but genetic transfer mechanisms are widespread, and diverse bacterial species (including many important human pathogens) are naturally competent, able to actively take up and recombine homologous DNA from their surroundings into their chromosomes [3,4]. The value of this genetic transfer mechanism to researchers was seen as early as 1944 by Avery *et al*., when naturally competent *Streptococcus pneumoniae* were used to show that DNA is the genetic material, or “the transforming principle” [5]. But only recently has exploiting natural competence (and other gene transfer mechanisms) become a practical means to investigate the genetic basis for natural phenotypic variation, as massively parallel sequencing technologies have become cost effective [6–9].

The Gram-negative bacterium *Haemophilus influenzae* has a well-characterized natural competence mechanism (reviewed in [4]) and illustrates how experimental natural transformation can be useful for genetic mapping. Under nutrient limitation, cells actively take up double-stranded DNA from their environment through their cell envelope, and this DNA can replace homologous segments of the chromosome by recombination. In the laboratory, a competent *H*. *influenzae* cell will rapidly replace ~0.1–3% of its chromosome with genomic DNA from a divergent *H*. *influenzae* strain [8,10]. These replacements typically involve multiple independent recombination tracts and contain 100s to 1,000s of donor-specific single-nucleotide polymorphisms (SNPs) that span dozens of genes. Insertions and deletions readily transform as parts of longer recombination tracts, albeit with less efficiency than SNPs; this can add or remove whole genes and operons. Thus, a single transformation experiment can give millions of independently transformed recombinants containing all (or nearly all) of the genetic variation that distinguishes the donor and recipient strains [8]. Such pools can be screened or selected for donor phenotypes, and the donor-specific variation in isolated recombinants can be identified by DNA sequencing. A previous small-scale screen of <100 transformed recombinant clones identified a bacterial QTL with a ~10-fold effect on transformability itself, though this used laborious quantitative assays of individual clones that had already been sequenced [8].

Though typically a commensal of the nasopharynx, *H*. *influenzae*—especially in nonencapsulated or nontypeable forms (NTHi)—can cause middle ear infections (otitis media), community-acquired pneumonia, exacerbations of chronic obstructive pulmonary disease (COPD), conjunctivitis, and sometimes more severe invasive diseases [11]. Infections often persist and recur despite host production of bactericidal antibodies and the use of antibiotic therapy. Our understanding of the molecular mechanisms involved in the progression and persistence of *H*. *influenzae* infections remains limited, but identical strains have been repeatedly isolated from the lungs of COPD patients in serial clinic visits, suggesting that *H*. *influenzae* has traits that promote chronic infection [12,13].

Current evidence indicates that *H*. *influenzae* is a facultative intracellular pathogen, and host cell invasion may allow bacterial cells to temporarily evade the immune system and therapeutic interventions [14,15]. *H*. *influenzae* invades a variety of cell types [16–20], and viable NTHi have been found within host cells of adenoid tissues and bronchial biopsies [21,22]. After intracellular invasion, *H*. *influenzae* cells remains non-proliferative and resides within membrane-bound vacuoles with features of late endosomes [23,24] or freely within the cytoplasm [25], and intracellular bacteria eventually die after persisting for variable lengths of time [26]. While several host factors have been identified as important for intracellular invasion, much less is known about the bacterial factors responsible for this process.

**Fig 1:**
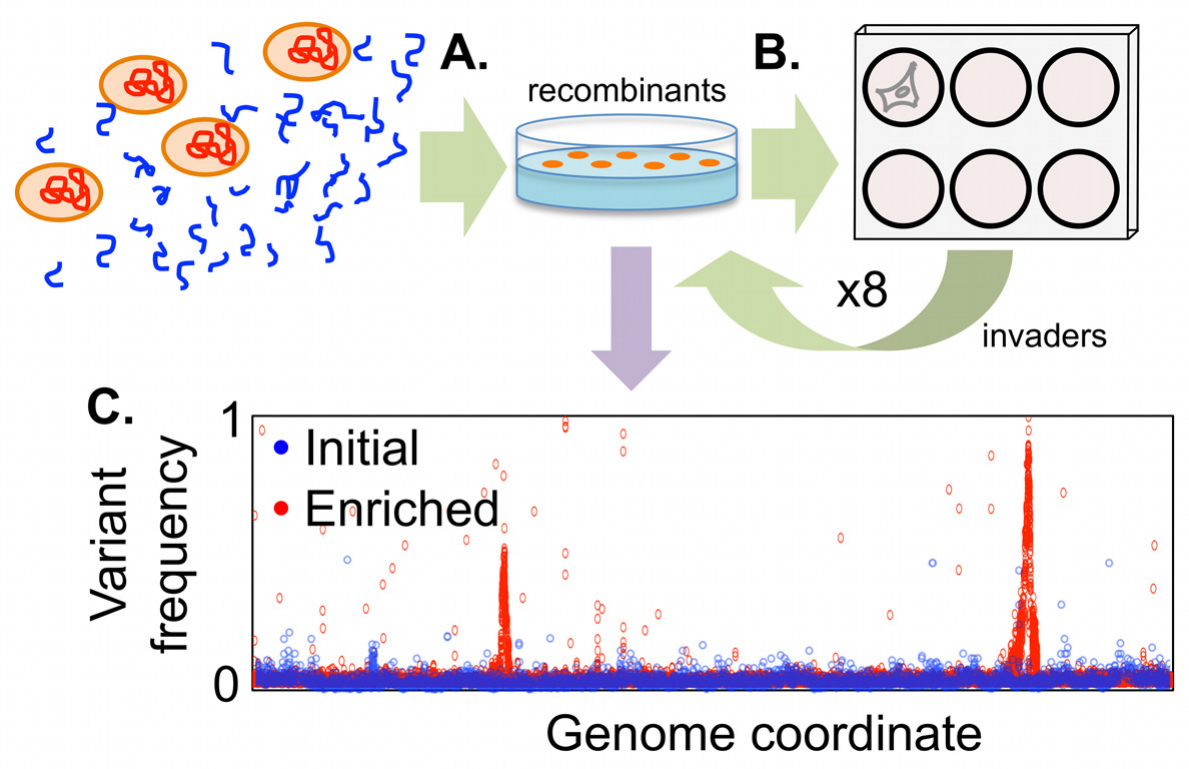
Schematic of transformed recombinant enrichment profiling (TREP) to map *H*. *influenzae* intracellular invasion genes. **(A)** Genomic DNA from a strain with high invasiveness is transformed into naturally competent cells of a strain with poor invasiveness. This produces complex pools of recombinants, in which short stretches of donor DNA replace homologous recipient DNA in individual recombinants. **(B)** Rare recombinants with increased invasiveness are enriched by serial passage of recombinant pools through A549 alveolar epithelial cells using gentamicin protection assays. Finally **(C)**, the selected locus/loci are identified from donor allele frequencies measured by deep sequencing.

We chose the intracellular invasion phenotype as a model to test the genetic mapping strategy described above, which we have named “transformed recombinant enrichment profiling”, or TREP (summarized in Fig 1). We chose this phenotype because entry of *H*. *influenzae* into airways cells: (a) is easily selectable in lab culture, (b) displays wide phenotypic variation between clinical isolates, and (c) is likely to be an important factor in chronic infections. Application of TREP rapidly identified the *hmw1* adhesin as a factor crucial for intracellular invasion.

## Results

### Comparison of three diverse *H*. *influenzae* isolates

Three criteria were used to choose the donor and recipient strains: (a) substantially higher invasiveness of the donor over the recipients, (b) high natural transformability of the recipients, and (c) available genome references with many genetic markers distinguishing the donor from the recipients (S1 Text and S1 Fig). Three strains were assayed: the standard laboratory strain (Rd KW20, hereafter referred to as Rd) and two clinical isolates from pediatric patients with otitis media (Hi375 and 86-028NP). All three have complete genome sequences available [27–29]. Rd and Hi375 are highly transformable, while 86-028NP is not [30,31]. Rd is known to be a poor invader of several epithelial cell lines [32]; Hi375 has previously been used in studies of intracellular invasion [15,24]; and 86-028NP is known to be highly virulent in a chinchilla model of otitis media [33–36]. Antibiotic resistant derivatives of all three strains were produced to allow subsequent tracking of transformation events and genetic background, yielding strains Rd Spc^R^, Hi375 Str^R^, and 86-028NP Nov^R^ Nal^R^, hereafter referred to as RdS, HiT, and NpNN, respectively (S1 Table).

**Fig 2:**
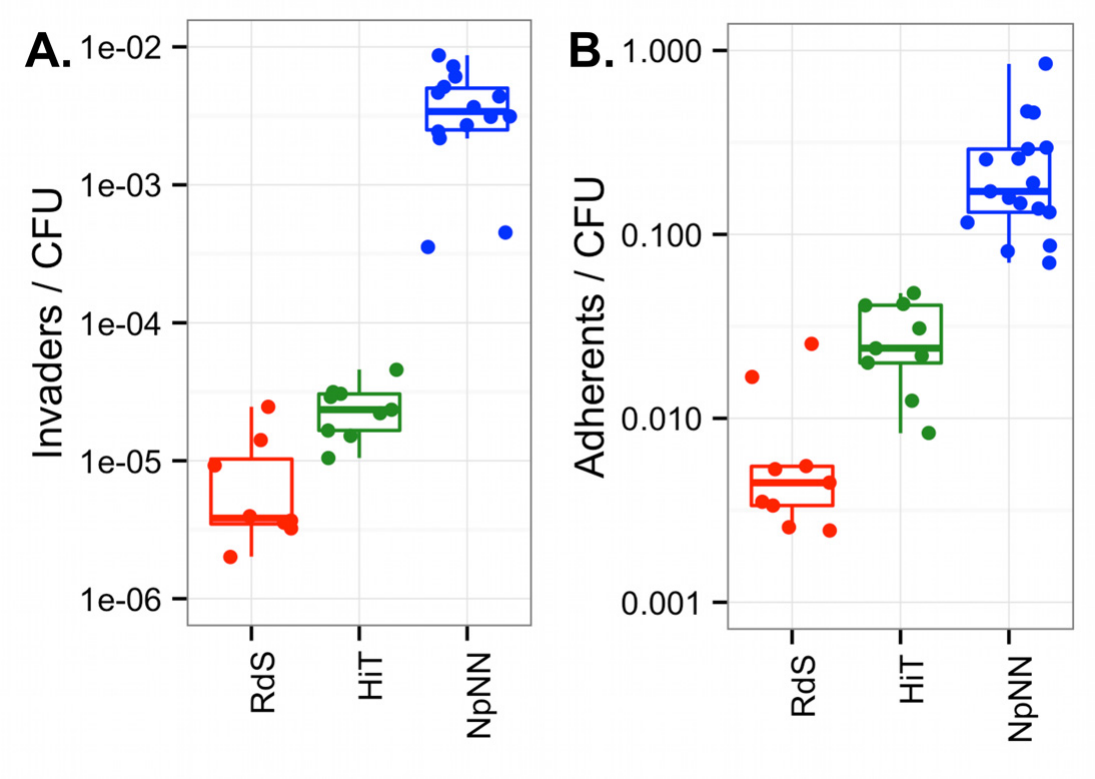
Invasion and adhesion in the parent strains. **(A)** “Invaders/CFU” was calculated as the total gentamicin-protected CFU / total input CFU. **(B)** “Adherents/CFU” was calculated as above with gentamicin treatment excluded. Triplicate experiments were conducted three times for RdS and HiT recipients and five times for the NpNN donor. Boxplots outline the first and third quartiles, with the thick horizontal line indicating the median, and the whiskers extend 1.5 times the interquartile distance.

Intracellular invasion frequencies were evaluated by gentamicin protection assays with A549 airway epithelial cells, and quantified as gentamicin-protected bacterial colony forming units (CFU) relative to the original inoculated CFU (hereafter “Invaders/CFU”). Gentamicin protection assays found that NpNN is a highly efficient invader of A549 cells with ~10^−2^ invaders/CFU, whereas both HiT and RdS invade at ~100-fold and ~1,000-fold lower frequencies, respectively (Fig 2A; one-way ANOVA p<<0.01, and p<<0.01 for all three pairwise comparisons by *post hoc* testing using Tukey’s HSD).

Several controls were performed, both for assays of the three parental strains and during the serial passage experiments described below: (a) To ensure that spontaneous gentamicin resistance was not responsible for survival in gentamicin protection assays, equivalent bacterial suspensions were treated in the absence of A549 cells, yielding no viable CFUs (limit of detection <10^−8^), and gentamicin-protected CFUs recovered as colonies on plates remained gentamicin sensitive. (b) To ensure gentamicin treatment was complete, culture supernatants of infected A549 cells treated with gentamicin were plated, rendering no viable CFUs (limit of detection <10^−8^). (c) To ensure that introduction of selectable markers had no effect on intracellular invasion, these strains were compared to their progenitors, finding that they had comparable phenotypes (S2A Fig, one-way ANOVA p-values > 0.1 within each strain background).

Adhesiveness of the three parent strains to A549 cells was assayed similarly to invasiveness, except that the gentamicin treatment was omitted; “Adherents/CFU” was calculated as the CFU that remained associated with A549 cells after incubation and washing, relative to CFU of the input inoculum. The NpNN strain was the most adherent, with ~10% of the infecting cells remaining associated with A549 cells (Fig 2B). The HiT strain was intermediate (~10-fold lower than NpNN), whereas RdS had ~100-fold lower adherence (p<<0.01 by ANOVA and all for three pairwise comparisons). As with invasion, antibiotic resistant derivatives had adhesiveness comparable to that of the progenitors (S2B Fig).

### Gentamicin protection strongly selects for intracellular invaders

A calibration experiment was used to test the strength of the experimental selection applied by the gentamicin protection assay. Mixtures of a high invasion strain (86-028NP Nov^R^) and a low invasion strain (Rd Str^R^) were passaged twice: Bacterial cells were used to infect A549 cells and invaders were recovered after gentamicin treatment. The recovered colonies were then pooled and used for a second infection. This found that the highly invasive strain easily out-competed the poorly invasive one, even when starting at a 1 to 10,000 disadvantage, dominating the population after the second infection (S3 Fig). The high enrichment was not due to a growth rate advantage of 86-028NP, which has a slower doubling time than both recipients [30]. These data demonstrate that even rare recombinants could be highly enriched from complex pools using serial selection.

### Summary of transformed recombinant enrichment profiling (TREP)

The experimental design for isolating bacterial intracellular invasion genes by TREP is depicted in Fig 1, summarized below, and described in detail in subsequent sections. (a) Donor genomic DNA from NpNN was used to transform naturally competent cells of two low invasion strains, RdS or HiT. (b) Pools of ~10^5^ recombinant clones were enriched for those that conferred increased invasiveness by serial passages through A549 cells by gentamicin protection. Material from each cycle was stored to use for replicate quantitative assays and DNA extractions. (c) Genomic DNA from pools was sequenced to high coverage, and donor-specific allele frequencies were calculated at diagnostic SNPs. Sequencing and alignment statistics are summarized in S2, S3 and S4 Tables, and further details are presented in S1 Text and Materials and Methods.

By profiling genome-wide donor allele frequencies from independent experiments, a gene responsible for high invasion by NpNN was rapidly identified in both recipients, RdS or HiT. Individual clones were isolated after the fourth cycle of selection for genome sequencing and phenotypic analysis of recombinants and mutant derivatives. The results reveal a novel role for the adhesin-encoding *hmw1A* gene in intracellular invasion, beyond its previously described role in adhesion [37,38].

### Generation of complex recombinant pools of natural transformants

Recombinant pools were made by incubating high molecular weight genomic DNA from NpNN with naturally competent cultures of either RdS or HiT (Fig 1A) [39]. Transformation by the two antibiotic resistance markers (Nov^R^ and Nal^R^) indicated that ~15% of cells in each culture were competent and predicted that a given donor-specific SNP would be found in ~1% of Nov^R^ or Nal^R^ colonies, consistent with previously measured values (S5 Table) [8]. Selection for donor-specific antibiotic resistance alleles ensured elimination of untransformed recipient cells, and selection for recipient-specific resistances limited cross-contamination observed in preliminary experiments (Materials and Methods). This procedure generated four separate sets of recombinant clones (for the RdS recipient: Spc^R^ Nov^R^ and Spc^R^ Nal^R^; for the HiT recipient: Str^R^ Nov^R^ and Str^R^ Nal^R^). For each set, ~10^5^ colonies were harvested into pools, thoroughly mixed, and stored prior to infections. We thus predicted that each pool would have millions of cells for which (nearly) any given donor-specific variant would be present. Sequencing of the initial recombinant pools provided several lines of evidence that the TREP approach would be practical (S1 Text): First, a single round of selection for the donor-specific antibiotic resistance alleles was sufficient to map the resistances to single nucleotide resolution (S4A and S4B Fig and S6 Table). Second, recombinant pools contained low frequency donor-specific alleles across the genome, both SNPs and and structural variants (S4C Fig and S7 Table).

### Serial selection by gentamicin protection enriches for invasive recombinants

**Fig 3:**
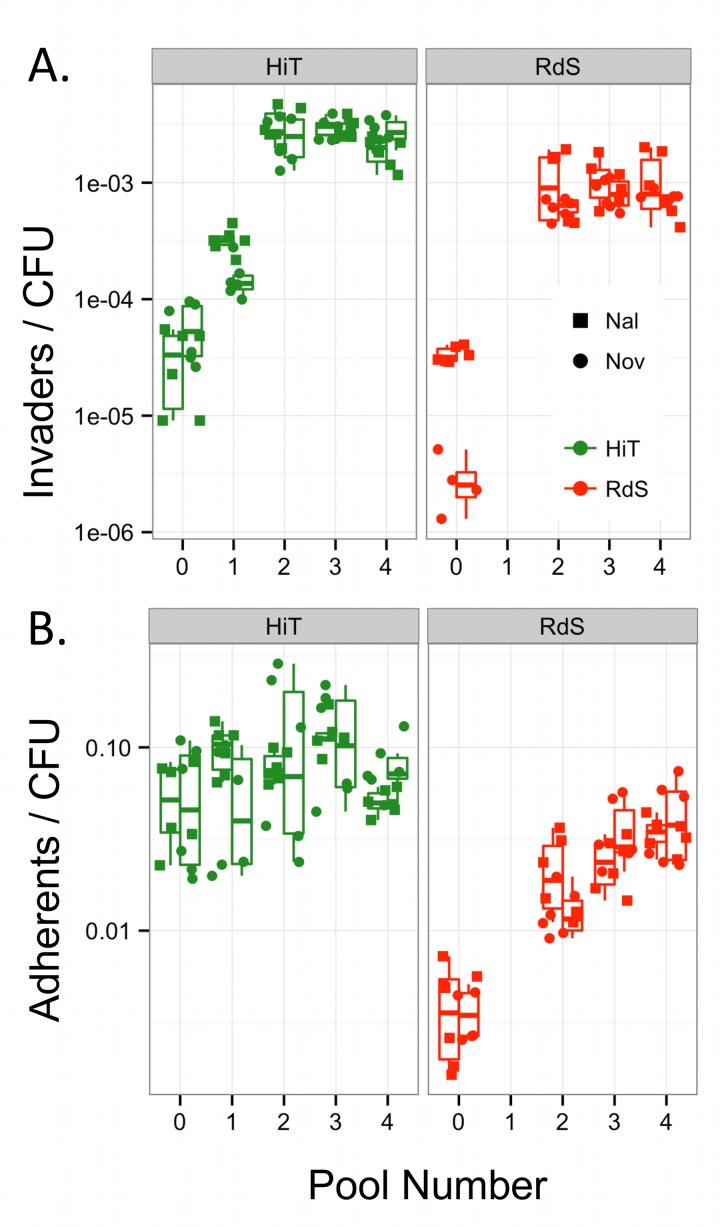
Serial enrichment for invasive recombinants by gentamicin protection. **(A)** Boxplots of intracellular invasion frequencies from Pools 0–4 (2 experiments in triplicate). **(B)** Boxplots of adhesion frequencies (2 experiments in triplicate). The values show the combined ability of clones in Pool *n* to invade or adhere to airway epithelial cells, while the recovered colonies comprise Pool *n+*1. Nal^R^ and Nov^R^ pools are summarized by the left and right boxplots, with individual data points shown as circles and squares, respectively.

The four initial recombinant pools (Pool 0s) were used in separate infections of A549 cells (Fig 1B). Serial passages of these pools using gentamicin protection resulted in >100-fold increased invasion after only three cycles of enrichment (S5 Fig). Replicate invasion assays using material from the initial enrichments confirmed the dramatic increase in intracellular invasion by pools after serial enrichment (Fig 3A, one-way ANOVA p<<0.001, Tukey’s HSD for comparisons of Pool 0 to Pools 2–4 were p<<0.001, but p>0.2 among Pools 2–4). Comparison with donor invasiveness values measured in parallel showed that the HiT recombinants in the two Pool 4s were not significantly less invasive than the donor (p = 0.082); the two Pool 4s using the RdS recipient remained marginally less invasive (p = 0.025). These data suggest that after the third serial enrichment (Pool 4), donor-specific genetic variants conferring invasiveness were at or near fixation.

Increased invasion was not due to selection for *de novo* mutations that confer increased invasiveness, nor due to changes in bacterial gentamicin sensitivity (controls detailed above). Such events were unlikely given that the number of cell generations across the experiment was <100 in total. Furthermore, a control experiment using untransformed RdS or HiT cultures found no significant increases in invasiveness over 5 serial cycles of selection with either recipient strain (S6 Fig; p>0.1 by one-way ANOVA), strongly suggesting that *de novo* mutations conferring increased invasiveness were not captured in these experiments.

Since higher adhesion might concomitantly increase intracellular invasion, we tested how adhesion was affected by the serial selections (Fig 3B). This found that the RdS pools showed substantial progressive increases in adhesiveness (one-way ANOVA p<<0.001, Tukey’s HSD p<<0.001 for comparisons of Pool 0 to Pools 2–4, but p > 0.2 for comparisons among Pools 2–4). The HiT pools trended towards increasing adhesion over serial enrichments, but for this experiment, no significant change was observed (p>0.1). Both pairs of pools still had significantly lower adhesiveness than NpNN cultures run in parallel (p<0.05 for all comparisons against NpNN).

In sum, RdS and HiT recombinants acquired loci or alleles that enhanced intracellular invasion. Adhesion increased, though to a lesser extent. This suggests that, while adhesion might be a prerequisite for invasion, its increase may be insufficient to explain the elevated invasion displayed by invasiveness-enriched recombinants.

### Serial enrichment for intracellular invaders selects for overlapping donor segments

Sequencing of genomic DNA across pools and serial passages (Fig 1C) showed that the four recombinant pools became progressively less complex, ultimately resulting in a total of only six recombinant clones dominating the four pools (out of ~4x10^5^ total). This suggests that the causative alleles transformed competent cells at lower rates than typical SNPs (~1,000-fold; S5 Table). The change in complexity was particularly apparent at the antibiotic-selected sites, where donor allele frequencies shifted from a smooth decline on either side of the resistance alleles to sharply demarcated donor segments (stretches of contiguous donor-specific variation) supporting the presence of only 1 or 2 antibiotic resistance-spanning segments dominating each pool (S7 Fig).

**Fig 4:**
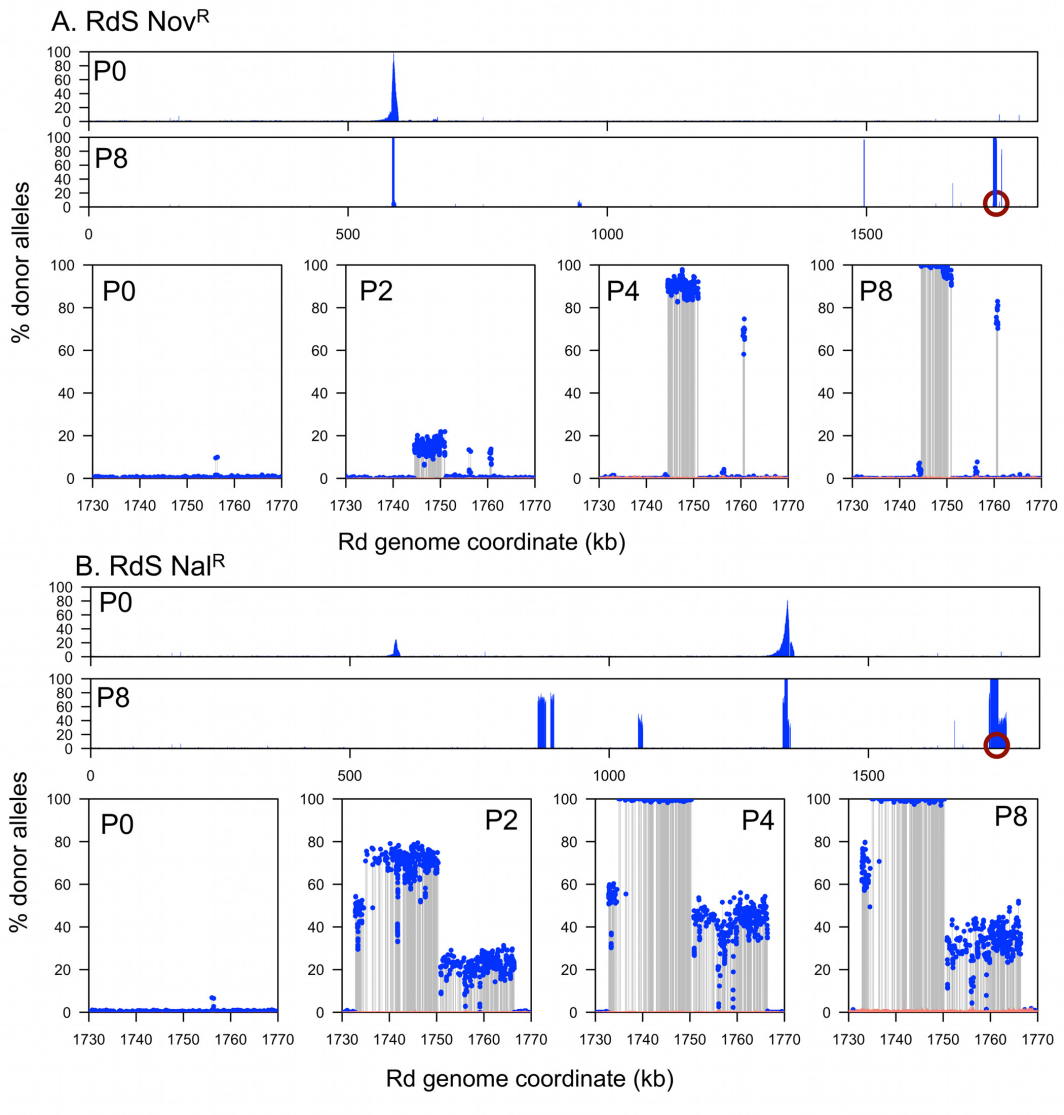
Genomic profile of the RdS recombinant pools. **(A)** RdS Nov^R^. **(B)** RdS Nal^R^. The x-axes indicate recipient genome coordinate, and the y-axes indicate the percent donor alleles. Blue dots and grey lines indicate donor-specific SNPs. Salmon color shows the limits of detection, or 1/depth (alignments with mapping quality = 0 were ignored, which excludes multiply mapping reads). Pools are indicated as P*n*. The two wide views at the top of **A** and **B** show the genome-wide donor allele frequencies for Pool 0 and Pool 8, whereas the lower four zoom around the nearly fixed locus selected for by serial gentamicin protection assay (indicated by purple circles) for Pools 0, 2, 4, and 8.

**Fig 5:**
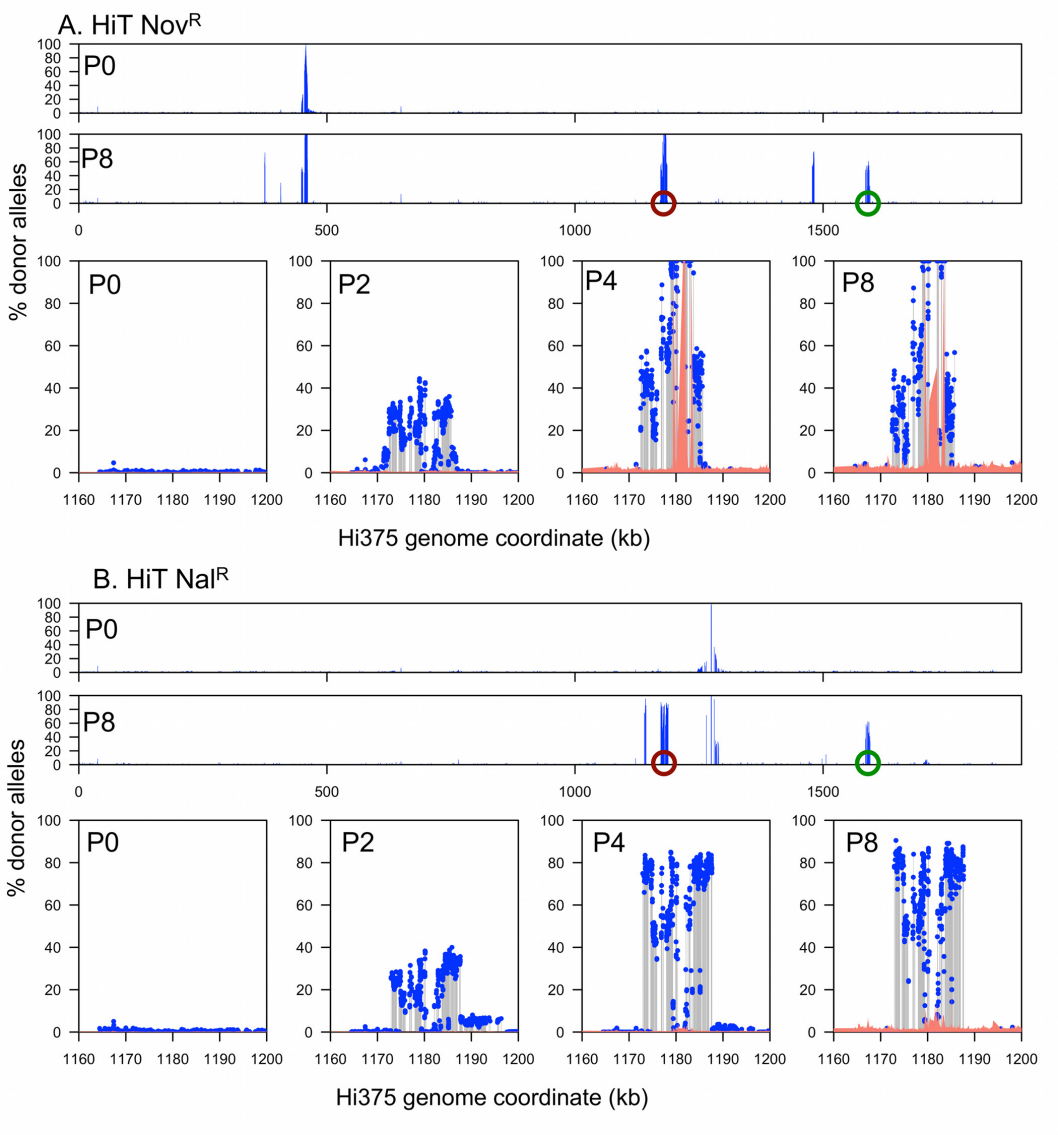
Genomic profile of the HiT recombinant pools. **(A)** HiT Nov^R^. **(B)** HiT Nal^R^. Plotting as in Fig 4, with the green circles indicating a multi-mapping artifact. Low limits of detection (1/depth, salmon color) within the invasion locus are due to alignments with mapping quality = 0 (multiply mapping reads) caused by the same sequencing artifact.

Coincident with decreasing complexity at the antibiotic-selected loci, serial enrichment also increased the frequency of donor segments in other intervals (Figs 4 and 5). At the end of selection, 1–2 recombinant clones dominated each pool, each carrying several donor segments (one segment in each clone spanning the antibiotic resistance allele).

Overlapping donor segment intervals between independent recombinants and pools are the best candidates for carrying invasion loci (*i*.*e*. the purple circles in Figs 4 and 5). In principle, donor-specific genetic variation found in only some clones (those seen with intermediate frequencies in the pools) could potentially modulate intracellular invasion, but “hitchhiking” segments that are not associated with invasion are expected to occur, since previous work has shown that competent cells typically take up and recombine multiple donor DNA molecules. Similarly, independent recombination tracts carrying the same invasion locus are expected to typically have independent recombination breakpoints [8,10].

### All invasion-enriched RdS recombinants had acquired the *hmw1*_*86-028NP*_ operon

**Fig 6:**
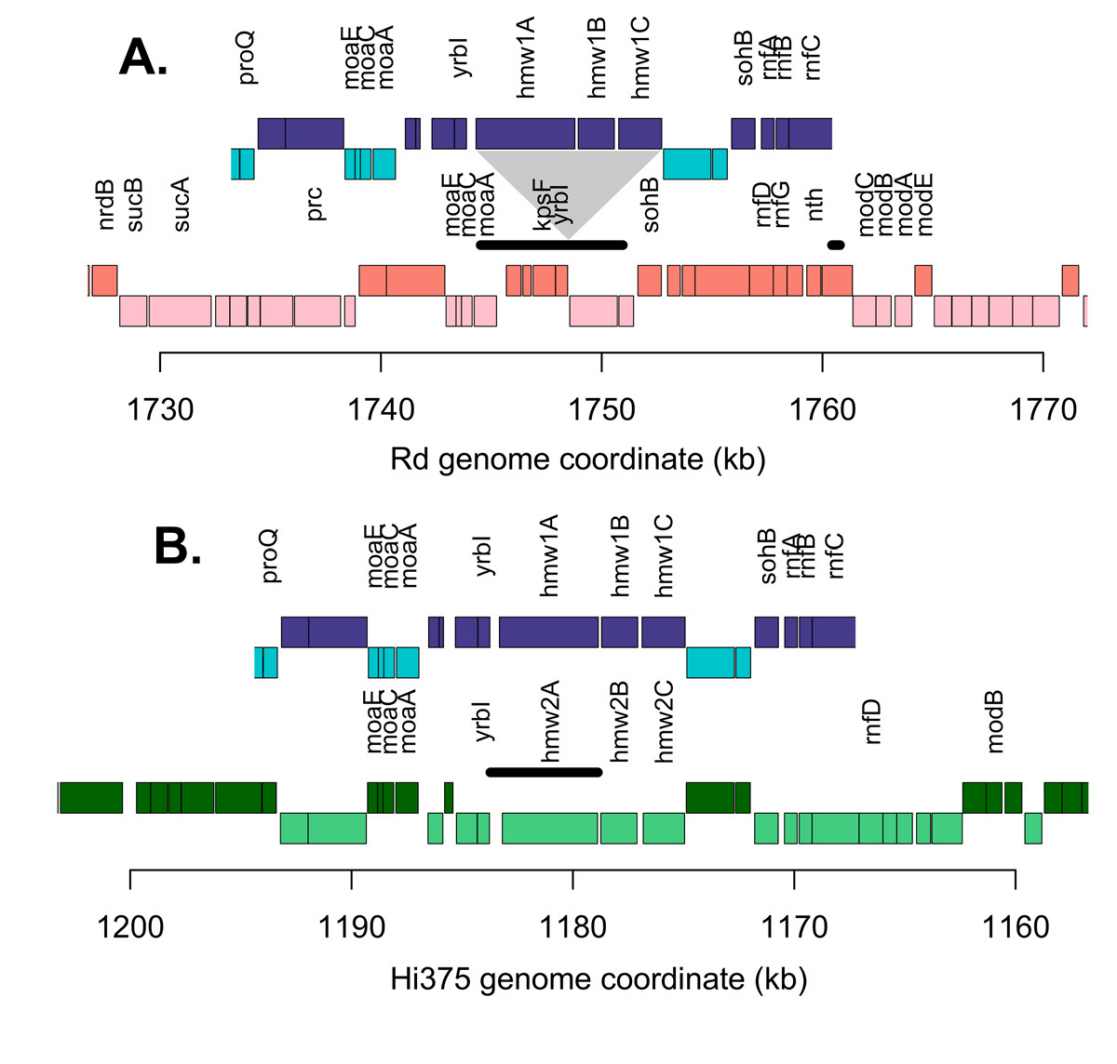
Genomic map at the enriched invasion locus for the three parental strains. For A and B, blue boxes indicate 86-028NP annotations; red is for Rd annotations, and green is for Hi375 annotations. Darker coloring indicates that sense is on the top strand, whereas lighter indicates bottom strand. Genes with names are indicated. The thick black horizontal lines indicate the minimum interval at or near fixation in both the Nov^R^ and Nal^R^ recombinant pools. **(A)** RdS recipient. Triangle indicates site of the *hmw1* insertion. **(B)** HiT recipient.

Sequencing of invader-enriched recombinant pools identified a single donor locus that was enriched to near-fixation in all four TREP experiments: *hmw1*_*86-028NP*_ (Fig 6). For the RdS recipient, a narrow interval reached near-fixation for both Nov^R^ and Nal^R^-selected pools (Figs 4 and 6A; Rd coordinates 1,744,519–1,750,336 nt, accompanied by a short nearby interval at 1,760,431–1,760,794 nt with lower levels of enrichment). The Rd gene annotations in this interval have no obvious connection with host cell interactions, but the donor strain carries a large operon here that is absent from Rd: *hmw1ABC*, which is found in ~60% of *H*. *influenzae* strains [40–43] and located between *yrbI* and *HI1680* (also known as *NTHI1986* in the 86-028NP genome). Depth-of-coverage analysis and reciprocal mapping of sequence reads to the donor genome confirmed the insertion of *hmw1ABC*_*86-028NP*_ into the invasion-enriched RdS recombinant pools. Three independent recombination-mediated insertions of *hmw1ABC*_*86-028NP*_ dominated the two invasion-enriched pools: one recombinant clone in the Nov^R^ pool and two in the Nal^R^ pool, as indicated by the distinct recombination breakpoints flanking the insertion (Fig 4). Additional recombination tracts were detected distant from the putative invasion locus and the antibiotic resistance marker, but these were unique to one of the three Pool 4 clones, as expected for random “hitchhiking” recombination events.

### Invader-enriched HiT recombinants had substituted their *hmw2A*_*Hi375*_ allele with the donor allele *hmw1A*_*86-028NP*_

Selecting for invaders from the HiT recombinant pools likewise enriched for donor segments containing *hmw1*_*86-028NP*_ adjacent to the *yrbI* gene (Figs 5 and 6B, S9 Table). Only two donor-specific intervals reached fixation in HiT Nov^R^: one spanning *gyrB* as expected, and another that completely spanned only the *hmw1A*_*86-028NP*_ gene carrying only short segments of the flanking genes (Hi375 genomic coordinates 1,178,771–1,183,728 nt). In HiT Nal^R^, a long donor segment spanning this same interval reached ~75%.

In contrast to RdS, the HiT recipient already possesses the *hmw1* operon and its paralogous operon *hmw2*, but the locations of the two adhesin genes (*hmw1A* and *hmw2A*) are swapped relative to their location in the donor NpNN. This is made evident through a comparison of the binding domains at the two *hmw* adhesin-encoding loci in HiT, NpNN, and the prototypic HMW-positive strain 12 (the strain that HMW adhesins were originally identified in and where they have been most extensively characterized, *aka* R2846) (Table 1). In strain 12 and the donor NpNN, *hmw1* is adjacent to the *yrbI* gene (*NTHI1982*) and *hmw2* is nearby the *radA* gene (*NTHI1453*). However, for Hi375, the *radA*-adjacent *hmw* adhesin has a binding domain with 100% amino acid identity to the *yrbI*-adjacent *hmw1A* gene from strain 12. Thus, whereas the *hmw1*_*86-028NP*_ operon was inserted into invasion-selected RdS recombinants, recombination events that increased HiT invasiveness were the result of an allelic substitution of *hmw2*_*Hi375*_ for *hmw1*_*86-028NP*_.

**Table 1:** HMW adhesin paralog assignment.

Locus	Strain	HMW1_strain12_	HMW2_strain12_	Best Hit
YrbI- adjacent	Strain 12	*	33.8%	HMW1
	86-028NP	39.8%	34.8%	HMW1
	Hi375	35.8%	50.7%	HMW2
RadA- proximal	Strain 12	33.8%	*	HMW2
	86-028NP	32.8%	55.4%	HMW2
	Hi375**	100.0%	33.8%	HMW1

Percent amino acid identity of six HMW binding domains to the prototype HMW1 and HMW2 paralogs in Strain 12, based on ClustalW2 alignments. Shared alignment gaps were excluded from pairwise comparisons. Self-alignments are indicated by *. The binding domains were defined by Strain 12 sequences as amino acids 555–914 in HMW1A_strain12_ and 553–916 in HMW2A_strain12_.

** The NCBI PGAAP annotation for *hmw1A*_*Hi375*_ ends with a serine instead of a stop codon and indicates a pseudogene. However, western analysis confirms its expression, and the presence of a stop codon 804 bp downstream accompanied by the expected pair of conserved C-terminal cysteine residues (codons 1624 and 1634) correctly defines the *hmw1A*_*Hi375*_ CDS as from coordinates 1,585,978 to 1,590,906 and encoding a 168 kDa protein.

The presence of the paralogous *hmw* locus nearby the *radA* gene caused an alignment artifact from multiply mapping sequence reads (Hi375 coordinates ~1,585–1,595 kb). Highly variable donor allele frequencies were seen across this interval ranging from ~0% to ~50% in Pool 8 for both HiT pools (S8 Fig). Furthermore, no donor-specific variation was detected flanking the *radA*-adjacent *hmw1A*_*Hi375*_ locus, in contrast to donor variation flanking the recombinant *yrbI*-adjacent *hmw1A*_*86-028NP*_ locus, which is expected for *recA*-mediated homologous recombination. Conclusive evidence for *radA*-proximal donor variation as a read alignment artifact is provided by allele-specific PCR assays on the isolated clones and mutants, as described below (S9 Fig).

Collectively, these data strongly support a role for *hmw1*_*86-028NP*_ in the increased intracellular invasion seen in enriched recombinants, though they do not strictly rule out a role for flanking donor-specific variation, particularly in the *yrbI* gene, since donor-specific variation in this gene was also near fixation in the invader-enriched recombinant pools. Comparison of sequence variants called between Pool 0s and Pool 8s identified that no novel mutations were fixed during serial selections, consistent with low per base mutation rates and the control experiment with untransformed recipients shown in S4 Fig.

### Individual recombinant clones validate and disambiguate the pool sequencing

**Fig 7:**
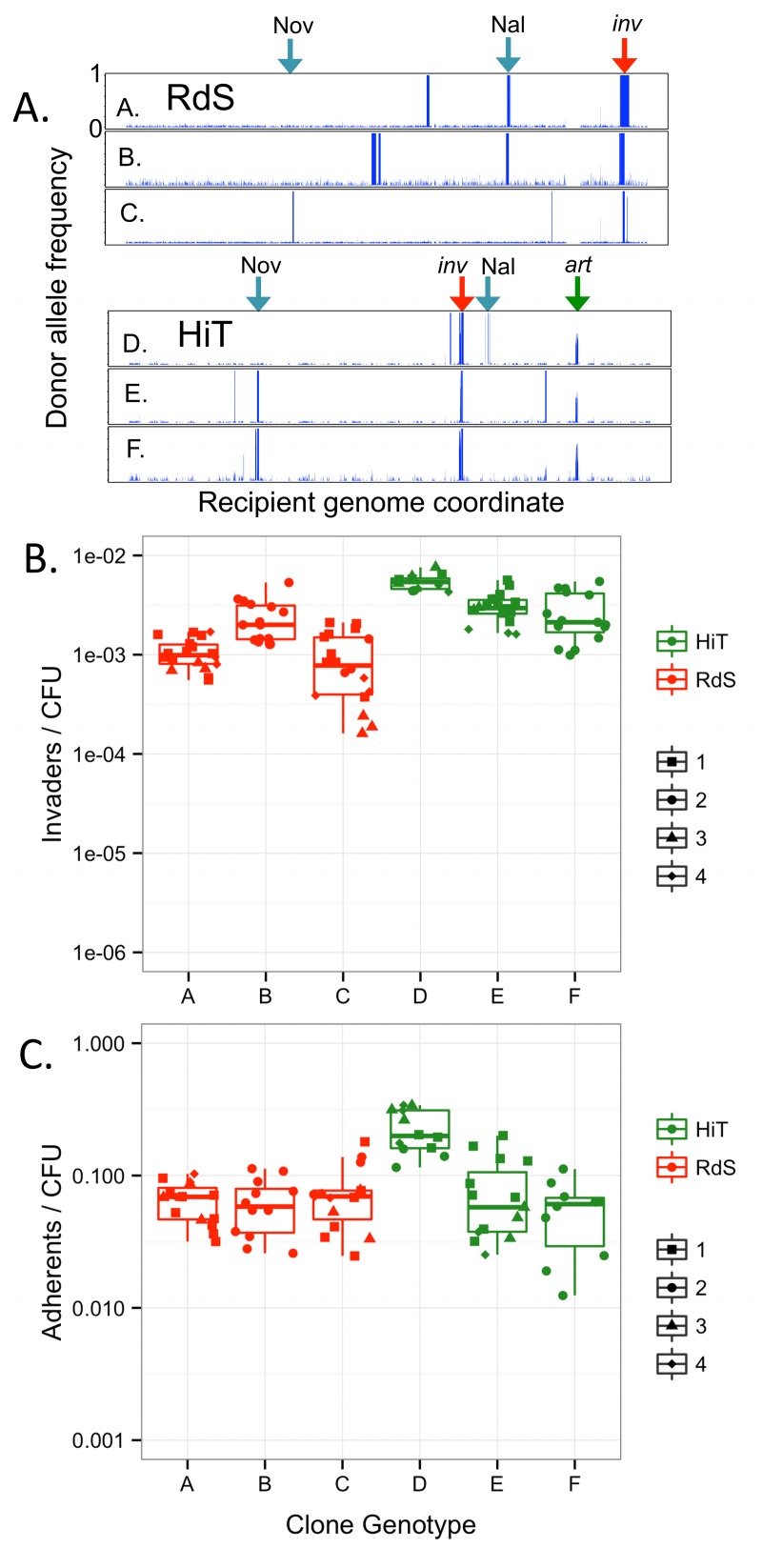
Genotype and phenotype of recombinant clones from Pool 4. Sequencing grouped 16 clones into six genotypes, two RdS Nal^R^ (A and B), one RdS Nov^R^ (C), one HiT Nal^R^ (D) and two HiT Nov^R^ (E and F). Genome-wide donor allele frequencies of each clone are shown in **(A)**. The x-axis indicates the recipient genome coordinate and the y-axis shows donor allele frequency from 0 to 1. Blue arrows mark the antibiotic resistance sites; red arrows mark the invasion locus; and the green arrow marks the *hmw2* artifact. Clone assignments are in S8 Table and exact donor segment breakpoints are in S9 Table. All clones were phenotyped in triplicate and were aggregated by genotype for plots depicting their invasion **(B)** and adhesion **(C)** phenotypes. Different symbols within each genotype indicate the specific isolated colony tested (labeled 1–4, as in S8 Table).

**Fig 8:**
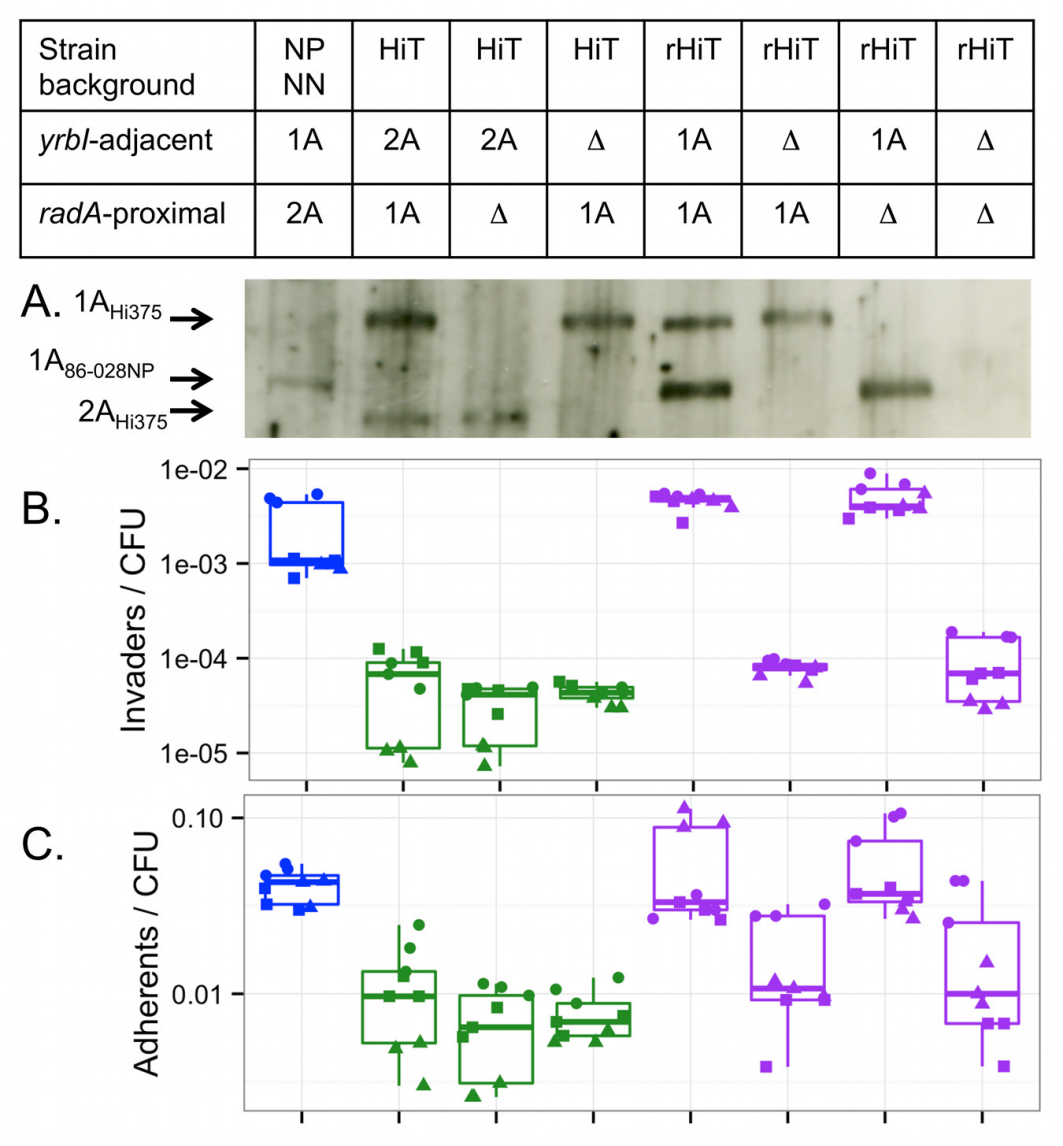
The *hmw1A*_*86-028NP*_ gene confers increased adhesion and intracellular invasion. **(A)** Western blot detection of HMW adhesins with a guinea pig anti-HMW1A gp85 antibody. The 154 kDa protein corresponds to HMW1A_86-028NP_; the 168 kDa protein corresponds to HMW1A_Hi375_, and the 150 kDa protein corresponds to HMW2A_Hi375_. Expression of HMW2A_86-028NP_ was not detectable with the antibody used. **(B)** Intracellular invasion and **(C)** adhesion by NpNN and HiT derivatives including mutant, recombinant and recombinant mutant strains. Blue indicates NpNN, green indicates the HiT recipient and mutant derivatives, and purple indicates the rHiT recombinant and mutant derivatives. Experiments were run in triplicate on three separate days, as indicated by the three distinct symbols.

Four colonies from Pool 4 were isolated from each of the four TREP experiments and further analyzed. Each of the 16 clones was assayed in triplicate for its invasion and adhesion phenotypes (Fig 7). Subsequent genome sequencing (summarized in S3 Table) revealed a total of only six distinct genotypes: three clones from each recipient background (Fig 7A; S9 Table). This is consistent with predictions from the pool data and shows that the isolated clones represented all the high frequency recombinants observed in Pool 4. Intracellular invasion and adhesion were strongly enhanced for all 16 isolated clones compared to recipient controls (Fig 7B and 7C, Tukey’s HSD p<<0.001 for all six comparisons). Immunoblot analysis confirmed expression of HMW1A_86-028NP_ protein in two recombinant clones, RdS genotype B (S10A Fig) and HiT genotype E (Fig 8A).

Not all of the recovered genotypes had identical phenotypes. Genotype D colonies (HiT Nal^R^ from Pool 4) were significantly more invasive and adherent than genotype E and F colonies (one-way ANOVA p<<0.001, Tukey’s HSD gives p<0.001 for comparison of D against E or F, but p>0.1 for comparison of E and F). This indicates that donor-specific variation present in genotype D but absent from genotypes E and F slightly enhances adherence and invasion, albeit substantially less so than the *hmw1ABC* locus; the causative variation responsible remains unknown, but includes donor alleles of a QseBC-like two-component system (S9 Table, “D-specific” segments).

The sequencing of Pool 4 clones further supported a read alignment artifact at the *radA*-adjacent *hmw1A*_*Hi375*_ locus. Colonies had been collected after re-streaking to ensure they represented single clonal lineages. Despite this, all three HiT clone genotypes (D, E, and F) from Pool 4 showed a variable mixture of recipient- and donor-specific alleles across the *radA*-proximal *hmw* operon (Fig 6). In contrast, donor-specific allele frequencies observed at the *yrbI*-adjacent *hmw1A*_*86-028NP*_ were near fixation and accompanied by flanking donor-specific variation. As a final confirmation that this mixed signal was not due to merodiploidy or other complex genetic effect, allele-specific PCR assays were conducted that distinguished all four *hmw* adhesin genes (the *yrbI*-proximal *hmw1A*_*86-028NP*_ and *hmw2A*_*Hi375*_ genes and the *radA*-proximal *hmw2A*_*86-028NP*_ and *hmw1A*_*Hi375*_ genes), and these PCR assays unambiguously showed that HiT recombinants had replaced their *yrbI*-adjacent *hmw2*_*Hi375*_ allele with the *hmw1*_*86-028NP*_ allele, whereas the *radA*-adjacent *hmw1*_*Hi375*_ alleles were unchanged (S9 Fig).

### Donor-specific variation in the *hmw1ABC* left flanking interval does not contribute to enhanced invasiveness

Despite the unambiguous acquisition of *hmw1*_*86-028NP*_ in all recombinant clones, other donor-specific variation in the recombinant clones could conceivably be responsible. Since recombination tracts that carried *hmw1ABC*_*86-028NP*_ also carried flanking donor-specific SNPs, we directly tested for a contribution by variation in the flanking interval. We cloned the 86-028NP alleles of the two genes upstream of *hmw1ABC*: *kpsF* and *yrbI*, encoding arabinose-5-phosphate isomerase and Kdo 8-phosphatase, respectively. Donor-specific variation in *yrbI* in particular might contribute to intracellular invasion, since the minimum interval that overlapped between all four experiments contained donor-specific variation in this gene (Fig 6). The resulting HA-tagged pSU20 plasmid (pSU20-*kpsF-yrbI*-HA) was then electroporated into Rd. Confirming expression from the plasmid, a ~19.3-kDa full-length YrbI_*86-028NP*_-HA protein was detected in whole cell extracts by immunoblot with an anti-HA antibody (S10B Fig). Strains Rd, Rd pSU20, and Rd pSU20-*kpsF-yrbI*-HA were tested for intracellular invasion into A549 cells. No significant difference was observed among strains (S10C Fig, one-way ANOVA p-value = 0.29), thereby excluding a significant role for these flanking loci in intracellular invasion.

### Mutation of *hmw1A*_*86-028NP*_ confirms its role in intracellular invasion

Genetic confirmation of a role for *hmw1A*_*86-028NP*_ in intracellular invasion (rather than other donor-specific variation acquired by recombinants) was performed using the HiT recipient and one of the invasive HiT recombinant clones (strain P551 with genotype E, hereafter called rHiT). We generated a panel of knockouts of the genes encoding HMW adhesins in the HiT and rHiT strains, either the locus adjacent to *yrbI* (*hmw2A*_*Hi375*_ or *hmw1A*_*86-028NP*_) or the locus nearby *radA* (*hmw1A*_*Hi375*_). In the case of rHiT, the double mutant was also produced (both *hmw1A*_*86-028NP*_ and *hmw1A*_*Hi375*_ deleted). Western blot analysis confirmed expression of the expected HMW adhesins in each strain (Fig 8A).

All mutant strains were assayed for invasion in parallel with NpNN, HiT, and rHiT controls (Fig 8B, one-way ANOVA p<<0.001). We hypothesized that knocking out *hmw1A*_*86-028NP*_ in the recombinant rHiT would show a strong defect in intracellular invasion, but that knocking out either *hmw* gene in the HiT recipient would have little or no effect. Indeed, deletion of *hmw1A*_*86-028NP*_ from rHiT reduced invasion frequencies 56-fold (Tukey’s HSD p<<0.01) down to HiT recipient levels. By contrast, deletion of the *radA*-proximal *hmw1A*_*Hi375*_ had no significant effect in either strain background, nor did deletion of either locus in the HiT recipient (Tukey’s HSD p>0.5 for all comparisons).

These results confirmed the role of *hmw1A*_*86-028NP*_ in the increased intracellular invasion of the HiT recombinant, and suggested that the HiT alleles of the HMW adhesins do not appreciably contribute to the HiT strain’s ability to invade A549 cells. Parallel adhesion assays of the knockout panel found qualitatively similar results (Fig 8C, one-way ANOVA p<<0.001). The recombinant rHiT was significantly more adherent than the HiT recipient (4.7-fold increase, Tukey’s HSD p<0.001)—comparable to adhesion by the donor NpNN (p = 0.99)—whereas deletion of *hmw1A*_*86-028NP*_ from rHiT brought adhesion down 3.3-fold to HiT recipient levels (Tukey’s HSD p = 0.0014 versus rHiT, p = 0.99 versus HiT).

The effect of *hmw1A*_*86-028NP*_ on adhesion is >10-fold lower than its effect on intracellular invasion, such that significant increases in adhesion had not been detected in the original pool experiments and were only marginally significant in adhesion assays with the isolated recombinant clones (see above). Nevertheless, these results indicate that *hmw1A*_*86-028NP*_ might contribute to intracellular invasion in part through an indirect effect of increasing adherence. While this is one contributing factor, the immunofluorescence data reported below indicate an unexpected intracellular invasion phenotype that cannot readily be explained by increased adherence alone.

### Possession of *hmw1ABC*_*86-028NP*_ confers a self-aggregation phenotype to recombinants

**Fig 9:**
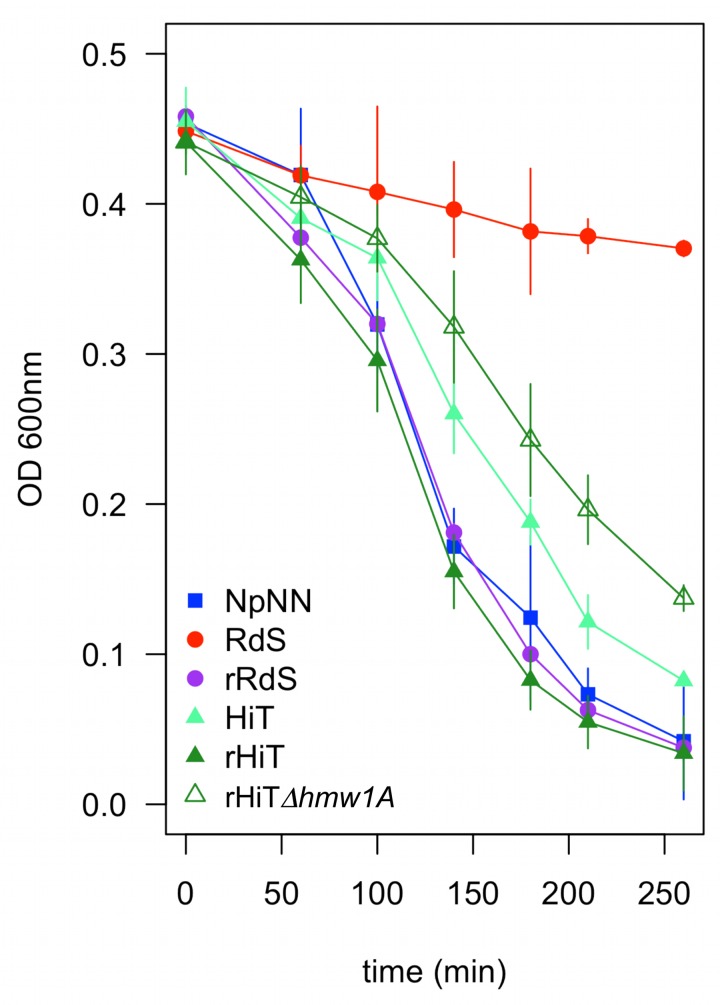
Self-aggregation is increased by *hmw1A*_*86-028NP*_. Bacteria scraped from chocolate agar plates were suspended into 35 ml of sBHI normalized to OD_600_ = 0.5 in a 50 ml conical tube and allowed to sit on the lab bench. The OD_600_ at the top of the cultures was followed over time as a proxy for clumping/self-aggregation. Error bars indicate the standard deviation from four replicate assays run on different days.

In the course of working with the parental strains, we noted that when cultures were left standing on the bench, NpNN (and 86-028NP) settled more quickly than RdS (and Rd), denoting a clumping or self-aggregation phenotype. Because self-aggregation could modulate bacterial-host cell interplay, we quantitatively tested this phenotype for a panel of strains: the three parents, an invasive recombinant RdS clone (P540, genotype B, hereafter rRdS), the rHiT recombinant, and the rHiTΔ*hmw1A*_*86-028NP*_ mutant. This clearly demonstrated that *hmw1A*_*86-028NP*_ plays a major role in the high self-aggregation seen in the recombinants (Fig 9; one-way ANOVA p<<0.001 at the t = 140 min time point and higher). Both recombinants settled quicker than the recipient strains (Tukey’s HSD p<0.001) but were indistinguishable from NpNN (p>0.6). While the HiT recipient settled substantially faster than RdS, it and the rHiTΔ*hmw1A*_*86-028NP*_ mutant settled slower than NpNN (p<0.01). These observations raised the question of how this clumping or self-aggregation phenotype modulates adhesion and intracellular invasion.

### Immunofluorescence microscopy reveals that *hmw1ABC*_*86-028NP*_ confers a novel aggregated intracellular bacterial invasion phenotype

**Fig 10:**
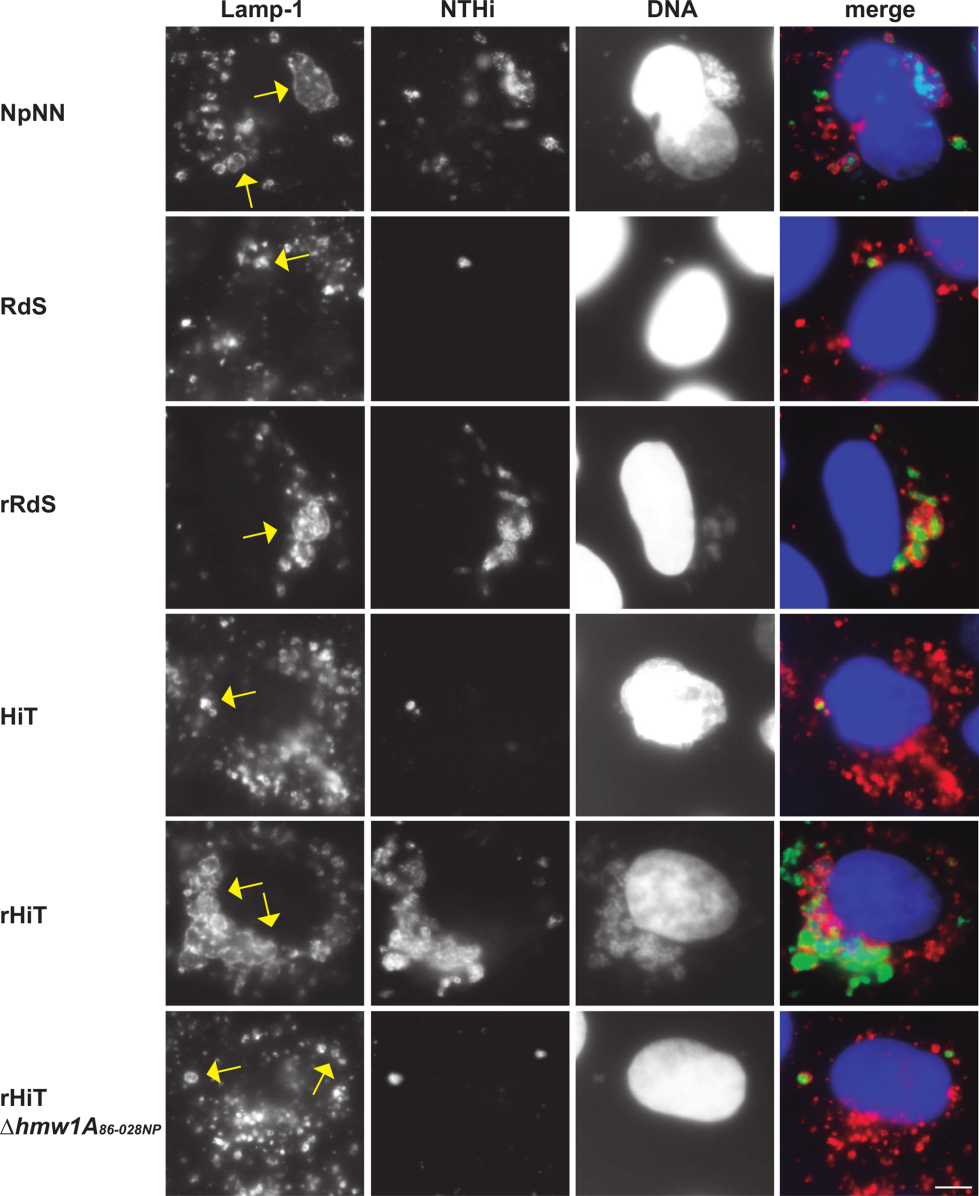
Co-localization of intracellular bacteria and the Lamp-1 endosomal marker. A549 cells were infected by NpNN, RdS, rRdS, HiT, rHiT, or rHiTΔ*hmw1A*_*86-028NP*_. In the merged images, reactivity with an anti-NTHi antibody is shown in green, Lamp-1 stain is shown in red, and DNA stained with Hoechst 33342 is shown in blue. Images were taken at 1 h post-gentamicin treatment. The scale bar in the lower right indicates 5 microns; individual bacterial cells are ~1 micron in diameter.

To directly assess intracellular location of bacterial cells, we used immunofluorescence microscopy (Fig 10). As expected, the donor (NpNN) and two recombinant (rRdS and rHiT) strains infected A549 cells at high rates, whereas the recipients (RdS and HiT) and the mutant recombinant rHiTΔ*hmw1A*_*86-028NP*_ infected A549 cells at substantially lower rates (Table 2). Co-localization with the endosomal marker Lamp-1 was substantial for all strains, indicating *bona fide* intracellular invasion, rather than gentamicin resistance or occlusion by A549 cells. These results confirm: (a) that both recipients (including RdS) successfully enter A549 cells, albeit at low rates; (b) that donor and recombinants have substantially higher invasion rates than recipients; and (c) that *hmw1A*_*86-028NP*_ is responsible for elevated intracellular invasion rates in the rHiT recombinant.

**Table 2:** Intracellular bacteria immunofluorescence microscopy.

Strain	% infected (n = 250 cells)^a^	% bacteria:Lamp-1 co-localization (n = 150 infected cells)	% cells with # of infecting bacteria (n = 250 infected cells)	
			<10	>10
RdS (P532)	11.6	74.1	100	0
HiT (P531)	33.6	42.0	98.8	1.2
NpNN (P351)	100	88.0	0	100
rRdS (P540)	90.0	87.6	61.3	38.7
rHiT (P551)	80.8	93.9	76.2	23.8
rHiTΔ*hmw1A*_*86-028NP*_ (P834)	44.7	65.3	92.1	7.9

^a^Frequencies are higher than from the plating assays above due to bacterial lysis during the saponin treatment that lyses A549 cells, as previously described [24].

The number of bacteria per infected cell was also distinct between strains. Cells infected by either recipient or the rHiTΔ*hmw1A*_*86-028NP*_ mutant were infected with <10 bacteria/cell, whereas cells infected by the donor strain had >10 bacteria/cell. Although visually similar to the NpNN donor, scoring indicated that the rRdS and rHiT recombinants had an intermediate phenotype (Fig 10, Table 1). Whereas gentamicin protection assays with the recombinants gave invasion rates that approached donor levels, these data suggest that additional unidentified donor-specific factors besides *hmw1A*_*86-028NP*_ may augment the ability of bacteria to invade.

Unexpectedly, bacterial invaders had a distinct pattern of co-localization with Lamp-1-positive endosomes for the donor and two recombinants. When infected by bacteria of either recipient strain or the rHiTΔ*hmw1A*_*86-028NP*_ mutant, Lamp-1-positive subcellular compartments typically enclosed single bacterial cells, as previously observed [24]. In contrast, cells of the donor or either recombinant had enlarged Lamp-1-positive subcellular compartments that surrounded groups of bacteria. The size of these groups varied, with ~5–50 bacteria per compartment (Fig 10).

These results indicate that increased intracellular invasion by the donor and recombinants relates—at least in part—to internalized bacteria localizing as groups in the same subcellular compartment, rather than as individual cells. Previous work showed that *H*. *influenzae* invaders do not replicate [24] and, in our assays, the time between infection and observation was sufficiently brief that the groups of intracellular bacteria seen are unlikely to reflect intracellular replication. Hi375 has previously been seen to clump at the surface of A549 cells prior to invasion, but with only single bacteria undergoing internalization [24]. Thus, our self-aggregation and immunofluorescence results strongly suggests that groups of *hmw1ABC*_*86-028NP*_ bacteria remain clumped during epithelial cell entry, thereby increasing overall invasion rates above and beyond the indirect effect of elevated adhesion, and also explaining the observed Lamp-1 reorganization around groups of bacteria.

### Addition of *hmw1ABC*_*strain12*_ to Rd increases intracellular invasion

**Fig 11:**
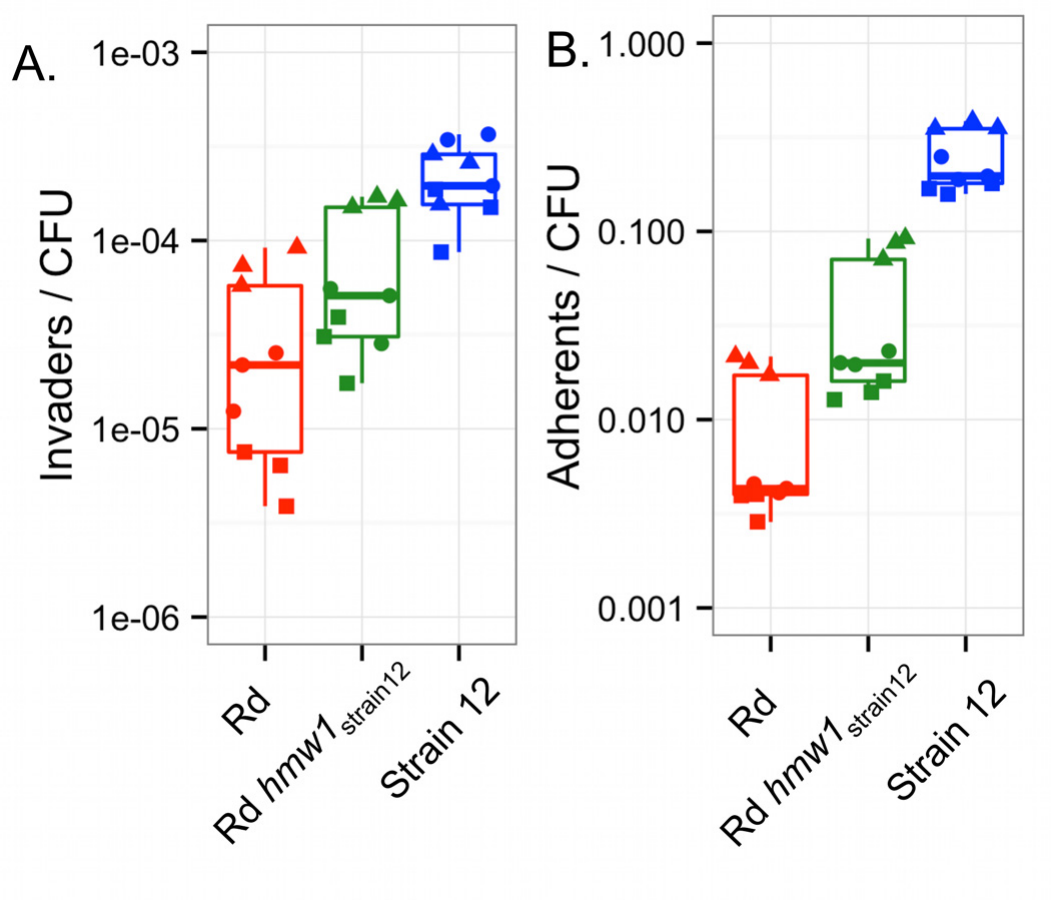
The *hmw1*_*strain12*_ allele confers increased intracellular invasion to Rd KW20. (**A**) Invasion and (**B**) adherence phenotypes of Rd, Rd *hmw1*_*strain12*_, and strain 12 were evaluated in A549 cells in triplicate on three separate days (shown by distinct symbols).

Previous studies of *hmw1* focused on its role in adhesion, including detailed characterization of an Rd derivative carrying the *hmw1* operon from strain 12 with only minimal flanking variation from strain 12 [44]. We used the Rd *hmw1*_*strain12*_ strain to independently test for the role of *hmw1* in intracellular invasion. We evaluated invasiveness and adhesiveness of Rd, Rd *hmw1*_*strain12*_, and strain 12 using A549 cells, as described above. Rd *hmw1*_*strain12*_ had intermediate levels of both invasion and adhesion between both parents (Fig 11, p-values<0.01 for one-way ANOVA and all three comparisons by Tukey’s HSD).

To test how these results depended on the specific protocol or cell type used, we evaluated invasion and adhesion following an alternative protocol in both A549 and Chang cells [18]. Rd *hmw1*_*strain12*_ had significantly higher adhesion and invasion than Rd for both cell types, though the effect was much stronger with Chang cells (S11 Fig, p-value < 0.01 for all three comparisons, except p = 0.054 for adherence to Chang cells by Rd *hmw1*_*strain12*_ and Strain 12). Rd *hmw1*_*strain12*_ invaded Chang cells nearly as well as strain 12. In contrast, when infecting A549 cells, Rd *hmw1*_*strain12*_ had an intermediate phenotype. These results indicate that *hmw1*’s contribution to invasion depends on the host cell type and confirm a more general role for *hmw1* in intracellular invasion beyond that seen for the 86-028NP allele.

## Discussion

### Is *hmw1* directly involved in *H*. *influenzae* intracellular invasion?

In contrast to bacterial pathogens with well-characterized intracellular life styles like *Salmonella*, *Listeria*, *Legionella* or *Brucella* [45–47], the mechanism and functional role of intracellular invasion in *H*. *influenzae* has been less well understood. It has been suggested that intracellular invasion of airway epithelial cells by non-typeable *H*. *influenzae* (NTHi) allows the bacterium to evade the immune system (antibodies, surfactant, antimicrobial peptides, galectins, professional phagocytes) and therapeutic interventions (antibiotics, anti-inflammatory agents), and to facilitate access to essential nutrients [14,48–50]. Thus, entry into airways cells may better equip bacterial cells for survival during long-term infections, particularly in the context of chronic infections that are often treated with intense antibiotic regimes.

Potential factors that contribute to invasion include those known to facilitate *H*. *influenzae*’s interactions with host cell surfaces. Among these are bacterial surface proteins that participate in *H*. *influenzae* binding to extracellular matrix proteins, mucin, or epithelial cells (including P5, OapA, PE, Hap, Hia, the HMW1 and HMW2 adhesins, IgA1 proteases A and B, and type IV pili [26,33,38,51–54]). Some factors, such as IgaA1 protease, appear to be more directly involved in *H*. *influenzae* entry into epithelial cells [26]. The PE and Hap adhesins have also been implicated in *H*. *influenzae* entry into epithelial cells [55,56]. While adherence to host cells may be a prerequisite for invasion, we lacked information on the specific involvement of adhesins or other factors that modulate intracellular invasion by *H*. *influenzae*. TREP was designed to identify invasion-promoting genes in an unbiased manner, but we were nonetheless surprised to isolate the well-characterized adhesin-encoding *hmw1* operon.

Gain of *hmw1*_*86-028NP*_ by a poorly invading strain naturally lacking both *hmw1* and *hmw2* (RdS) dramatically enhanced both adhesion and invasion, showing that *hmw1*_*86-028NP*_ is sufficient to confer high adhesion and invasion levels. When a recipient strain already possessing both *hmw1* and its paralog *hmw2* (HiT) was transformed, allelic substitution of the *hmw2A*_*Hi375*_ allele with *hmw1A*_*86-028NP*_ also strongly enhanced invasion. Characterization of recombinant clones and mutants lacking functional *hmw1*_*86-028NP*_ confirmed these results. Important roles for other donor-specific variation carried by the transformed recombinants can be excluded, except that one of the HiT recombinants (genotype D) showed significantly higher adhesion and invasion than the others, suggesting at least one additional invasion-promoting factor, albeit a smaller contributor; further analysis will be of interest in future studies.

**Fig 12:**
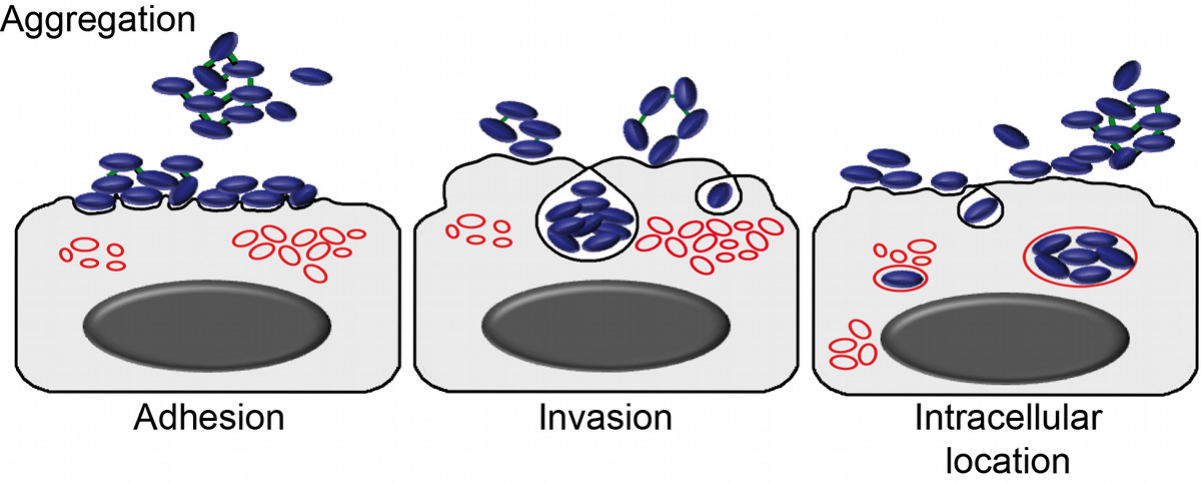
Model of enhanced intracellular invasion by HMW1_86-028NP_. Bacteria self-aggregate and adhere to the epithelial cell surface in clumps, and HMW1-mediated attachments are maintained during uptake by cells into subcellular compartments with endosomal features.

The striking intracellular phenotype we observed by immunofluorescence for *hmw1*_*86-028NP*_ strains—in which groups of bacteria occupy engorged intracellular vesicles—suggests that increased adhesion alone is insufficient to fully explain the role of *hmw1* in intracellular invasion. We instead suggest that elevated invasion is an emergent property of HMW1-mediated self-aggregation; whereas adhesion is increased as an indirect result, we speculate that invasion by bacterial groups directly enhances overall invasion rates. Alternatively, possession of *hmw1*_*86-028NP*_ may increase independent entry by bacteria into cells, followed by subsequent aggregation of bacterium-containing vesicles. Ruling out self-aggregation *per se*, recent results show that deletion of the Hap autotransporter from Hi375 (naturally present in both 86-028NP and Hi375 but absent from Rd) eliminates self-aggregation, but epithelial cell adhesion and invasion were not significantly affected [57]. Altogether, we propose that, whether or not it can truly be called an “invasin”, allelic variation in HMW1A affects invasion by increasing adhesion and also directly through a novel mechanism that allows for entry by groups of aggregated bacterial cells (model summary in Fig 12).

### The HMW1 and HMW2 adhesins

The *hmw1* and *hmw2* operons are found in ~60% of *H*. *influenzae* isolates, and they always co-occur, despite being at different chromosomal loci (one adjacent to *HI1679* in Rd and the other to *HI1598*) [40,58,59]. Co-occurrence of the *hmw1* and *hmw2* loci in all tested clinical isolates suggests that the laboratory-created *hmw1*-only strains studied here must be at some unknown fitness disadvantage in nature.

The *hmw1* and *hmw2* operons are phase-variable, and expression is inversely correlated with the number of 7-bp tandem repeats found within their promoter regions [60–63]. Though subtle expression variation was not ruled out, western blot analysis indicated that HMW1 adhesin levels were mostly unchanged in recombinants (Fig 8A; 16 repeats upstream of *hmw1*_*86-028NP*_ but 17 upstream of *hmw2*_*Hi375*_). This indicates that allelic variation in the *hmw* coding sequences is likely responsible for differences in adhesion and invasion.

HMW1A and HMW2A display wide amino acid diversity both within and between isolates, with the region of lowest sequence identity in the host cell binding domain, which has been predicted to affect tissue tropism and immune evasion [42,59,63–67]. Phylogenetic analyses of the HMW adhesin binding domain has revealed four distinct sequence clusters, and the majority of sequences belonging to one of two dominant sequence clusters [41]. Of note, 86-028NP and strain 12 *hmw1A* binding domains belong to clusters 4 and 2, respectively, which might contribute to their strong and intermediate phenotypes; future studies using mosaic proteins with binding domains from distinct clusters and using multiple human cell lines could identify any clade-specific functions for HMW proteins.

The two *hmw* operons encode high molecular weight non-pilus adhesins (HMW1/HMW2) [37,64], along with two co-factor proteins encoded by the downstream genes. The co-factors are required for proper surface localization of HMW adhesins, and the paralogs of the co-factors are functionally interchangeable [37,58]. The *hmw1B/hmw2B* genes encode outer membrane pore-forming translocators that export HMW1 and HMW2 to the cell surface [68]. The *hmw1C/hmw2C* genes encode glycosyltransferases responsible for adding mono-hexose or di-hexose residues at asparagines in conserved NX(S/T) motifs of HMW1 and HMW2 [69], likely involved in stabilizing the adhesins during or after their synthesis [70]. Due to the high diversity in HMW adhesin sequences, differential glycosylation patterns might in part be responsible for distinct activities of different alleles.

Another key distinction between the HMW1 and HMW2 adhesins is that the former recognizes sialylated glycoprotein receptors on cultured human epithelial cells [71]. HMW1 confers high adherence to Chang, Hep-2, HaCaT and NCI-H292 cells mediated by interactions with α-2,3 N-linked sialic acids. By contrast, HMW2 confers adherence to HaCaT and NCI-H292 cells via a sialic acid-independent mechanism [59,67,71]. This suggests that the role of *hmw1A*_*86-028NP*_ in intracellular invasion may involve specific interactions between *H*. *influenzae* cells and sialylated glycoprotein receptors, both on the bacterial cell surface to mediate self-aggregation and possibly also specific host sialylated glycoproteins on epithelial cells. Glycoproteins play important roles in many cellular activities, and new methods for investigating their expression and sialylation states are being developed and applied to multiple cell types including A549 [72], opening new avenues to identify host glycoproteins hijacked by bacterial proteins such as HMW adhesins during the infection process.

To our knowledge, this is the first report of an involvement for *hmw1A* in *H*. *influenzae* intracellular invasion and, more strikingly, we further found that intracellular invasion is modulated by allelic diversity at *hmw1A*. Finding that *hmw1*_*86-028NP*_ results in clumps of intracellular bacteria was unexpected and indicates that increased self-aggregation and adhesion *per se* are not sufficient to explain its effects, offering new avenues of investigation. Bacterial factors contributing to adhesion are already potential targets for antimicrobial therapies, and the additional role of HMW1 in intracellular invasion further increases its attractiveness as a target. Understanding the relationship between *hmw1* allelic variation and within-host adaptive evolution poses interesting challenges for future studies.

### Transformed recombinant enrichment profiling

To better understand intracellular invasion by *H*. *influenzae*, we have successfully employed a gain-of-function genetic mapping strategy, TREP, which takes advantage of within-species phenotypic variation, natural competence, and deep sequencing. In total, our experiment isolated six highly invasive recombinants from a total of ~400,000 independent recombinants. Thus, while transformation rates of SNPs was much higher (*e*.*g*. ~0.4% for the antibiotic resistance alleles), this approach was able to isolate even very rare recombinants, in the case of the Rd strain requiring the insertion of a particularly long operon (>9kb). TREP proved to be a rapid method to map genes. Once the donor and recipient were assayed and the effectiveness of the selections was determined, the total hands-on time was only about six weeks, from generating the recombinant pools, performing the serial selections, extracting DNA, making libraries and sequencing, with serial selections comprising the most time-consuming step. Owing to the strong selection used, we found that strains that have slight advantages in invasion were able to overtake the pools after serial selection. Thus, it was crucial to add selectable markers to our recipient background and to ensure that the serial selections were performed without the donor strain or any other strains assayed in parallel.

The TREP method holds great promise for studying a wide range of traits that show natural phenotypic variation in other naturally competent species, which includes many virulence traits and pathogens important to human health. In contrast to screening/selecting clones transformed by plasmids [73], TREP does not depend on dominance, a suitable vector, nor is it restricted to monogenetic traits. The approach should be readily applicable to any selectable trait in any bacterial species for which a natural competence protocol has been developed, and the number of such species continues to grow. Similar approaches have recently been reported in other organisms, for example to identify conjugation genes in *Mycobacterium* and causative alleles responsible for antibiotic resistance in *Streptococcus* [7,74]. Importantly, TREP is a general genetic mapping strategy agnostic to the type of variation (*i*.*e*. SNPs or whole loci can be identified), and we expand the utility of the transformation-based genetic mapping to include quantitative differences that go beyond absolute phenotypic differences (*i*.*e*. resistance *versus* sensitivity) by incorporating serial selection.

Traditionally, microbial experimental evolution studies rely on “hard” selective sweeps, in which newly arising beneficial mutations fix in a laboratory population [75]; more recently this has also included experimental evolution of pathogenic traits [76,77]. But “soft” sweeps, in which pre-existing genomic variation recombines within/into a population [78], may also play an important role in the adaptation of naturally competent species to new environments [79]. Allowing for introgression of natural variation has been used in experimental evolution in sexual eukaryotes (*e*.*g*. [80,81]), but its role in bacterial adaptation has been explored only recently and only in the context of standard experimental evolution studies that start with clonal populations [82,83]. Here, we found that rare recombinants generated in a single round of natural transformation could reach fixation after a small number of serial selections, illustrating the powerful contribution of natural competence to adaptation.

Finally, applying TREP to understand bacterial pathogenesis could use large “zoos” of donor genomic DNAs, rather than single donor-recipient combinations. This would better mimic the situation in chronic infections, where diverse polyclonal infections are common, and it would more fully sample the genomic diversity of these organisms in single experiments. However, caution must be exercised with such an approach: depending on the organism and trait under study, this could inadvertently generate novel hyper-virulent strains by combining multiple pathogenicity factors from different genetic backgrounds; a similar ethical concern has already been raised for studying pathogens using gain-of-function mutagenesis [84,85].

## Materials and Methods

### Bacterial strains

Bacterial strains and plasmids used are listed in S1 Table, and all PCR primers used are in S10 Table. General culturing and manipulation of *Haemophilus influenzae* followed standard methods [39]. Strains were grown at 37°C with 5% CO_2_, on chocolate agar or brain heart infusion (BHI) supplemented with 10 μg/ml hemin and 10 μg/ml β-nicotinamide (sBHI). Antibiotics were added as required: novobiocin (Nov) at 2.5 μg/ml, nalidixic acid (Nal) at 3 μg/ml, spectinomycin (Spc) at 25 μg/ml, streptomycin (Str) at 100 μg/ml, chloramphenicol (Cm) at 2 μg/ml, and erythromycin (Erm) at 11 μg/ml. *Escherichia coli* strains were grown at 37°C on Luria Bertani (LB), and Cm at 30 μg/ml was added as required.

#### Donor and recipient strains

Donor genomic DNA was obtained from P351, a derivative of the non-typeable otitis media isolate 86-028NP that also carries Nov^R^ and Nal^R^ alleles of *gyrB* and *gyrA* from the multi-antibiotic resistant strain MAP7 [10,28,39] (hereafter NpNN). Two recipient strains were used for natural transformation by this donor DNA: P532, a Spc^R^ derivative of the laboratory strain Rd KW20 [27] (hereafter RdS), and P531, a Str^R^ derivative of the non-typeable otitis media isolate Hi375 [29,86] (hereafter HiT). RdS was produced by transformation of Rd with MAP7 genomic DNA, selection for Spc^R^, followed by screening against other MAP7 resistance alleles. HiT was produced by transformation of Hi375 with a PCR amplicon spanning the Str^R^ allele of the *rpsL* gene from an Rd KW20 Str^R^ strain (P193) produced with “RdS” primers 1497+1798, and selection of transformants on sBHI-agar containing Str 100 μg/ml.

#### Cloning of the HMW1 flanking interval

To test whether the region flanking *hmw1*_*86-028NP*_ played a role in invasion, an interval encompassing *NTHI1981* (*kpsF*) and *NTHI1982* (*yrbI*), and 675 bp upstream, was PCR amplified using 86-028NP genomic DNA as template and “cloning” primers 1219 and 1218. Primer 1218 included an HA tag to add at the 3´end of the *yrbI* gene. This 2,241bp blunt PCR product was phosphorylated with T4 kinase, and cloned into pSU20 [87] pre-digested with *Hinc*II and dephosphorylated with Antarctic phosphatase, generating pSU20-*kpsF-yrbI*-HA. pSU20 and pSU20-*Pr*::*kpsF-yrbI*-HA were transformed into electrocompetent Rd. Tranformants were selected by plating to sBHI-agar containing Cm.

#### HMW adhesin knockouts

Deletion mutations of *hmw* adhesins were generated using one of two approaches. In the first recombineering approach, the first 1kb of the *hmw1A*_*86-028NP*_ gene (7,000bp) was replaced with a Spc^R^ cassette. This was used to generate an *hmw1A*_*86-028NP*_ knockout in the rHiT recombinant clone (P551). Briefly, a ~3kb interval was amplified from NpNN using “interval” primers 13CAN+1273, which spanned the 1kb region targeted for deletion ±1kb, and the purified amplicon was cloned into the pGEMT-easy vector. Separately, a Spc^R^ selectable marker was amplified from pRSM2832 [88] using primers carrying 50 bp overhangs flanking the deletion target (“Deletion” primers 1199 and 1274). The plasmid and amplicon were co-electroporated into DY380/SW102, which carries a heat-shock inducible λ Red recombinase. After recombinase induction, recovery, and selection for Amp^R^ Spc^R^ resistant *E*. *coli* colonies [88], disruption cassettes were confirmed by PCR. Finally, the “interval” primers (13CAN and 1273) were used to amplify the complete disruption cassette from the plasmid prior to natural transformation into rHiT and selection on Spc^R^. Mutants were selected on sBHI-agar containing Spc, and correct targeting was confirmed by PCR and western blot. This procedure generated strain P834.

In the second approach, PCR was used to amplify two ~1kb intervals flanking the region targeted for deletion (Flanks A and B), and an erythromycin resistance cassette (Erm^R^) was added between them. This was used to generate knockouts in HiT (P531), rHiT (P551), and rHiT Δ*hmw1A*_86-028NP_ (P834) in two genes: *hmw1A*_*Hi375*_ (Flank A primers 1463 and 1464; Flank B primers 1465 and 1466) and *hmw2A*_*Hi375*_ (Flank A primers 1467 and 1468; Flank B primers 1469 and 1470). Flanks A and B were amplified with *Sma*I sites included in the R primer of Flank A and the F primer of Flank B, digested with *Sma*I, and blunt-end cloned into pJET1.2 by tri-molecular ligation. The Erm^R^ cassette was excised from pBSLerm [89] using *Sma*I (1,188 bp fragment) and added to the pJET1.2 clone by blunt-ended ligation into the *Sma*I site joining Flanks A and B. Finally, the whole disruption cassette was amplified and transformed into MIV-competent cells of the appropriate strain using the standard protocol [39]. Mutants were selected on sBHI-agar containing Erm, and correct targeting was confirmed by PCR and western blot. This procedure generated strains P836-P839.

### TREP design

The spectrum and distribution of recombinants in transformed pools that carry invasion alleles/loci depends upon the number of loci involved, their genetic interactions, the rates of recombination at those loci, and the experimental environment. To maximize the chance of enriching invasive recombinants from transformed pools: (a) We selected for a donor-specific marker (either Nov^R^ or Nal^R^) after transformations to ensure that recombinant clones were not derived from non-competent cells in the original culture [8,10]. (b) We selected for a recipient-specific marker (Spc^R^ or Str^R^) to limit cross-contamination. (c) Colonies were pooled, so that each independent recombinant in the pools was represented by many (>10^6^) cells. (d) We maximized the complexity of the recombinant pools emerging from the first round of selection. (e) We progressively increased the frequency of invasive recombinants by serial selection, using a pool of the total CFU output from the gentamicin protection assay as the infecting material for the next cycle of selection.

### Natural transformations

Recipient strains were made naturally competent using the standard protocol [39], except scaled up to 10 ml (~10^10^ CFU). Briefly, exponentially dividing cells growing in rich medium (sBHI) were transferred to starvation medium (MIV) for 100 min. Purified genomic DNA from the donor was incubated with naturally competent cultures at a concentration of ~1 genome per cell, or ~2 μg / 10^9^ CFU / ml, for 30 min on a roller drum at 37°C, followed by a 1:5 dilution into sBHI and further incubation for 80 min to allow for expression of resistance alleles. Cultures were diluted and plated on sBHI-agar ± antibiotics to measure transformation and co-transformation frequencies (as Nov^R^ or Nal^R^ resistant colonies / CFU). Percent competence was calculated as (Nov^R^ Nal^R^ / CFU) / (Nov^R^/CFU * Nal^R^/CFU), as previously described [8,10,90]. To generate high complexity pools of recombinants, we plated 0.75 ml of a 10^−2^ dilution to 20 large petri dishes (20 cm diameter): 10 containing Nov and 10 with Nal (plus Spc or Str, depending on the recipient). This yielded ~10^4^ resistant colonies per plate. Colonies from each set of 10 plates were scraped into a single 10 ml sBHI pool, titrated by dilution and plating to sBHI+antibiotics, and immediately stored as 1.25 ml aliquots in 15% glycerol at -80°C. This generated a total of four pools with an initial complexity of ~10^5^ independent recombinants each, two for each recipient (Rd KW20 Spc^R^ and Hi375 Str^R^), selected for either the Nov^R^ or the Nal^R^ donor allele, as well as a second antibiotic to select for the appropriate recipient background.

### Infection of cultured epithelial cells and measurements of adhesion and intracellular invasion frequencies

The carcinomic human alveolar basal epithelial cell line A549 (ATCC CCL-185) was maintained in RPMI 1640 medium supplemented with 10 mM Hepes, 10% heat-inactivated fetal calf serum (FCS) and antibiotics (penicillin 100 units/ml and Str 0.1 mg/ml) in 25 cm^2^ tissue culture flasks at 37°C in a humidified 5% CO_2_ atmosphere. Chang cells (ATCC CCL-13) were cultured under the same atmospheric conditions in Minimum Essential Medium Eagle supplemented with 10% FCS and 1x MEM non-essential amino acid mixture (Sigma). Cells were seeded to 6×10^4^ or to 1.2×10^5^ cells / well in 24- or in 6-well tissue culture plates, respectively, for 32 h, and then serum starved for 16 h before infection. A ~90% confluence was reached by the time of infection. Adhesion and intracellular invasion assays in 24-well plates were conducted as previously described [15,24], starting with *H*. *influenzae* cells scraped from chocolate-agar plates (freshly grown for 16 h at 37°C with 5% CO_2_) into PBS and adjusted to OD_600_ = 1. A small aliquot of this adjusted suspension was diluted and plated on sBHI-agar to titrate the input CFU.

For invasion assays, A549 cells were incubated with 0.2 ml of each adjusted bacterial suspension for 2 h, washed 3 times with PBS and incubated for 1 h with RPMI 1640 containing 10% FCS, Hepes 10 mM and gentamicin 200 μg/ml to kill extracellular bacteria (the bacterial isolates used all had minimum inhibitory concentrations of < 5 μg/mL), washed 3 times with PBS, and human cells were lysed with 300 μl of PBS-saponin 0.025% for 10 min at room temperature. To quantify intracellular invasion frequencies, lysates were serial diluted and plated onto sBHI-agar with appropriate antibiotics. Recovered CFU was divided by the input CFU to calculate “Invaders/CFU”. Unless otherwise indicated, all infections were carried out in triplicate on three separate occasions. Adhesion assays were carried out similarly, excluding gentamicin treatment to calculate “Adherents/CFU”. For adhesion assays, cells were incubated with 0.1 ml of each adjusted bacterial suspension for 30 min. Wells were then washed 5 times with PBS and lysed as above. An alternative method was also used for both invasion and adhesion for comparisons of Rd, Strain 12, and Rd/HMW1_Strain12_ following a previously published protocol [18]. The primary differences were the lack of a serum starvation step, a low-speed centrifugation step to quickly bring bacteria into contact with the monolayer, and a lower MOI.

### Serial enrichment for invasive recombinants

To enrich for recombinants carrying donor-specific invasion alleles, we performed eight serial selections for invasive clones for each recombinant pool. To maximize the complexity of the initial recombinant pools (Pool 0), one frozen aliquot per pool (~10^10^ CFU of ~10^5^ independent recombinants per aliquot) was used to infect three wells of A549 cells seeded onto 6-well plates. Pool 0 aliquots were first recovered by thawing, pelleting, resuspending in 5 ml sBHI, and incubating on a roller drum for 60 min at 37°C under 5% CO_2_. Cultures were pelleted prior to proceeding with the invasion protocol, performed as described above, scaled up to cells seeded on 6-well plates (in 4 ml EBSS, with 0.8 ml of bacterial adjusted suspension / well), starting with resuspension of pellets in PBS, and ending with the total lysate plated on sBHI-agar (+appropriate antibiotics) at varying dilutions. This allowed measurement of intracellular invasion frequency and provided material for the next cycle.

For each subsequent serial invasion cycle, all CFU were scraped off plates into PBS and thoroughly mixed before normalizing to OD_600_ = 1 and proceeding with the infection. For all cycles, unused material was stored as (i) 15% glycerol stocks at -80°C for repeat assays and isolation of individual clones, and (ii) as a pellet at -20°C for DNA extractions (except for the RdS Pool 1 material, for which none was left over). In practical terms, this serial infection procedure was repeated for four enrichment cycles, at which point recovered pools were frozen as 15% glycerol stocks to allow for a new set of confluent A549 cells to grow up; frozen stocks were then restarted to carry out four additional cycles of selection. Untransformed recipient controls were run in parallel to exclude potential issues related to cell seeding. At Pool 4, several single gentamicin-protected clones per enrichment were isolated on sBHI-agar plates and stored at -80°C in 15% glycerol for adhesion and invasion assays, as well as clone sequencing.

### Western blot

To monitor YrbI-HA expression, whole cell extracts from strain Rd alone, Rd carrying pSU20, and Rd carrying pSU20-*Pr*::*kpsF-yrbi*-HA were prepared from bacterial cultures grown to OD_600_ = 0.9 in sBHI containing Cm, when required. YrbI-HA expression was analyzed by western blot with a primary rabbit anti-HA antibody (Sigma) diluted 1:4000, and a secondary goat anti-rabbit IgG (whole molecule, Sigma) antibody conjugated to horseradish peroxidase, diluted 1:1000.

To investigate HMW adhesin protein expression in strains NpNN, RdS, rRdS, HiT, HiTΔ*hmw1A*_*Hi375*_, HiTΔ*hmw2A*_*Hi375*_, rHiT, rHiTΔ*hmw1A*_86-028NP_, rHiTΔ*hmw1A*_Hi375_, rHiTΔ*hmw1A*_86-028NP_Δ*hmw1A*_*Hi375*_, whole cell extracts were prepared from bacterial suspensions recovered from overnight grown chocolate-agar plates and adjusted to OD_600_ = 1 in PBS. HMW1A expression was analyzed by western blot with a primary guinea pig anti-HMW1A (gp85) antibody diluted 1:2000 [91], and a secondary goat anti-guinea pig IgG (Santa Cruz) antibody conjugated to horseradish peroxidase, diluted 1:5000.

### Bacterial self-aggregation

*H*. *influenzae* cells were scraped from chocolate-agar plates freshly grown for 16 h at 37°C with 5% CO_2_ into PBS solution, and adjusted to OD_600_ = 0.45 in a 35 ml volume, and left standing at room temperature for at least 260 min. OD_600_ readings were performed at regular time intervals on 500 μl aliquots gently collected from the top of each bacterial suspension. Four independent experiments were performed for each strain.

### Immunofluorescence microscopy

A549 cells were seeded on 13 mm circular coverslips in 24-well tissue culture plates. Cells were infected at an MOI ~1:8 (5 μl) of each adjusted bacterial suspension for 2 h, and infected cells were incubated in RPMI 1640 containing 10% FCS, Hepes 10mM and gentamicin 200 μg/ml for 1 h. Cells were washed three times with PBS and fixed with 3.7% paraformaldehyde (PFA) in PBS pH 7.4 for 15 min at room temperature. Immunofluorescence staining was carried out as previously described [24]. *H*. *influenzae* cells were stained with a rabbit anti-NTHi serum (raised against a pool of strains Hi375, 2019, and 398 [24]) diluted 1:600. Late endosomes were stained with mouse monoclonal anti-human Lamp-1 H4A3 antibody (Developmental Studies Hybridoma Bank) diluted 1:100. DNA was stained with Hoechst 33342 (Invitrogen) diluted 1:2500. Donkey anti-rabbit conjugated to Cy2 and donkey anti-goat or donkey anti-mouse conjugated to Rhodamine secondary antibodies (Jackson) were diluted 1:100.

Samples were analyzed with a Carl Zeiss Axioskop 2 plus fluorescence microscope and a Carl Zeiss Axio Cam MRm monochrome camera. We quantified: (a) the percentage of infected cells, counting at least 250 cells per sample; (b) the number of bacteria per infected cell in at least 250 cells per sample type, scoring <10 bacteria/cell or >10 bacteria/cell; (c) co-localization of bacteria and Lamp-1—an NTHi-containing vacuole (NTHi-CV) was considered positive for Lamp-1 when the marker was detected throughout the area occupied by the bacterium, or around/enclosing the bacterium. To determine the percentage of bacteria that co-localized with Lamp-1, all bacteria located inside a minimum of 150 infected cells were scored in each experiment. Results were calculated from two independent experiments.

### DNA sequencing

Genomic DNA was extracted from the donor and recipients, stored pools, and isolated clones by phenol/chloroform extraction as described [8]. Purity and quality were evaluated by Nanodrop spectrophotometry (Thermo Scientific) and agarose gel electrophoresis, and quantification was performed with Qbit fluorometry prior to sequencing library construction. Multiplexed sequencing libraries were produced using the Nextera XT kit following manufacturer recommendations (Illumina). Paired-end sequencing (2x101nt) was conducted on an Illumina HiSeq in RapidRun mode over several independent runs/lanes. Raw base call data (bcl) was converted into FastQ format (Illumina version 1.8) using the bcl2fastq conversion software from Illumina (version 1.8.3, setting—no-eamss). For recombinant clones, paired-end sequencing (2x151nt) was conducted on an Illumina MiSeq, which automates demultiplexing to provide raw FastQ files. Properties of the genomic DNA samples and sequencing statistics (including donor and recipient controls) are in S2 and S3 Tables.

### Read alignments and variant calling

The genome sequence references for the donor and recipients were: 86-028NP (NC_007146.2) [28], Rd_KW20 (NC_000907.1) [27], and Hi375 (CP009610.1) [29]. For the Rd genome, all non-ACGT bases were first converted to Ns (some non-N ambiguous IUPAC nucleotide characters lead to errors running samtools mpileup). Reads from control strains were used to identify variation between the derivative strains’ genomes and their deposited parental reference sequences (as described below). For all raw Illumina sequence processing, paired-end reads were trimmed of adapter sequences with Trimmomatic (v0.32) [92] and overlapping pairs were merged with COPE (v1.1.3; simple-connect mode) [93]. Next, reads were mapped using bwa mem (v0.7.8) with default settings [94], duplicates were marked with SamBlaster (v0.1.14) [95], and aligned reads were sorted and compressed using SamBamba (v0.4.6) [96]. Subsequent steps filtered out reads with a mapping quality = 0, which excludes multiply mapping reads that align equally well to different reference genome coordinates.

For donor and recipient controls, as well as recombinant clones, single-nucleotide polymorphism (SNP) and small indel variant calling used samtools mpileup and bcftools view (v0.1.19) [97]. Variant frequency calling from recombinant pools used a python script (available at https://github.com/photonchang/allelecount/) to count reads supporting each of the 4 bases at each reference position directly from samtools mpileup output (for base calls with quality score >10), and subsequent parsing used linux commands (mostly awk). BedTools (v2.19.1) [98] was used for subsetting (using the intersect tool) with the variants detected between donor and recipient genomes. Variant tables were first corrected for “self” variants identified between reads and their own reference (with the exception of resistance-associated markers). This allowed calculation of recipient-specific, donor-specific, and erroneous base frequencies (*i*.*e*. bases with neither donor nor recipient identity). Manual validation of recombination breakpoints and clone assignments used the Integrative Genomics Viewer (v2.3.1) [99]. Identification of novel alleles that had approached fixation compared the variants called from Pool 8 reads to those from Pool 0 reads (using samtools mpileup and bcftools view). Due to systematic alignment artifacts that arise when mapping donor reads to recipient genomes in regions of high divergence, putative novel variation that was also identified only in reciprocal alignments of control reads (“unreliable” SNP positions) was excluded, leaving no observed fixed new mutations.

#### PCR validation

To distinguish between the four possible *hmw* genotypes, allele- and locus-specific PCR was used, with allele-specific primer pairs listed under “Allele ID” in S10 Table. Each pair is specific for one of the four possibilities and generates a distinct PCR product size as determined by standard agarose gel electrophoresis. Primers 1456+1458 were used for *hmw1A*_*Hi375*_ (product size 1,364 bp), primers 1456+1457 for *hmw2A*_*Hi375*_ (product size 1,076 bp); primers 1459+1460 for *hmw1A*_*86-028NP*_ (product size 744 bp); and primers 1461+1462 for *hmw2A*_*86-028NP*_ (product size 582 bp).

### Statistics and plotting

Significant differences in invasion, adhesion, and self-aggregation phenotypes among strains and pools were evaluated using one-way ANOVA with *post hoc* hypothesis testing using Tukey’s HSD (“honest significant differences”). Invasion and adhesion frequencies were first log-transformed prior to testing to account for the highly unequal variances observed between strains/pools that were quantified at distinct plating dilutions. Pairwise student’s t-tests with untransformed data and Bonferroni correction gave qualitatively similar results. Plotting used the R statistical programming language including add-on packages seqinr, genoplotr, ggplot2, and Rcolorbrewer.

### Data deposition

All sequence data were deposited at NCBI under BioProject PRJNA308311. BioSample accessions are included in S2 and S3 Tables. Parental strains were submitted to the SRA as BAM files aligned to their own reference sequence. Recombinant pool and clone data were submitted to the SRA as BAM files aligned to the appropriate recipient reference sequence (Hi375 or Rd KW20).

## Supporting Information

S1 Text Supplementary Results and Supplementary References. (DOCX)

S1 Fig Comparison of the donor to the two recipients at different scales. Turquoise lines above the x-axis indicate the position of SNPs distinguishing donor from recipient, while grey lines below the x-axis indicate positions in the recipient genome missing from the donor genome (at indels). **(A)** Hi375 recipient. **(B)** Rd KW20 recipient. Note that SNPs between Hi375 and 86-028NP are punctate, with stretches of very low SNP density punctuated by stretches of high SNP density. Genomic positions exclusive to the recipient strains are shown in grey; these coincide with areas that appear as regions of low SNP density, but these artifacts are insufficient to explain the pattern seen in Hi375. Conversely Rd-specific positions do largely explain low SNP density regions in Rd KW20.
(TIF)

S2 Fig Invasion and adhesion phenotypes of parental and related strains. **(A)** Invasion of and **(B)** adhesion to A549 cells is shown for *H*. *influenzae* strains Rd KW20, Hi375, 86-028NP, and antibiotic resistant derivatives, including the parental strains.
(TIF)

S3 Fig Competition for invasion between Rd and 86-028NP strain backgrounds. Two serial cycles of selection for intracellular invaders were conducted using three mixtures of 86-028NP Nov^R^ and Rd Str^R^ cells, at 1:100, 1:1,000, or 1:10,000 ratios. Prior to the first infection (input.A), the bacterial cell suspension was titrated for the total Nov^R^ and Str^R^ CFU used per well, and this closely matched the expected frequencies. After the first round of selection (output.A), dramatically fewer CFU were recovered, but Nov^R^ were proportionally much more abundant. Total unselected CFUs were pooled and titrated (input.B), showing that the proportion of Nov^R^ remained relatively the same in between cycles of selection for invasion. Finally, the second cycle of selection resulted in a higher yield with an even higher proportion of Nov^R^ colonies, representing a strong enrichment of 86-028NP over Rd, even when at a low relative abundance in the starting mixture.
(TIF)

S4 Fig Donor allele frequencies in the transformed input pools. **(A)** and **(B)** NpNN-specific SNP frequencies as a function of chromosome coordinate for the RdS and HiT recipients, respectively, at Pool 0, prior to enrichment for invasive recombinants. Left panels: Nov^R^-selected pools. Right panels: Nal^R^-selected pools. Top panels: chromosome-wide view. Bottom panels: zoom on 60 kb windows around the antibiotic resistance markers. The peak SNP is the one conferring antibiotic resistance. **(C)** “Bean plots” summarizing 16 histograms of non-recipient allele frequencies for untransformed controls and the initial transformed recombinant pools. The left side (salmon-colored) of each bean shows a histogram for allele frequencies with donor allele identities, whereas the right side (light blue) shows a histogram for “novel” alleles (neither recipient nor donor). The latter are sequencing errors, while the former are sequencing errors for the control strains and a combination of sequencing errors and transformants for the transformed pools.
(TIF)

S5 Fig Serial selection of invasive recombinants by gentamicin protection. Invaders/CFU for pools during the initial eight serial selections for invasive recombinants. Recovered CFU that survived gentamicin treatment (Pool 1) served as input for the next cycle (which generated Pool 2). The values show the combined ability of clones in Pool *n* to invade airway epithelial cells, while the recovered colonies comprise Pool *n+*1. This procedure was carried out eight times. The apparent decline in invasiveness seen at Pool 4 appears to be an artifact, since no such decline was seen in the replicate assays (Fig 3A). Instead, this drop likely reflects that Pool 4 bacteria had been frozen and re-inoculated prior to the next cycle, combined with batch-to-batch variation of the confluent A549 cells used.
(TIF)

S6 Fig No improvement by selection on untransformed recipients. Control experiment using untransformed recipients cultures in triplicate found no increase in invasiveness over 5 serial selections. This experiment was conducted independently for each of the recipients and separately from the experimental enrichments to minimize enrichment of cross-contaminants.
(TIF)

S7 Fig Complexity of recombination tracts decreases at the antibiotic resistance markers over serial passages. Genomic profiling at antibiotic-selected sites for both the **(A)** RdS and **(B)** HiT recipients at Nov^R^ (top) and Nal^R^ (bottom) sites (*gyrB* and *gyrA* respectively, see S6 Table) for Pools 0, 2, 4, and 8. Axes are as in other figures with x-axes indicating recipient genome coordinate (in kb) and the y-axis indicating donor allele frequency. RdS Nov^R^ contains a single clone at ~95% by Pool 8, while RdS Nal^R^ contains two dominant clones, one at ~70% and the other ~30%. HiT Nov^R^ contains two dominant clones (at ~30% and 70%), whereas HiT Nal^R^ appears to contain two clones at ~80% and ~20%. For this pool, only a single genotype (the one at ~80%) was recovered in the four individual clones collected from Pool 4. No other donor segments appeared at ~20%, so this is likely due to incomplete fixation of the invasive genotype after several rounds of selection.
(TIF)

S8 Fig Read alignment artifact at the *radA*-proximal *hmw1* locus in the HiT pools. **(A)** Pools 0, 2, 4, and 8 for HiT Nov^R^ and HiT Nal^R^ as in other figures (x-axis is HiT recipient coordinate in kb, and y-axis is donor allele frequency). **(B)** Genomic map around the same interval. The thick black horizontal line shows the entire range of positions containing donor frequencies >5%. The affected interval spans only the hmw1 locus; no flanking variation was detected, unlike the situation at the *yrbI*-adjacent *hmw2*_*Hi375*_, which was replaced by the *hmw1*_*86-028NP*_ allele. Donor allele frequencies are highly variable in this region. They are also highly consistent between the two pools, which was unexpected, as all other overlapping donor segments detected had distinct recombination breakpoints. Allele-specific PCR assays confirm this as read alignment artifact and confirm that the *radA*-proximal adhesin remain *hmw1*_*Hi375*_ across strains (S9 Fig).
(TIF)

S9 Fig Agarose gel showing allele/locus-specific PCR products amplified for the four possible *hmw* alleles. Strains are listed as it follows: (1) NpNN, (2) RdS, (3) rRdS, (4) HiT, (5) HiTΔ*hmw1A*_*Hi375*_, (6) HiTΔ*hmw2A*_*Hi375*_, (7) rHiT, (8) rHiTΔ*hmw1A*_*86-028NP*_, (9) rHiTΔ*hmw1A*_*Hi375*_, (10) rHiTΔ*hmw1A*_*86-028NP*_Δ*hmw1A*_*Hi375*_, and primers are in S10 Table. **(A)** Primers 1456+1458 identify *hmw1A*_*Hi375*_ (1,364 bp product); **(B)** primers 1456+1457 identify *hmw2A*_*Hi375*_ (1,076 bp product); **(C)** primers 1459+1460 identify *hmw1A*_*86-028NP*_ (744 bp product); and **(D)** primers 1461+1462 for *hmw2A*_*86-028NP*_ (582 bp product). Lanes 8 and 10 rendered a correct size band upon PCR with primers 1459+1460 because mutant strains lacking *hmw1A*_*86-028NP*_ were generated by partial deletion that maintains the annealing sites for the primers and product size.
(TIF)

S10 Fig Confirmation of HMW adhesion expression and testing for a role by *kpsF* and *yrbI*. **(A)** Western blot showing expression of HMW1A_86-028NP_ (154 KDa) adhesin. Whole cell extracts of NpNN, RdS and rRdS (P540, genotype B) were prepared and used to detect HMW by immunoblot with the guinea pig anti-HMW1A gp85 antibody. **(B and C)** Addition of the *kpsF* and *yrbI* alleles from 86-028NP on a plasmid does not increase intracellular invasion frequencies. **(B)** Western blot showing expression from plasmid carrying an interval carrying *kpsF-yrbI* from 86-028NP. Whole cell extracts of cultures (Rd, Rd pSU20, and Rd pSU20-*kpsF-yrbI*-HA) were prepared and used to detect Hap-HA by immunoblot with a rabbit anti-HA antibody, finding expression of the expected ~19.3-kDa protein in the expected strain. **(C)** The same strains were used to infect A549 cells and measure bacterial intracellular invasion. Experiments were performed three times in triplicate (different symbols denote independent experiments).
(TIF)

S11 Fig A role for HMW1 is seen for a distinct strain, for another epithelial cell type, and with an alternative protocol. Invasion **(A)** and adhesion **(B)** by Rd, Rd *hmw1*_*strain12*_, and Strain 12 bacterial strains into Chang and A549 epithelial cell lines. An alternative protocol that includes centrifugation to quickly bring bacteria into contact with the cell monolayer was used for these experiments, showing that both cell type and details of the infection procedure give qualitatively similar results.
(TIF)

S1 Table Strains and Plasmids Used (DOCX)

S2 Table Pool and control sequencing statistics (DOCX)

S3 Table Recombinant clone sequencing statistics (DOCX)

S4 Table Alignment statistics for the untransformed controls (DOCX)

S5 Table Transformation frequencies and estimated competence (DOCX)

S6 Table Allele frequencies around antibiotic resistances in Pool 0 (DOCX)

S7 Table Non-reference alleles at reliable SNP positions in transformed pools and untransformed controls (DOCX)

S8 Table Clone genotype assignments (DOCX)

S9 Table Donor segments detected in each isolated genotype (DOCX)

S10 Table Primers used in this study (DOCX)
