# Correction: Transformed Recombinant Enrichment Profiling Rapidly Identifies HMW1 as an Intracellular Invasion Locus in *Haemophilus influenzae*

**DOI:** 10.1371/journal.ppat.1005681

**Published:** 2016-05-25

**Authors:** The PLOS Pathogens Staff

## Notice of Republication

This article was republished on May 13, 2016, to correct the title which was incorrectly published as “Transformed Recombinant Enrichment Profiling Rapidly Identifies HMW1 as an Intracellular Invasion Locus in *Haemophilus influenza”*. The publisher apologizes for the errors. Please download this article again to view the correct version. The originally published, uncorrected article and the republished, corrected articles are provided here for reference.

## Supporting Information

S1 File Originally published, uncorrected article. (PDF)

S2 File Republished, corrected article. (PDF)
