# Supplementary material for: Transformed Recombinant Enrichment Profiling Rapidly Identifies HMW1 as an Intracellular Invasion Locus in Haemophilus influenzae
Source: PLoS Pathog. 2016 Apr 28;12(4):e1005576. doi: 10.1371/journal.ppat.1005576 (PMC4849778; doi:10.1371/journal.ppat.1005576)
Supplement: S1 Text — (DOCX) [file ppat.1005576.s001.docx]

**S1 Text: Supplementary Results and References**

“Transformed recombinant enrichment profiling rapidly identifies HMW1 as an intracellular invasion locus in *Haemophilus influenzae*” by Mell *et al.*

**Supplementary Results**

**Genomic comparisons of donor to recipients**. The three parental strains can be distinguished by tens of thousands of single-nucleotide polymorphisms distinguishable by genomic DNA sequencing, as well as by hundreds of “accessory” or “distributed” loci present in only some strains. Re-sequencing the parental strains was used as a control to identify unambiguous and reliable genetic markers, as well as to identify genomic positions with systematically low coverage or ambiguous base calls. Sequence reads collected from the recipients were aligned to their respective references, also to the donor, and *vice versa*.

To evaluate the quality of the DNA sequencing and read alignment and to correct the references for intra-strain variation, we examined variant frequencies at each position after mapping reads from the parents to their own genomic reference (S4 Table). Variants could be any of the 3 non-reference bases aligned to each reference genome position. Nearly all positions had 10 or more reads aligned to them (>99.9% of positions for all five controls), with mean depths per control averaging 372 reads per position. For all controls, >50% of positions showed no variation whatsoever from the reference base, with a mean frequency for non-reference variants (VarFreq) of ~10^-3^ per position or less (median VarFeq=0).

A small number of positions showed evidence for high frequency strand-specific sequencing errors (as determined by Fisher’s exact test with a p-value cut-off of 0.05), and these had a disproportionately high VarFreq (S4 Table). When only positions with no evidence for strand bias were considered, <2.5% of had a VarFreq > 0.01. Together, these data indicate that sequencing and alignment artifacts are rare, with most positions having no evidence of non-reference bases, even at very high sequencing depths. These data also suggest that, as long as donor allele frequencies exceed a few percent, sequencing-based measurements are unaffected by errors at nearly every genomic position.

Control sequence alignments also identified positions that differed between the reference sequences and the parental strain derivatives used for the experiments (last row, S4 Table). Variants in HiT are due to the addition of the Str^R^ marker, which also carried several Rd-specific SNPs, and similarly variants in NPNN are due to addition of the Nov^R^ and Nal^R^ markers, which added adjacent Rd-specific SNPs [2,3]. Differences between the Rd-derivatives and their reference are largely due to errors in the Rd KW20 genome sequence produced in 1995, as previously described [2], but also include MAP7-specific genetic variation, including seven antibiotic resistance markers (as previously described in [3]), in the case of the RdS strain, only the Spc^R^ marker. Identifying these variants between the strains and their references allowed for correction of reference base at these positions in the pool and clone-based genotyping used for TREP mapping.

To identify unambiguous and reliable genetic markers, reciprocal alignments between donor and recipient reads to donor and recipient genomes was used to identify mutually consistent variant calls. This set of reliable SNP positions required that alignment of donor and recipient reads to their own reference supported the reference base and a variant base when aligned to their reciprocal reference. This procedure also eliminated putative SNP positions that, when aligned to either reference, generated mixed (or “heterozygous”) genotype calls, including high-frequency strand-biased positions. Whole-genome alignnments generated by Mauve provided a coordinate liftover table for cross-referencing positions between donor and recipient genomes. Comparisons of 86-028NP and Rd have previously been made [[1](#_ENREF_1),[2](#_ENREF_2)]; here we found 36,869 reliable SNPs. Comparison of 86-028NP and Hi375 found this pair of strains to be more similar, reliably distinguished by 19,520 SNPs. Notably, the genetic variation between 86-028NP and Hi375 was punctuated by segments of much lower sequence identity (S2 Fig.). This mosaic divergence seemed unusual, suggesting that these strains may have once had a history of co-occurrence and genetic exchange, despite the isolation of the strains from different parts of the world (86-028NP in USA and Hi375 in Finland).

**Isolation of the antibiotic resistance markers to nucleotide resolution.** The frequency of donor-specific SNPs along the genome in the transformed recombinant pools (Pool 0) identified the known variants responsible for antibiotic resistance after a single round of strong selection (S4 Fig. parts A and B). All four pools started with nearly 100% donor-specific Nov^R^ or Nal^R^ alleles. Three recombinant pools were as expected (nearly 100% Nov^R^ or Nal^R^ donor-specific alleles), but a manipulation mistake during pooling from independent plates led to the RdS Nal^R^ pool being ~20% Nov^R^ (S4 Fig. part A, right panels); this did not interfere with mapping invasion loci/alleles. “Novel” alleles (base calls with neither donor nor recipient identity) occurred at a frequency consistent with overall error rates (S4 Table and S5 Table, and see below).

Because the donor and recipient genomes are divergent, clone sequencing could have identified recombinant segments spanning the selected site but not have isolated the exact resistance alleles [[3](#_ENREF_3)]. Here, the high complexity of the recombinant pools after selection (~10^5^ colonies) allowed for identification of the exact causative nucleotide, since donor alleles immediately adjacent to the resistance alleles had lower frequencies (S6 Table). The smooth decline in donor SNP frequencies on either side of the antibiotic-selected sites further underscores the pools’ high complexities (S4 Fig. parts A and B), since less complex pools have a more saw-toothed or step-wise pattern [[3](#_ENREF_3)]. Consistent with previous observations using a small set of sequenced recombinant clones [[3](#_ENREF_3)], the decay in donor allele frequencies was asymmetric and non-uniform, suggesting variation in transformation frequencies for positions flanking the selected sites, at least in part due to the presence of nearby structural variation.

These results validate TREP, showing that causative single nucleotide differences can be identified directly from selected pools. In this situation, the transformation frequency of the causative alleles was high, the selection was very strong, and the alleles had very large effect sizes. For other phenotypes, like intracellular invasion, serial selections would allow for weaker selections and concomitant enrichment of alleles with weaker effects, though possibly at the expense of reduced complexity in enriched pools, especially if transformation by the relevant alleles is infrequent.

**Genome-wide transformation in the recombinant pools.** Because TREP relies on the enrichment of donor-specific genetic variation through serial selection, these experiments do not need to be specifically designed to measure the low transformation frequencies (<1%) in the initial recombinant pools prior to selection, *per* se, which would require considerably higher sequencing depth, along with other experimental design and bioinformatics analysis considerations not needed for TREP, mostly having to do with transformation frequencies being similar to average Illumina sequencing error rates. Nevertheless, we were able to check the initial recombinant pools (Pool 0s) for evidence that transformation had occurred genome-wide, albeit with a relatively high limit-of-detection and in the context of sequencing errors. Several lines of evidence show that the recombinant pools carried donor-specific genetic variation of all types across the genome prior to serial selections for intracellular invasion and not only near the antibiotic-selected sites: (a) Compared to untransformed controls, non-reference variants with a donor base identity in the transformed pools had non-zero frequencies at substantially more positions (~3-fold or more, S6 Table). (b) “Donor” variant frequencies in the recombinant pools were significantly higher than in untransformed controls, where “donor” variants are just 1 of 3 possible sequencing/alignment errors (S4 Fig. part C, p<<0.01 for one-way ANOVA). (c) In contrast, untransformed controls had putative “donor” variant frequencies similar to or lower than “novel” variant frequencies ( the frequency of the two bases that had neither donor nor recipient identity), as would be expected for sequencing/alignment artifacts (S4 Fig. part C; Tukey’s HSD p>0.1 for comparisons of “donor” frequencies to error rate in controls, but p<<0.001 for transformed pools, compared to “novel” allele frequencies). Finally, (d) donor-specific structural variants (including donor-specific genes) were also incorporated into recombinant chromosomes in pools, indicated by sequence reads failing to align to the recipient reference but aligning to the donor reference >5-fold better than reads from untransformed controls (S7 Table).

Suggestive of potential “hot” and “cold” regions for transformation, donor-specific SNP frequencies in the recombinant pools—but not the control strains—had a markedly bimodal distribution, with the low frequency mode being comparable to the rate of erroneous “donor” variants in untransformed controls (S4 Fig. part C). Unfortunately, these experiments do not accurately capture global transformation frequencies, due to insufficient coverage (limits of detection >0.1%). However, for purposes of TREP, the data demonstrate that the starting recombinant pools are highly complex and likely carry much of the genetic variation distinguishing the donor from the recipient. As long as phenotypic selection increases the frequency of recombinant (or mutant) clones above a few percent, sequencing artifacts will not confound identifying the donor-specific (or novel) genetic variation in selected clones (outside of the <1% of genomic positions with low coverage or ambiguous base calls).

**Supplementary References**

1. Harrison A, Dyer DW, Gillaspy A, Ray WC, Mungur R, et al. (2005) Genomic sequence of an otitis media isolate of nontypeable *Haemophilus influenzae*: comparative study with *H. influenzae* serotype d, strain KW20. J Bacteriol 187: 4627-4636.

2. Mell JC, Shumilina S, Hall IM, Redfield RJ (2011) Transformation of natural genetic variation into *Haemophilus influenzae* genomes. PLoS Pathog 7: e1002151.

3. Mell JC, Lee JY, Firme M, Sinha S, Redfield RJ (2014) Extensive cotransformation of natural variation into chromosomes of naturally competent *Haemophilus influenzae*. G3 (Bethesda) 4: 717-731.

4. Wilcox KW, Smith HO (1975) Isolation and characterization of mutants of *Haemophilus influenzae* deficient in an adenosine 5'-triphosphate-dependent deoxyribonuclease activity. J Bacteriol 122: 443-453.

5. Catlin BW, Bendler JW, 3rd, Goodgal SH (1972) The type b capsulation locus of *Haemophilus influenzae*: map location and size. J Gen Microbiol 70: 411-422.

6. Hood DW, Makepeace K, Deadman ME, Rest RF, Thibault P, et al. (1999) Sialic acid in the lipopolysaccharide of *Haemophilus influenzae*: strain distribution, influence on serum resistance and structural characterization. Mol Microbiol 33: 679-692.

7. Barenkamp SJ, Leininger E (1992) Cloning, expression, and DNA sequence analysis of genes encoding nontypeable *Haemophilus influenzae* high-molecular-weight surface-exposed proteins related to filamentous hemagglutinin of *Bordetella pertussis*. Infect Immun 60: 1302-1313.

8. Grass S, Buscher AZ, Swords WE, Apicella MA, Barenkamp SJ, et al. (2003) The *Haemophilus influenzae* HMW1 adhesin is glycosylated in a process that requires HMW1C and phosphoglucomutase, an enzyme involved in lipooligosaccharide biosynthesis. Mol Microbiol 48: 737-751.

9. Tracy E, Ye F, Baker BD, Munson RS, Jr. (2008) Construction of non-polar mutants in *Haemophilus influenzae* using FLP recombinase technology. BMC Mol Biol 9: 101.

10. Sanchez R (1998) A medium-copy-number plasmid for insertional mutagenesis of *Streptococcus mutans*. Plasmid 40: 247-251.
