# Supplementary figures and images for: Transformed Recombinant Enrichment Profiling Rapidly Identifies HMW1 as an Intracellular Invasion Locus in Haemophilus influenzae
Source: PLoS Pathog. 2016 Apr 28;12(4):e1005576. doi: 10.1371/journal.ppat.1005576 (PMC4849778; doi:10.1371/journal.ppat.1005576)

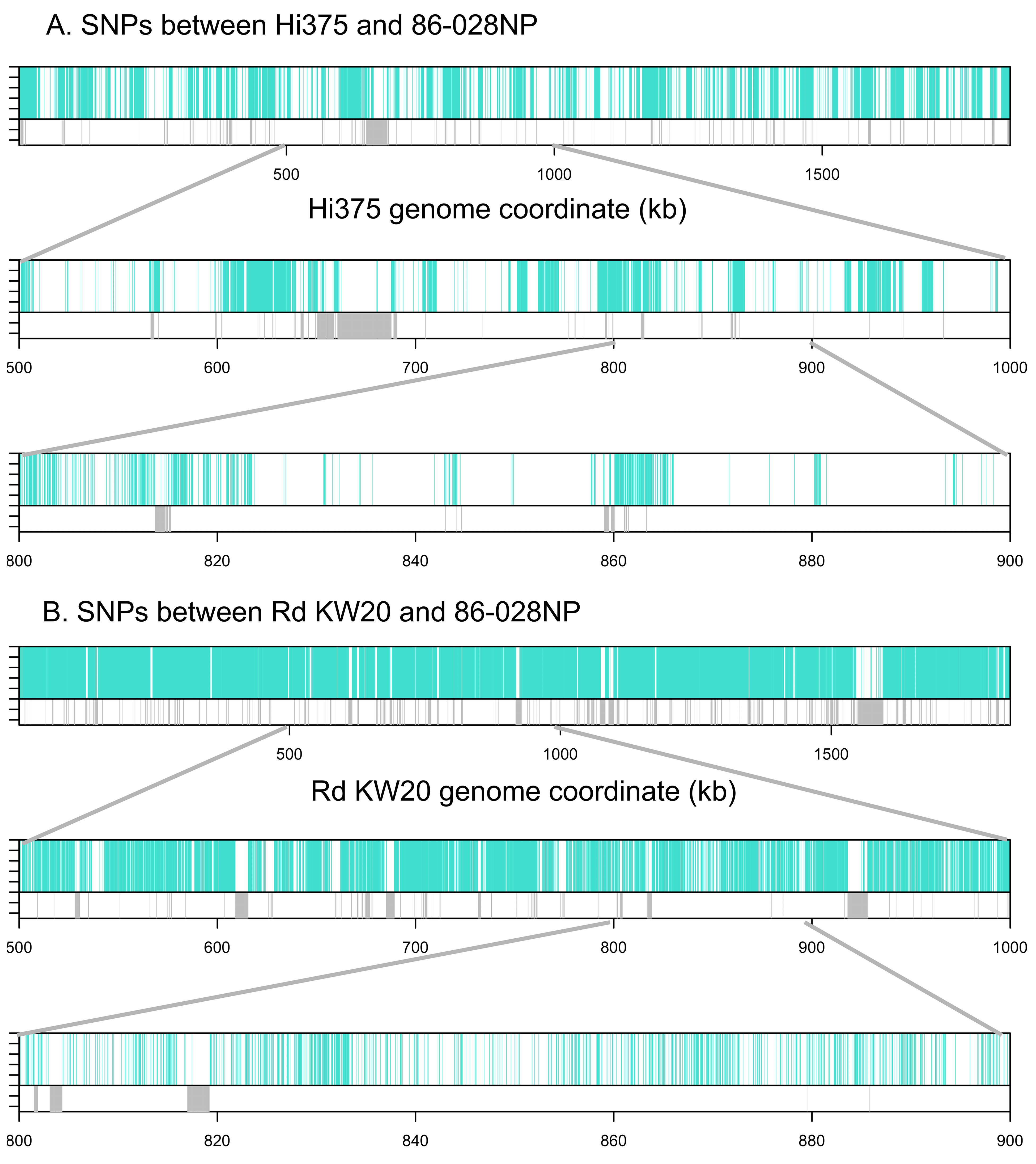

Supplement: S1 Fig — Turquoise lines above the x-axis indicate the position of SNPs distinguishing donor from recipient, while grey lines below the x-axis indicate positions in the recipient genome missing from the donor genome (at indels). (A) Hi375 recipient. (B) Rd KW20 recipient. Note that SNPs between Hi375 and 86-028NP are punctate, with stretches of very low SNP density punctuated by stretches of high SNP density. Genomic positions exclusive to the recipient strains are shown in grey; these coincide with areas that appear as regions of low SNP density, but these artifacts are insufficient to explain the pattern seen in Hi375. Conversely Rd-specific positions do largely explain low SNP density regions in Rd KW20. (TIF) [file ppat.1005576.s002.tif]

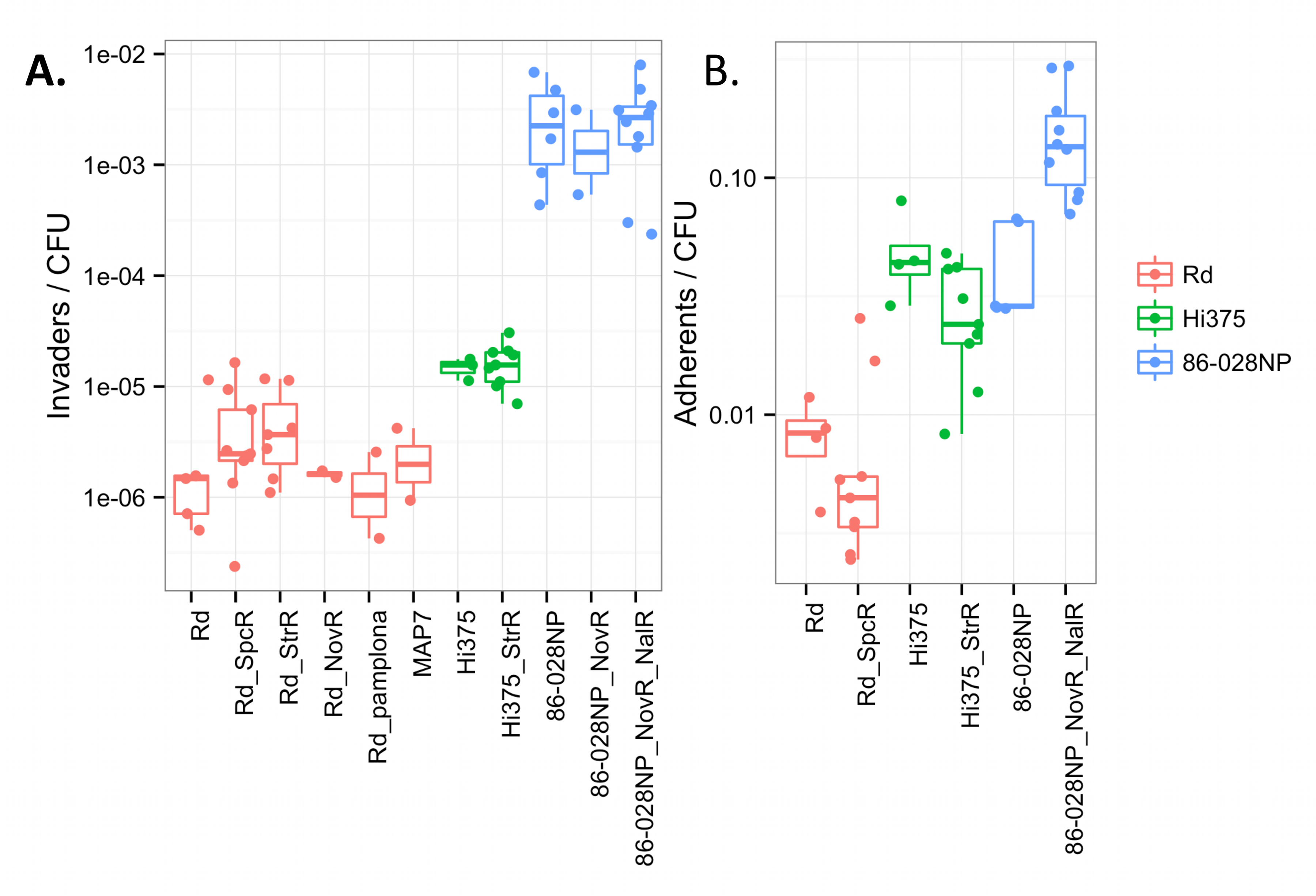

Supplement: S2 Fig — (A) Invasion of and (B) adhesion to A549 cells is shown for H. influenzae strains Rd KW20, Hi375, 86-028NP, and antibiotic resistant derivatives, including the parental strains. (TIF) [file ppat.1005576.s003.tif]

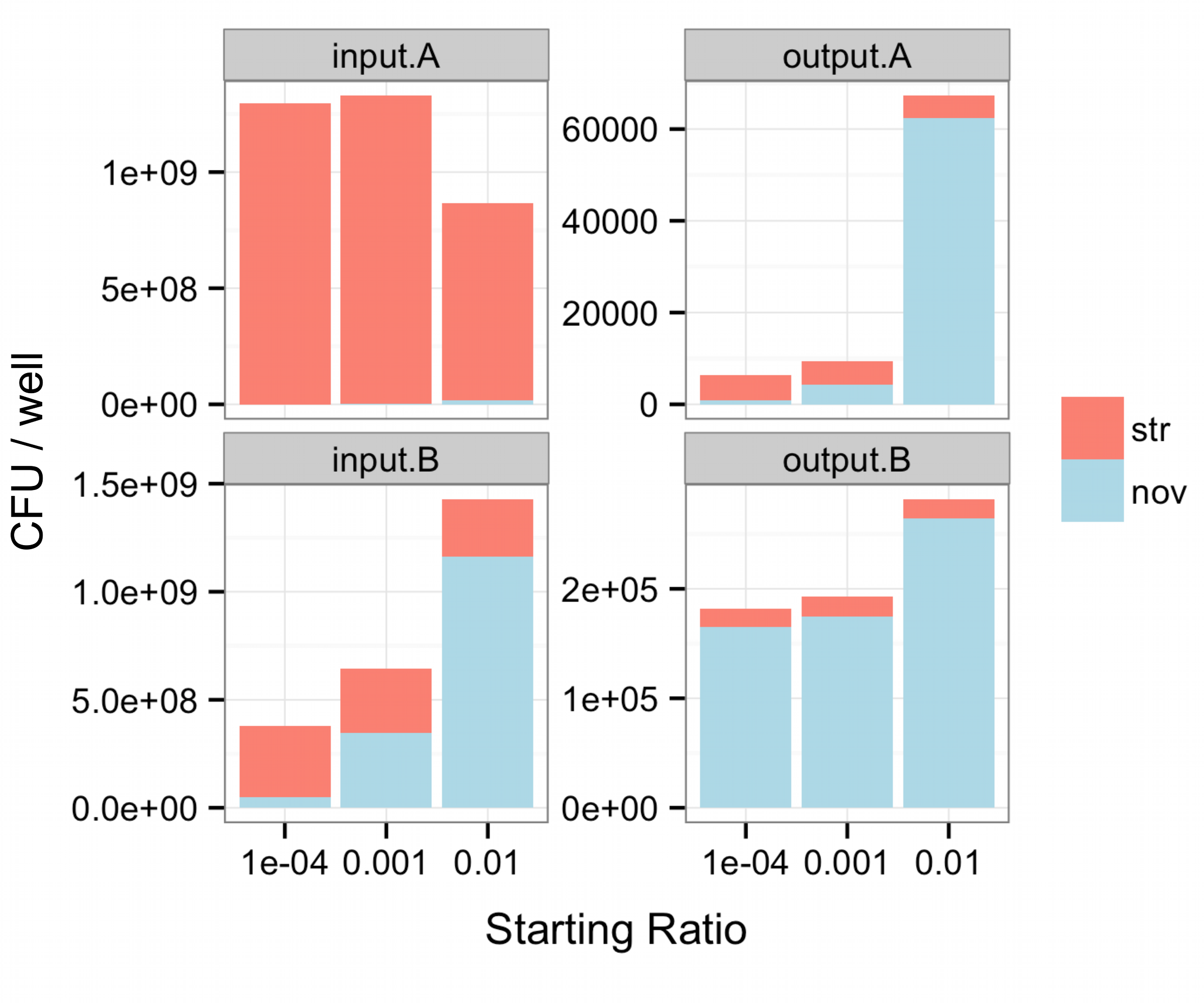

Supplement: S3 Fig — Two serial cycles of selection for intracellular invaders were conducted using three mixtures of 86-028NP NovR and Rd StrR cells, at 1:100, 1:1,000, or 1:10,000 ratios. Prior to the first infection (input.A), the bacterial cell suspension was titrated for the total NovR and StrR CFU used per well, and this closely matched the expected frequencies. After the first round of selection (output.A), dramatically fewer CFU were recovered, but NovR were proportionally much more abundant. Total unselected CFUs were pooled and titrated (input.B), showing that the proportion of NovR remained relatively the same in between cycles of selection for invasion. Finally, the second cycle of selection resulted in a higher yield with an even higher proportion of NovR colonies, representing a strong enrichment of 86-028NP over Rd, even when at a low relative abundance in the starting mixture. (TIF) [file ppat.1005576.s004.tif]

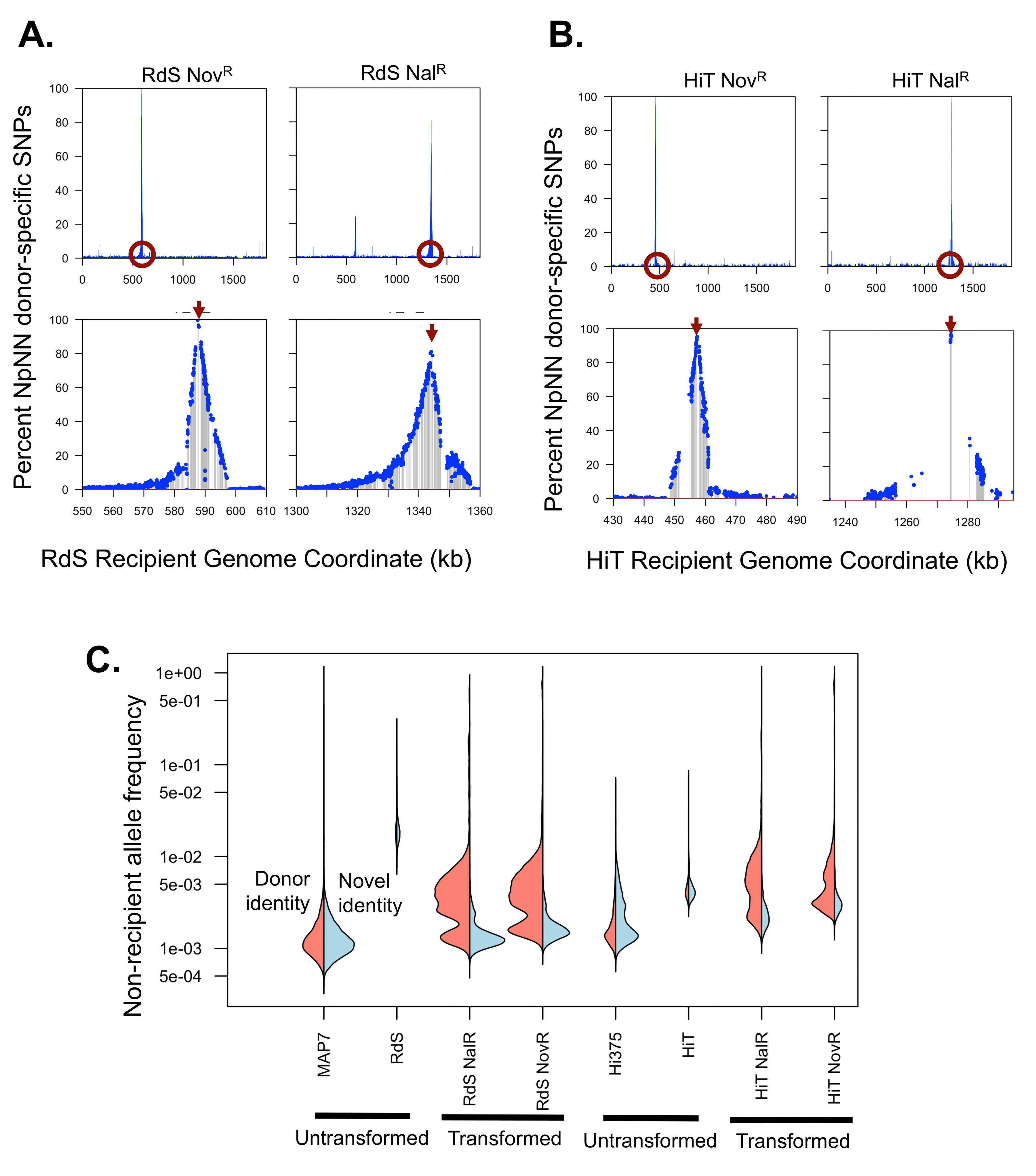

Supplement: S4 Fig — (A) and (B) NpNN-specific SNP frequencies as a function of chromosome coordinate for the RdS and HiT recipients, respectively, at Pool 0, prior to enrichment for invasive recombinants. Left panels: NovR-selected pools. Right panels: NalR-selected pools. Top panels: chromosome-wide view. Bottom panels: zoom on 60 kb windows around the antibiotic resistance markers. The peak SNP is the one conferring antibiotic resistance. (C) “Bean plots” summarizing 16 histograms of non-recipient allele frequencies for untransformed controls and the initial transformed recombinant pools. The left side (salmon-colored) of each bean shows a histogram for allele frequencies with donor allele identities, whereas the right side (light blue) shows a histogram for “novel” alleles (neither recipient nor donor). The latter are sequencing errors, while the former are sequencing errors for the control strains and a combination of sequencing errors and transformants for the transformed pools. (TIF) [file ppat.1005576.s005.tif]

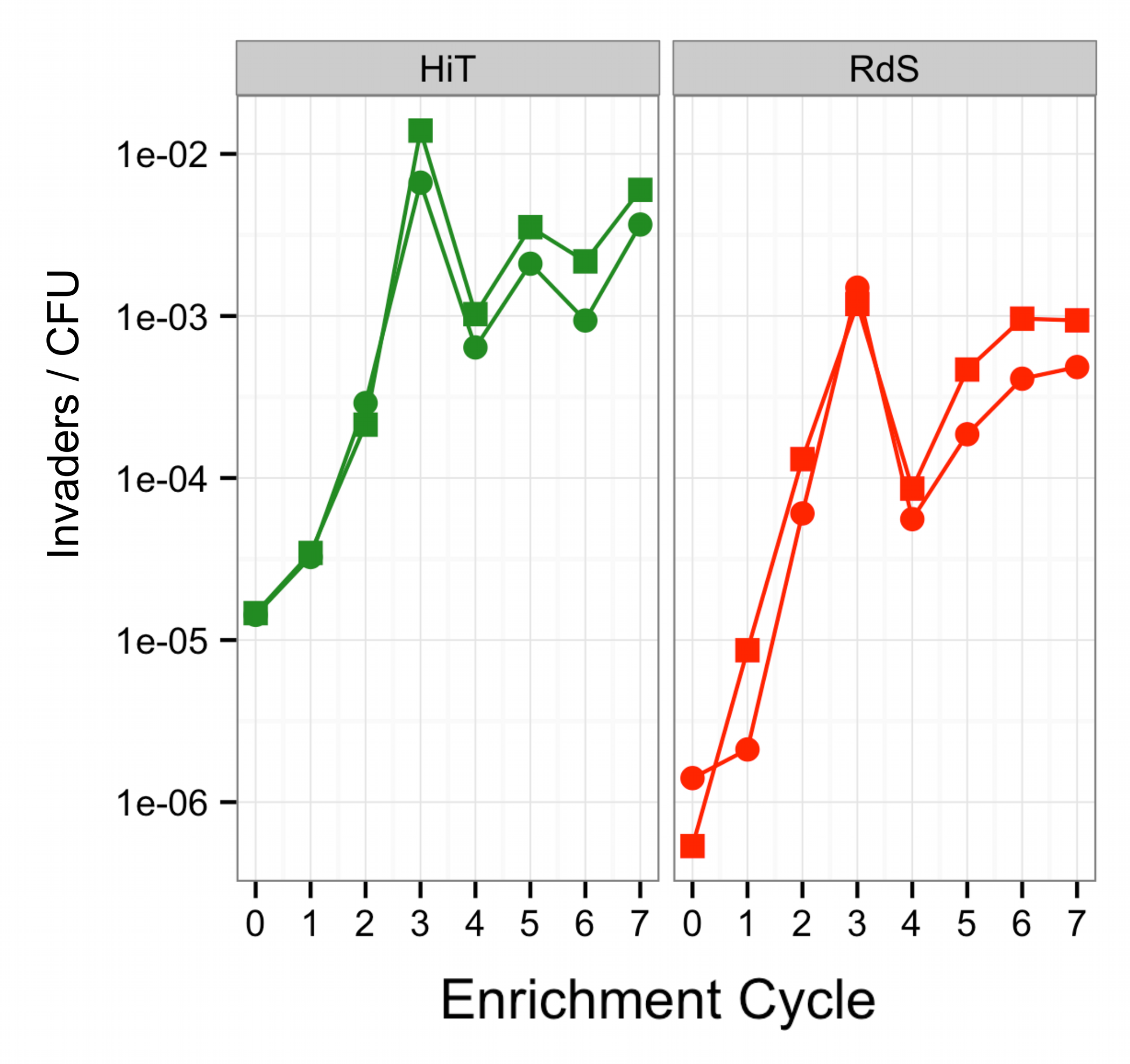

Supplement: S5 Fig — Invaders/CFU for pools during the initial eight serial selections for invasive recombinants. Recovered CFU that survived gentamicin treatment (Pool 1) served as input for the next cycle (which generated Pool 2). The values show the combined ability of clones in Pool n to invade airway epithelial cells, while the recovered colonies comprise Pool n+1. This procedure was carried out eight times. The apparent decline in invasiveness seen at Pool 4 appears to be an artifact, since no such decline was seen in the replicate assays (Fig 3A). Instead, this drop likely reflects that Pool 4 bacteria had been frozen and re-inoculated prior to the next cycle, combined with batch-to-batch variation of the confluent A549 cells used. (TIF) [file ppat.1005576.s006.tif]

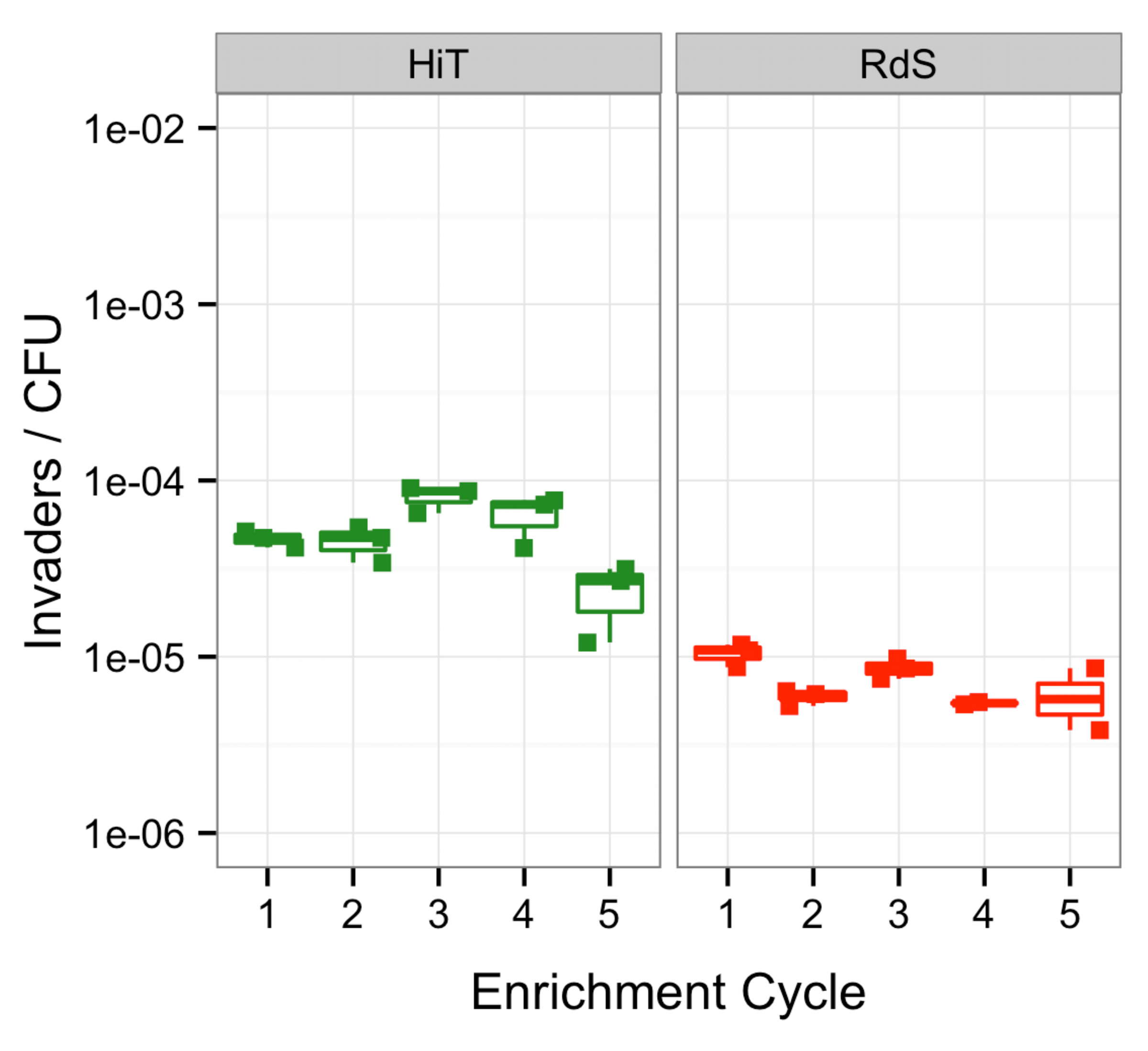

Supplement: S6 Fig — Control experiment using untransformed recipients cultures in triplicate found no increase in invasiveness over 5 serial selections. This experiment was conducted independently for each of the recipients and separately from the experimental enrichments to minimize enrichment of cross-contaminants. (TIF) [file ppat.1005576.s007.tif]

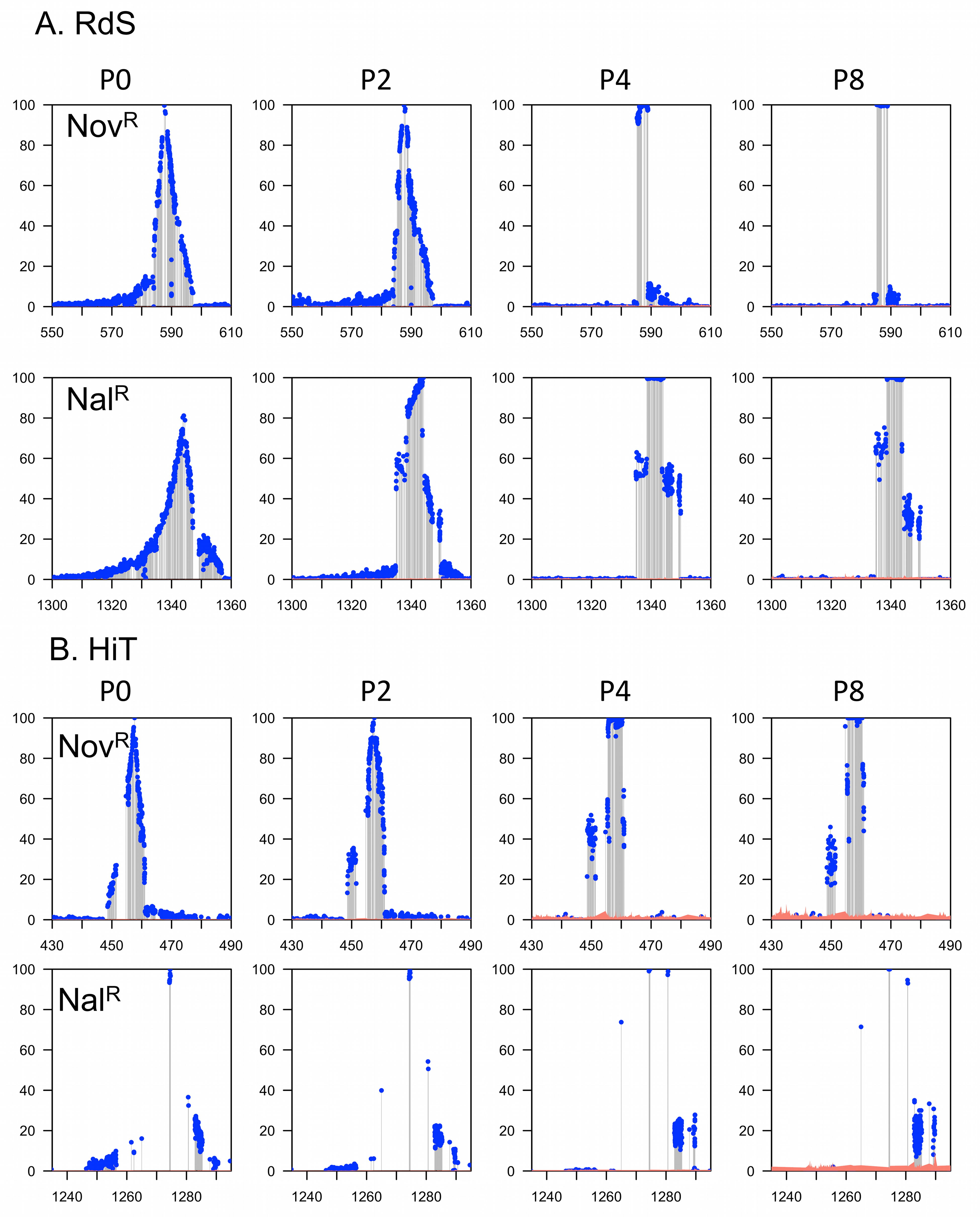

Supplement: S7 Fig — Genomic profiling at antibiotic-selected sites for both the (A) RdS and (B) HiT recipients at NovR (top) and NalR (bottom) sites (gyrB and gyrA respectively, see S6 Table) for Pools 0, 2, 4, and 8. Axes are as in other figures with x-axes indicating recipient genome coordinate (in kb) and the y-axis indicating donor allele frequency. RdS NovR contains a single clone at ~95% by Pool 8, while RdS NalR contains two dominant clones, one at ~70% and the other ~30%. HiT NovR contains two dominant clones (at ~30% and 70%), whereas HiT NalR appears to contain two clones at ~80% and ~20%. For this pool, only a single genotype (the one at ~80%) was recovered in the four individual clones collected from Pool 4. No other donor segments appeared at ~20%, so this is likely due to incomplete fixation of the invasive genotype after several rounds of selection. (TIF) [file ppat.1005576.s008.tif]

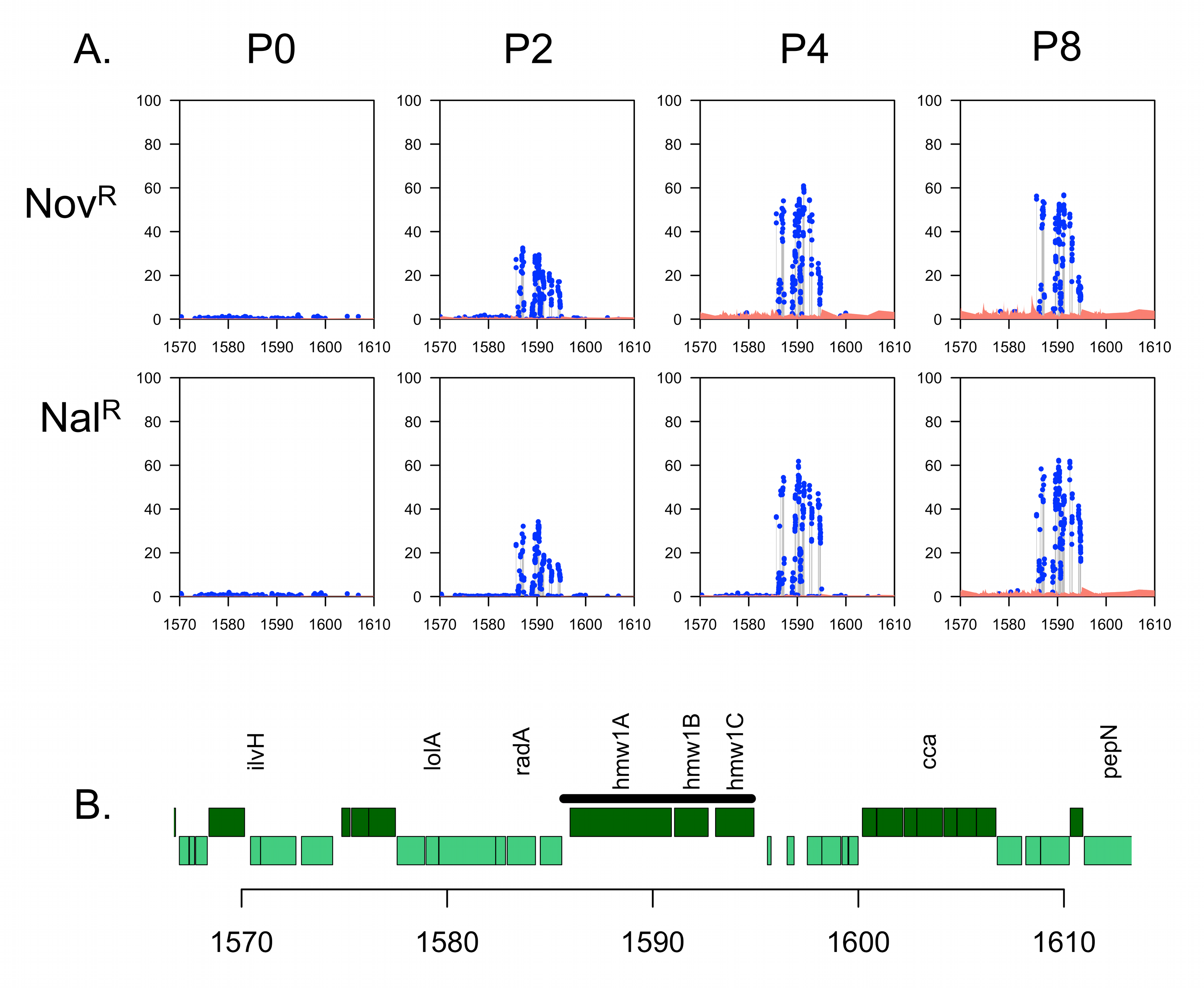

Supplement: S8 Fig — (A) Pools 0, 2, 4, and 8 for HiT NovR and HiT NalR as in other figures (x-axis is HiT recipient coordinate in kb, and y-axis is donor allele frequency). (B) Genomic map around the same interval. The thick black horizontal line shows the entire range of positions containing donor frequencies >5%. The affected interval spans only the hmw1 locus; no flanking variation was detected, unlike the situation at the yrbI-adjacent hmw2 Hi375, which was replaced by the hmw1 86-028NP allele. Donor allele frequencies are highly variable in this region. They are also highly consistent between the two pools, which was unexpected, as all other overlapping donor segments detected had distinct recombination breakpoints. Allele-specific PCR assays confirm this as read alignment artifact and confirm that the radA-proximal adhesin remain hmw1 Hi375 across strains (S9 Fig). (TIF) [file ppat.1005576.s009.tif]

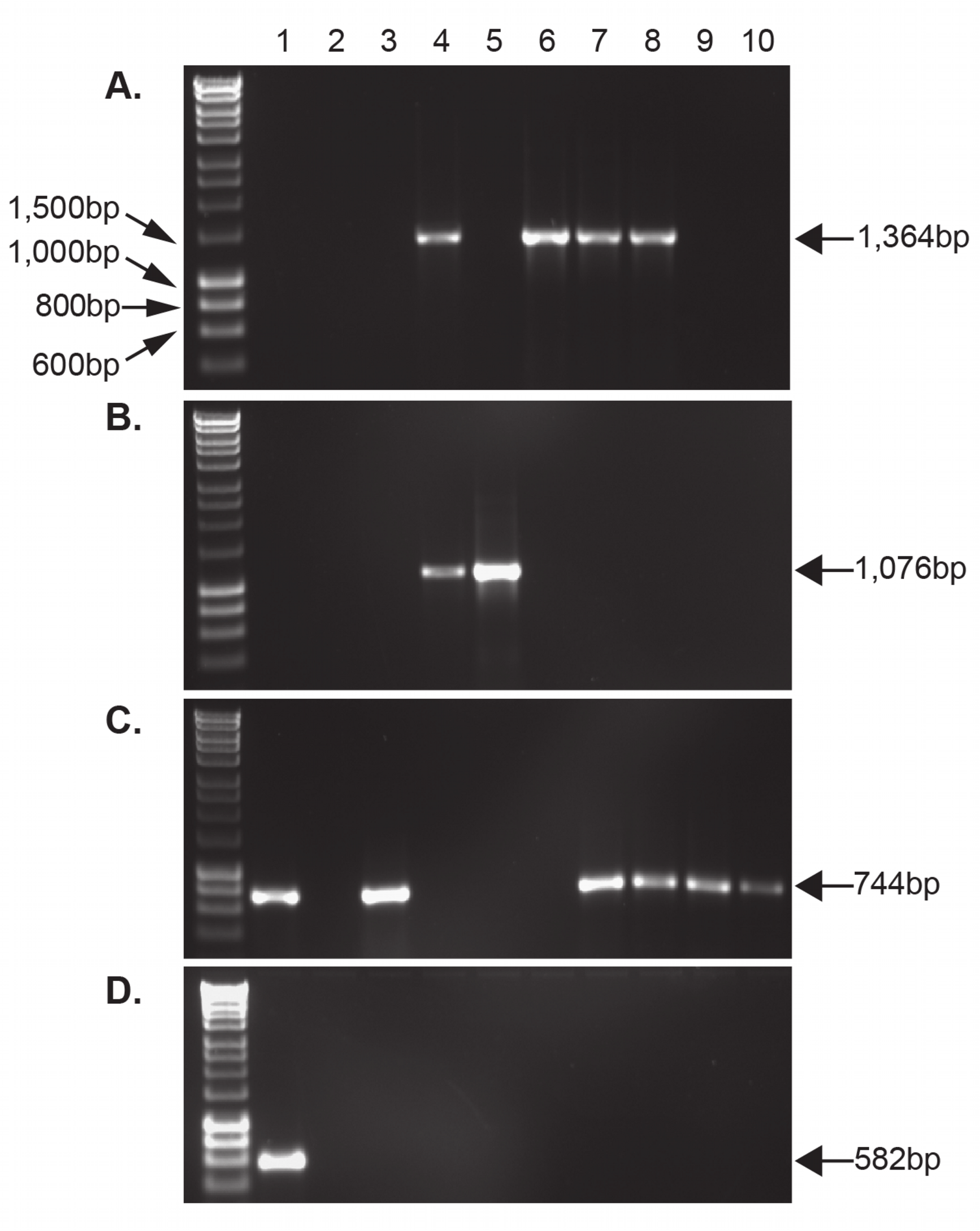

Supplement: S9 Fig — Strains are listed as it follows: (1) NpNN, (2) RdS, (3) rRdS, (4) HiT, (5) HiTΔhmw1A Hi375, (6) HiTΔhmw2A Hi375, (7) rHiT, (8) rHiTΔhmw1A 86-028NP, (9) rHiTΔhmw1A Hi375, (10) rHiTΔhmw1A 86-028NPΔhmw1A Hi375, and primers are in S10 Table. (A) Primers 1456+1458 identify hmw1A Hi375 (1,364 bp product); (B) primers 1456+1457 identify hmw2A Hi375 (1,076 bp product); (C) primers 1459+1460 identify hmw1A 86-028NP (744 bp product); and (D) primers 1461+1462 for hmw2A 86-028NP (582 bp product). Lanes 8 and 10 rendered a correct size band upon PCR with primers 1459+1460 because mutant strains lacking hmw1A 86-028NP were generated by partial deletion that maintains the annealing sites for the primers and product size. (TIF) [file ppat.1005576.s010.tif]

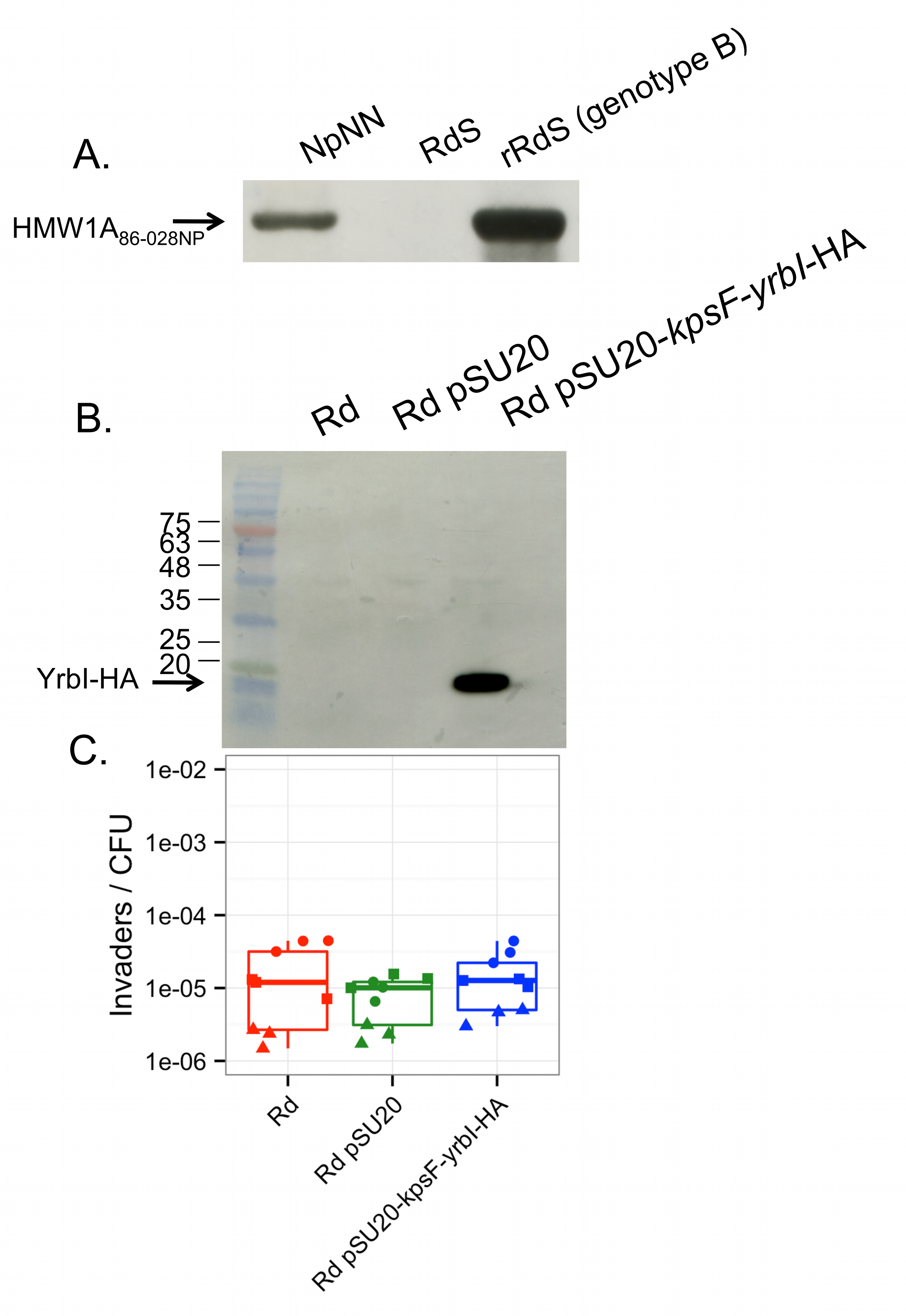

Supplement: S10 Fig — (A) Western blot showing expression of HMW1A86-028NP (154 KDa) adhesin. Whole cell extracts of NpNN, RdS and rRdS (P540, genotype B) were prepared and used to detect HMW by immunoblot with the guinea pig anti-HMW1A gp85 antibody. (B and C) Addition of the kpsF and yrbI alleles from 86-028NP on a plasmid does not increase intracellular invasion frequencies. (B) Western blot showing expression from plasmid carrying an interval carrying kpsF-yrbI from 86-028NP. Whole cell extracts of cultures (Rd, Rd pSU20, and Rd pSU20-kpsF-yrbI-HA) were prepared and used to detect Hap-HA by immunoblot with a rabbit anti-HA antibody, finding expression of the expected ~19.3-kDa protein in the expected strain. (C) The same strains were used to infect A549 cells and measure bacterial intracellular invasion. Experiments were performed three times in triplicate (different symbols denote independent experiments). (TIF) [file ppat.1005576.s011.tif]

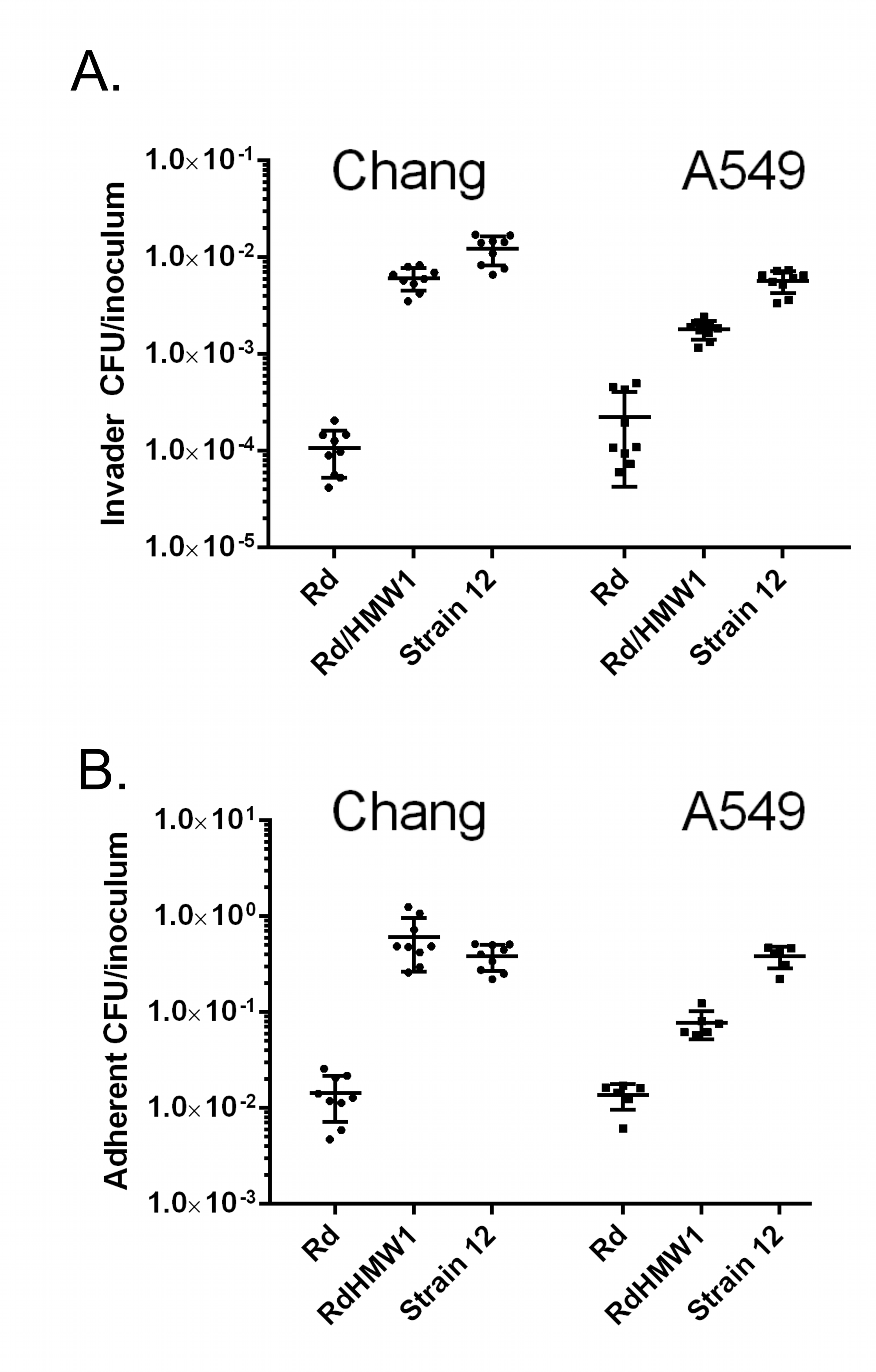

Supplement: S11 Fig — Invasion (A) and adhesion (B) by Rd, Rd hmw1 strain12, and Strain 12 bacterial strains into Chang and A549 epithelial cell lines. An alternative protocol that includes centrifugation to quickly bring bacteria into contact with the cell monolayer was used for these experiments, showing that both cell type and details of the infection procedure give qualitatively similar results. (TIF) [file ppat.1005576.s012.tif]
