# Supplementary material for: Transformed Recombinant Enrichment Profiling Rapidly Identifies HMW1 as an Intracellular Invasion Locus in Haemophilus influenzae
Source: PLoS Pathog. 2016 Apr 28;12(4):e1005576. doi: 10.1371/journal.ppat.1005576 (PMC4849778; doi:10.1371/journal.ppat.1005576)
Supplement: S1 Table — (DOCX) [file ppat.1005576.s013.docx]

**Table S1.** Strains and plasmid used*

| **Strain or plasmid** | **Description** | **Reference or source**** |
| --- | --- | --- |
| ***H. influenzae*** |  |  |
| P189 | Rd KW20, rough (unencapsulated) derivative of type d, source: R.J. Redfield (*aka* RR722) | [[4](#_ENREF_4)] |
| P192 (MAP7) | Rd Nov^R^ Nal^R^ Str^R^ Spc^R^ Kan^R^ Rif^R^ (*aka* RR666) | [[5](#_ENREF_5)] |
| P532 (RdS) | Rd Spc^R^, made by transforming P189 with P192 DNA, selecting for Spc^R^, and screening against other MAP7 resistance alleles | This study |
| P193 | Rd Str^R^, made as above but selecting for Str^R^ (*aka* RR514) | This study |
| P194 | Rd Nov^R,^ made as above but selecting for Nov^R^ (*aka* RR3148) | This study |
| P1171 | NTHi375, otitis media clinical isolate | [[6](#_ENREF_6)] |
| P531 (HiT) | NTHi375 Str^R^, made by transformation of the *rpsL* amplified from strain P193 | This study |
| P190 | 86-028NP, otitis media clinical isolate | [[1](#_ENREF_1)] |
| P195 | 86-028NP Nov^R^ (*aka* RR3129) | [[2](#_ENREF_2)] |
| P351 (NpNN) | 86-028NP Nal^R^ Nov^R^ (*aka* RR3131) | [[2](#_ENREF_2)] |
| P809 | Strain 12, otitis media clinical isolate, source: J. St. Geme (*aka* R2846) | [[7](#_ENREF_7)] |
| P812 | Rd KW20, source: J. St. Geme | [[4](#_ENREF_4)] |
| P813 | Rd/HMW1, made by addition of *hmw1ABC_strain12_* to Rd, source: J. St. Geme | [[8](#_ENREF_8)] |
| P540 (rRdS) | Rd Spc^R^, recombinant clone Nal^R^ s2, genotype B (rRdS) | This study |
| P551 (rHiT) | NTHi375 Str^R^, recombinant clone Nov^R^ s1, genotype E (rHiT) | This study |
| P834 | HiT rec-genotype EΔ*hmw1A*_86-028NP_ (rHiT Δ*hmw1A*_86-028NP_) | This study |
| P836 | HiTΔ*hmw1A_Hi375_* | This study |
| P837 | HiTΔ*hmw2A_Hi375_* | This study |
| P838 | HiT rec-genotype EΔ*hmw1A*_Hi375_ (rHiTΔ*hmw1A*_Hi375_) | This study |
| P839 | HiT rec-genotype EΔ*hmw1A*_86-028NP_Δ*hmw1A_Hi375_* (rHiTΔ*hmw1A*_86-028NP_Δ*hmw1A_Hi375_*) | This study |
| ***E. coli*** |  |  |
| Top10 | Cloning strain. F- *mcrA* Δ(*mrr-hsd*RMS-*mcr*BC) Φ80*lac*ZΔM15 Δ*lac*X74 *rec*A1 *ara*D139 Δ(*araleu*)7697 *gal*U *gal*K *rps*L (Str^R^) *end*A1 *nup*G | Thermofisher Scientific |
| SW102/DY380 | *E. coli* strain expressing lambda recombinase | [[9](#_ENREF_9)] |
| **Plasmid** |  |  |
| pGEMT-easy | Cloning vector | Promega |
| pJET2.1 | Cloning vector | Life Technologies |
| pJET2.1-(A-Erm^R^-B)*_hmw1AHi375_* | pJET2.1 with a 3,578 bp insert containing a *hmw1A_Hi375_* disruption cassette | This study |
| pJET2.1-(A-Erm^R^-B)*_hmw2AHi375_* | pJET2.1 with a 3,853 bp insert containing a *hmw2A_Hi375_* disruption cassette | This study |
| pGEMT-(A-Spc^R^-B)*_hmw1A86028NP_* | pGEMT-easy with a 4,253 bp insert containing a *hmw1A_86-028NP_* disruption cassette | This study |
| pSU20 | pACYC184 derivative, Cm^R^, 2,334 bp, shuttle vector for *H. influenzae* that allows for XGal screening | [[10](#_ENREF_10)] |
| pSU20-(*Pr::kpsF-yrbI*-HA) | pSU20 with a 2,241 bp insert containing *kpsF* and *yrbI* genes expressed under their own promoter | This study |

* Recombinant clones isolates from Pool 4 are listed in S3 Table

** Listed in S1 Text: Supplementary References
