# Supplementary material for: Transformed Recombinant Enrichment Profiling Rapidly Identifies HMW1 as an Intracellular Invasion Locus in Haemophilus influenzae
Source: PLoS Pathog. 2016 Apr 28;12(4):e1005576. doi: 10.1371/journal.ppat.1005576 (PMC4849778; doi:10.1371/journal.ppat.1005576)
Supplement: S2 Table — (DOCX) [file ppat.1005576.s014.docx]

**Table S2.** Pool and control sequencing statistics.

| **SAMN0** | **Sample** | **Ref** | **Pairs** | **Est. Cov.** | **%Merge** | **%Dup** | **%RecipMap** | **%DonorMap** |
| --- | --- | --- | --- | --- | --- | --- | --- | --- |
| 4392945 | Hi375 | Hi375 | 9,396,398 | 1879.3 | 55.80% | 5.50% | 99.90% | 96.70% |
| 4392946 | HiT | Hi375 | 3,188,760 | 637.8 | 61.30% | 7.10% | 99.90% | 99.10% |
| 4392947 | MAP7 | Rd | 11,986,693 | 2397.3 | 50.80% | 8.50% | 99.90% | 94.00% |
| 4392948 | RdS | Rd | 654,260 | 130.9 | 57.20% | 2.40% | 99.90% | 97.80% |
| 4392949* | NpNN | Hi375 | 1,376,875 | 275.4 | 34.60% | 1.90% | 95.70% | 99.80%* |
| 4392949* | NpNN | Rd | 1,376,875 | 275.4 | 34.50% | 2.00% | 92.50% | 99.80%* |
| 4392950 | HiT_NalR_P0 | Hi375 | 5,323,837 | 1064.8 | 35.30% | 5.40% | 99.90% | 98.80% |
| 4392951 | HiT_NalR_P1 | Hi375 | 1,351,021 | 270.2 | 34.60% | 2.80% | 98.60% | 98.10% |
| 4392952 | HiT_NalR_P2 | Hi375 | 6,505,976 | 1301.2 | 32.50% | 5.90% | 99.90% | 98.90% |
| 4392953 | HiT_NalR_P3 | Hi375 | 1,173,018 | 234.6 | 31.60% | 3.10% | 99.00% | 98.40% |
| 4392954 | HiT_NalR_P4 | Hi375 | 2,602,566 | 520.5 | 47.10% | 4.80% | 98.90% | 98.70% |
| 4392955 | HiT_NalR_P5 | Hi375 | 498,668 | 99.7 | 46.90% | 0.80% | 96.60% | 97.60% |
| 4392956 | HiT_NalR_P6 | Hi375 | 227,374 | 45.5 | 29.80% | 1.20% | 98.60% | 98.30% |
| 4392957 | HiT_NalR_P7 | Hi375 | 832,148 | 166.4 | 30.40% | 1.50% | 98.30% | 98.20% |
| 4392958 | HiT_NalR_P8 | Hi375 | 633,131 | 126.6 | 29.10% | 1.40% | 99.80% | 98.90% |
| 4392959 | HiT_NovR_P0 | Hi375 | 4,399,056 | 879.8 | 56.50% | 4.80% | 99.90% | 99.10% |
| 4392960 | HiT_NovR_P1 | Hi375 | 627,900 | 125.6 | 31.60% | 2.10% | 98.30% | 98.00% |
| 4392961 | HiT_NovR_P2 | Hi375 | 1,859,651 | 371.9 | 36.50% | 4.30% | 98.40% | 98.10% |
| 4392962 | HiT_NovR_P3 | Hi375 | 594,415 | 118.9 | 34.70% | 3.50% | 98.60% | 98.20% |
| 4392963 | HiT_NovR_P4 | Hi375 | 575,382 | 115.1 | 32.70% | 4.60% | 99.10% | 98.40% |
| 4392964 | HiT_NovR_P5 | Hi375 | 1,259,247 | 251.8 | 39.80% | 2.00% | 97.90% | 97.70% |
| 4392965 | HiT_NovR_P8 | Hi375 | 489,260 | 97.9 | 64.10% | 0.90% | 99.80% | 99.10% |
| 4392966 | RdS_NalR_P0 | Rd | 10,295,100 | 2059 | 51.80% | 6.80% | 99.90% | 97.80% |
| 4392967 | RdS_NalR_P2 | Rd | 3,002,546 | 600.5 | 65.10% | 2.20% | 99.50% | 98.20% |
| 4392968 | RdS_NalR_P3 | Rd | 1,168,294 | 233.7 | 28.90% | 3.30% | 99.50% | 97.60% |
| 4392969 | RdS_NalR_P4 | Rd | 2,293,706 | 458.7 | 30.20% | 2.90% | 98.60% | 97.30% |
| 4392970 | RdS_NalR_P5 | Rd | 1,494,058 | 298.8 | 41.10% | 1.40% | 97.60% | 96.90% |
| 4392971 | RdS_NalR_P6 | Rd | 118,518 | 23.7 | 33.00% | 0.60% | 98.30% | 97.10% |
| 4392972 | RdS_NalR_P7 | Rd | 974,181 | 194.8 | 38.00% | 1.60% | 98.50% | 97.30% |
| 4392973 | RdS_NalR_P8 | Rd | 1,252,651 | 250.5 | 61.40% | 1.00% | 99.40% | 98.00% |
| 4392974 | RdS_NovR_P0 | Rd | 8,521,941 | 1704.4 | 55.70% | 6.40% | 99.80% | 98.00% |
| 4392975 | RdS_NovR_P2 | Rd | 3,801,127 | 760.2 | 36.30% | 3.50% | 99.80% | 97.70% |
| 4392976 | RdS_NovR_P3 | Rd | 4,030,064 | 806 | 46.00% | 3.50% | 99.60% | 97.70% |
| 4392977 | RdS_NovR_P4 | Rd | 1,784,374 | 356.9 | 32.80% | 4.00% | 99.70% | 97.40% |
| 4392978 | RdS_NovR_P5 | Rd | 1,346,587 | 269.3 | 37.90% | 1.50% | 99.60% | 97.70% |
| 4392979 | RdS_NovR_P6 | Rd | 222,740 | 44.5 | 35.90% | 1.00% | 97.90% | 96.90% |
| 4392980 | RdS_NovR_P7 | Rd | 1,357,364 | 271.5 | 37.10% | 1.60% | 98.60% | 97.30% |
| 4392981 | RdS_NovR_P8 | Rd | 1,930,259 | 386.1 | 52.60% | 1.80% | 99.50% | 97.90% |

* Reads were submitted as BAM alignments to the 86-028NP reference genome.

Columns are as follows:

**SAMN0:** the BioSample accession number for the sample as SAMN0######.

**Sample:** Sample listed as strain name or recipient_selectedDonorMarker_PoolOrCloneID.

**Ref:** Reference genome for “self”-alignment.

**Pairs:** Total read pairs collected by Illumina sequencing (2x101nt).

**Est. Cov.:** Estimated genomic coverage based on total read pairs and genome size.

**% Merge:** The percent of reads that were merged by COPE.

**% Dup:** The percent of reads whose mappings were deemed duplicates by SamBlaster.

**%RMap:** Percent of reads that mapped to the appropriate recipient genome (Rd or Hi375).

**%DMap:** Percent of reads that mapped to the donor genome (86-028NP).
