# Supplementary material for: Transformed Recombinant Enrichment Profiling Rapidly Identifies HMW1 as an Intracellular Invasion Locus in Haemophilus influenzae
Source: PLoS Pathog. 2016 Apr 28;12(4):e1005576. doi: 10.1371/journal.ppat.1005576 (PMC4849778; doi:10.1371/journal.ppat.1005576)
Supplement: S3 Table — (DOCX) [file ppat.1005576.s015.docx]

**Table S3.** Recombinant clone sequencing statistics

| **SAMN0** | **Strain** | **Ref.** | **Pairs** | **Est. Cov.** | **%Merge** | **%Dup** | **%Nunmap** | **%RMap** | **%DMap** |
| --- | --- | --- | --- | --- | --- | --- | --- | --- | --- |
| 4392982 | HiT_NalR_s1 | Hi375 | 192,542 | 28.9 | 9.60% | 4.40% | 26.89% | 99.54% | 97.45% |
| 4392983 | HiT_NalR_s2 | Hi375 | 228,743 | 34.3 | 16.00% | 4.30% | 23.07% | 99.59% | 97.63% |
| 4392984 | HiT_NalR_s3 | Hi375 | 260,781 | 39.1 | 23.20% | 3.30% | 15.99% | 99.64% | 97.92% |
| 4392985 | HiT_NalR_s4 | Hi375 | 156,888 | 23.5 | 6.60% | 4.00% | 26.42% | 99.55% | 97.59% |
| 4392986 | Hit_NovR_s1 | Hi375 | 289,852 | 43.5 | 18.00% | 3.70% | 17.70% | 99.70% | 98.06% |
| 4392987 | Hit_NovR_s2 | Hi375 | 216,142 | 32.4 | 12.80% | 3.80% | 21.60% | 99.68% | 97.96% |
| 4392988 | Hit_NovR_s3 | Hi375 | 348,917 | 52.3 | 20.20% | 3.60% | 15.40% | 99.46% | 97.89% |
| 4392989 | Hit_NovR_s4 | Hi375 | 436,820 | 65.5 | 2.90% | 8.60% | 27.81% | 99.45% | 97.32% |
| 4392990 | RdS_NalR_s1 | Rd | 142,686 | 21.4 | 3.90% | 2.40% | 30.95% | 92.32% | 89.13% |
| 4392991 | RdS_NalR_s2 | Rd | 244,256 | 36.6 | 7.20% | 6.80% | 29.69% | 99.42% | 95.05% |
| 4392992 | RdS_NalR_s3 | Rd | 248,886 | 37.3 | 25.60% | 4.90% | 24.57% | 99.24% | 94.38% |
| 4392993 | RdS_NalR_s4 | Rd | 301,960 | 45.3 | 23.00% | 4.60% | 21.23% | 99.31% | 95.24% |
| 4392994 | RdS_NovR_s1 | Rd | 201,572 | 30.2 | 27.50% | 2.90% | 20.58% | 98.57% | 94.08% |
| 4392995 | RdS_NovR_s2 | Rd | 177,728 | 26.7 | 23.60% | 2.80% | 21.50% | 99.32% | 95.07% |
| 4392996 | RdS_NovR_s3 | Rd | 414,147 | 62.1 | 4.00% | 9.50% | 27.68% | 99.51% | 95.38% |
| 4392997 | RdS_NovR_s4 | Rd | 317,691 | 47.7 | 8.30% | 7.40% | 26.77% | 99.18% | 95.43% |

Columns as in S2 Table, except **%Nunmap** estimates uninformative reads as unmapped reads with strings of 5+ Ns, and **%RMap** and **%DMap** are adjusted to exclude these uninformative reads. A high proportion of clone reads from two independent sequencing experiments (independent DNA preps, library preps, and Illumina MiSeq runs) yielded a high proportion of reads that consisted of all or mostly all Ns. The reasons for this are unknown but were likely due to problems with a particular batch of Nextera XT library kits. The low coverage data was nonetheless able to corroborate the recombination breakpoints detected in the high quality high coverage pool datasets, as well as disambiguate donor segment assignment to individual clones. Aggregating datasets by clone genotype (as in S7 Table) further validated donor segment assignments in S8 Table.
