# Supplementary material for: Transformed Recombinant Enrichment Profiling Rapidly Identifies HMW1 as an Intracellular Invasion Locus in Haemophilus influenzae
Source: PLoS Pathog. 2016 Apr 28;12(4):e1005576. doi: 10.1371/journal.ppat.1005576 (PMC4849778; doi:10.1371/journal.ppat.1005576)
Supplement: S4 Table — (DOCX) [file ppat.1005576.s016.docx]

**Table S4:** Alignment statistics for the untransformed controls

| **Reads** | **Hi375** | **HiT** | **MAP7** | **RdS** | **NPNN** |
| --- | --- | --- | --- | --- | --- |
| **Reference** | **Hi375** | **Hi375** | **Rd KW20** | **Rd KW20** | **86-028NP** |
| Total positions | 1,850,897 | 1,850,897 | 1,830,138 | 1,830,138 | 1,914,490 |
| Ambiguous reference^1^ | - | - | 115 | 115 | - |
| Depth=0 | 22 | 55 | 116 | 182 | 1 |
| Depth<10 | 100 | 195 | 156 | 689 | 56 |
| Depth<50 | 233 | 545 | 261 | 783,986 | 20,256 |
| Filtered^2^ | 1,850,797 | 1,850,702 | 1,829,867 | 1,829,334 | 1,914,434 |
| % Invariant^3^ | 54.4% | 92.0% | 61.6% | 97.6% | 92.5% |
| Mean Depth | 652.2 | 227.5 | 822.9 | 49.0 | 107.6 |
| Mean Limit-of-detection (1/depth) | 1.53E-03 | 4.40E-03 | 1.22E-03 | 2.04E-02 | 9.30E-03 |
| Mean VarFreq | 1.12E-03 | 3.92E-04 | 7.89E-04 | 6.45E-04 | 7.21E-04 |
| Strand-biased positions^4^ | 4001 | 657 | 2811 | 175 | 643 |
| Unbiased Mean VarFreq | 1.12E-03 | 3.89E-04 | 7.84E-04 | 6.45E-04 | 7.14E-04 |
| Biased Mean VarFreq | 3.60E-03 | 9.36E-03 | 4.11E-03 | 2.28E-03 | 2.16E-02 |
| % Unbiased with VarFreq >0.01 | 0.29% | 0.17% | 0.11% | 2.37% | 2.02% |
| % Biased with VarFreq >0.01 | 6.20% | 17.66% | 6.79% | 3.43% | 29.86% |
| Detected variants (VarFreq >0.95)^5^ | 0 | 8 | 326 | 292 | 48 |

^1^ In the Rd KW20 genome, 115 positions have a non-ACGT base identity.

^2^ Positions with reference base ACGT that have at least 10 reads aligned; remaining statistics refer to this set of filtered bases.

^3^ % of positions for which no non-reference bases were detected (VarFreq=0)

^4^ Number of positions with evidence of strand-specific sequencing errors, as determined by Fisher’s exact test for strand bias between reference and alternate alleles with p-value <0.05.

^5^ Among positions with no strand bias, these positions represent single-nucleotide variants between strains and reference sequences. All variants can be accounted for as due to the introduction of the antibiotic resistance alleles or due to errors in the sequence reference (in the case of Rd KW20).
