# Supplementary material for: Transformed Recombinant Enrichment Profiling Rapidly Identifies HMW1 as an Intracellular Invasion Locus in Haemophilus influenzae
Source: PLoS Pathog. 2016 Apr 28;12(4):e1005576. doi: 10.1371/journal.ppat.1005576 (PMC4849778; doi:10.1371/journal.ppat.1005576)
Supplement: S5 Table — (DOCX) [file ppat.1005576.s017.docx]

**Table S5.** Transformation frequencies and estimated competence.

| **Recip** | **Rep** | **CFU/ml** | **Nov^R^/CFU** | **Nal^R^/CFU** | **Nov^R^ Nal^R^ /CFU** | **%Competence*** |
| --- | --- | --- | --- | --- | --- | --- |
| HiT | A | 3.1E+09 | 4.1E-03 | 4.1E-03 | 3.3E-06 | 19.1% |
| HiT | B | 3.1E+09 | 7.2E-03 | 5.7E-03 | 5.4E-06 | 13.1% |
| HiT | C | 3.8E+09 | 5.3E-03 | 3.6E-03 | 3.1E-06 | 15.8% |
| HiT | Mean | 3.3E+09 | 5.5E-03 | 4.5E-03 | 3.9E-06 | 16.0% |
| HiT | SD | 4.2E+08 | 1.5E-03 | 1.1E-03 | 1.3E-06 | 3.0% |
| RdS | A | 1.0E+09 | 2.3E-03 | 2.5E-03 | 8.6E-07 | 14.7% |
| RdS | B | 2.8E+09 | 1.1E-03 | 2.7E-03 | 4.4E-07 | 15.1% |
| RdS | Mean | 1.9E+09 | 1.7E-03 | 2.6E-03 | 6.5E-07 | 14.9% |
| RdS | SD | 1.3E+09 | 8.9E-04 | 1.2E-04 | 3.0E-07 | 0.3% |

* % Competence was calculated as expected doubles (Nov^R^Nal^R^/CFU) divided by the product of the singles (Nov^R^/CFU * Nal^R^/CFU).
