# Supplementary material for: Transformed Recombinant Enrichment Profiling Rapidly Identifies HMW1 as an Intracellular Invasion Locus in Haemophilus influenzae
Source: PLoS Pathog. 2016 Apr 28;12(4):e1005576. doi: 10.1371/journal.ppat.1005576 (PMC4849778; doi:10.1371/journal.ppat.1005576)
Supplement: S6 Table — (DOCX) [file ppat.1005576.s018.docx]

**Table S6.** Allele frequencies around antibiotic resistances in Pool 0*.

| **Recip** | **Select** | **Position** | **Recip** | **Donor** | **Depth** | **%recip** | **%donor** | **%other** |
| --- | --- | --- | --- | --- | --- | --- | --- | --- |
| HiT | Nal^R^ | 1,274,373 | A | C | 388 | 5.93% | 94.07% | 0.00% |
| HiT | Nal^R^ | 1,274,406 | C | T | 424 | 4.95% | 94.81% | 0.24% |
| HiT | Nal^R^ | 1,274,496 | T | G | 546 | 2.20% | 97.80% | 0.00% |
| HiT | Nal^R^ | 1,274,522 | C | A | 541 | 0.00% | 99.82% | 0.18% |
| HiT | Nal^R^ | 1,274,532 | T | G | 473 | 3.17% | 96.83% | 0.00% |
| HiT | Nal^R^ | 1,274,634 | C | T | 354 | 2.82% | 96.61% | 0.56% |
| HiT | Nal^R^ | 1,274,652 | T | A | 342 | 2.92% | 96.78% | 0.29% |
| HiT | Nov^R^ | 457,257 | A | G | 532 | 5.08% | 94.92% | 0.00% |
| HiT | Nov^R^ | 457,263 | A | T | 511 | 6.65% | 93.35% | 0.00% |
| HiT | Nov^R^ | 457,333 | G | T | 523 | 4.59% | 95.41% | 0.00% |
| HiT | Nov^R^ | 457,600 | C | A | 434 | 0.00% | 100.00% | 0.00% |
| HiT | Nov^R^ | 457,953 | T | C | 382 | 13.87% | 86.13% | 0.00% |
| HiT | Nov^R^ | 457,956 | A | C | 367 | 13.08% | 86.92% | 0.00% |
| HiT | Nov^R^ | 457,962 | G | A | 402 | 12.69% | 87.31% | 0.00% |
| RdS | Nal^R^ | 1,343,717 | C | G | 893 | 28.00% | 71.89% | 0.11% |
| RdS | Nal^R^ | 1,343,720 | T | C | 891 | 26.82% | 72.73% | 0.45% |
| RdS | Nal^R^ | 1,343,759 | T | G | 952 | 19.75% | 80.25% | 0.00% |
| RdS | Nal^R^ | 1,344,100 | C | A | 993 | 18.83% | 81.17%** | 0.00% |
| RdS | Nal^R^ | 1,344,472 | C | T | 865 | 38.61% | 61.39% | 0.00% |
| RdS | Nal^R^ | 1,344,488 | A | G | 703 | 32.43% | 67.57% | 0.00% |
| RdS | Nal^R^ | 1,344,490 | T | C | 690 | 31.74% | 66.96% | 1.30% |
| RdS | Nov^R^ | 586,842 | A | G | 635 | 17.17% | 82.83% | 0.00% |
| RdS | Nov^R^ | 586,851 | C | A | 622 | 16.24% | 83.76% | 0.00% |
| RdS | Nov^R^ | 586,854 | T | A | 620 | 16.13% | 83.87% | 0.00% |
| RdS | Nov^R^ | 587,579 | G | T | 972 | 0.31% | 99.69% | 0.00% |
| RdS | Nov^R^ | 587,846 | C | A | 1134 | 3.09% | 96.74% | 0.18% |
| RdS | Nov^R^ | 587,969 | C | A | 983 | 4.27% | 95.73% | 0.00% |
| RdS | Nov^R^ | 588,549 | C | T | 813 | 16.24% | 83.64% | 0.12% |

* Highlighted rows indicate positions of antibiotic resistance causing donor alleles.

** The RdS Nal^R^ recombinant shows only ~80% Nal^R^ allele due to a mistake during pooling. An additional ~20% of the pool was Nov^R^.
