# Supplementary material for: Transformed Recombinant Enrichment Profiling Rapidly Identifies HMW1 as an Intracellular Invasion Locus in Haemophilus influenzae
Source: PLoS Pathog. 2016 Apr 28;12(4):e1005576. doi: 10.1371/journal.ppat.1005576 (PMC4849778; doi:10.1371/journal.ppat.1005576)
Supplement: S7 Table — (DOCX) [file ppat.1005576.s019.docx]

**Table S7.** Non-reference alleles at reliable SNP positions in transformed pools and untransformed controls*

| **DNA** | **Type** | **Total SNPs** | **Donor Freq>0** | **Novel Freq>0** | **Depth**  **(Median ± MAD)** | | | **Un- mapped to Recip** | **Re-mapped to donor** | **% Re-mapped** |
| --- | --- | --- | --- | --- | --- | --- | --- | --- | --- | --- |
| MAP7 | Control | 36,869 | 9,839 | 10,273 | 876 | ± | 268 | 20,955 | 656 | 3.13% |
| RdS | Control | 36,869 | 406 | 731 | 52 | ± | 13 | 935 | 22 | 2.35% |
| RdS Nal^R^ | Pool 0 | 36,869 | 30,998 | 6,911 | 787 | ± | 142 | 19,861 | 3,054 | 15.38% |
| RdS Nov^R^ | Pool 0 | 36,869 | 29,838 | 5,405 | 636 | ± | 125 | 18,890 | 2,969 | 15.72% |
| Hi375 | Control | 19,520 | 4,027 | 7,762 | 701 | ± | 169 | 13,992 | 43 | 0.31% |
| HiT | Control | 19,520 | 786 | 1,202 | 236 | ± | 43 | 3,891 | 8 | 0.21% |
| HiT Nal^R^ | Pool 0 | 19,520 | 14,882 | 2,119 | 448 | ± | 117 | 9,125 | 169 | 1.85% |
| HiT Nov^R^ | Pool 0 | 19,520 | 10,840 | 1,534 | 325 | ± | 70 | 8,477 | 577 | 6.81% |

* The distribution of non-zero allele frequencies is depicted in S4 Figure part C beanplots.
