# Supplementary material for: Transformed Recombinant Enrichment Profiling Rapidly Identifies HMW1 as an Intracellular Invasion Locus in Haemophilus influenzae
Source: PLoS Pathog. 2016 Apr 28;12(4):e1005576. doi: 10.1371/journal.ppat.1005576 (PMC4849778; doi:10.1371/journal.ppat.1005576)
Supplement: S8 Table — (DOCX) [file ppat.1005576.s020.docx]

**Table S8.** Clone genotype assignments.

| **Background** | **Resistance** | **Clone** | **Genotype** |
| --- | --- | --- | --- |
| RdS | Nal | s1 | A |
| RdS | Nal | s2 | B (P540) |
| RdS | Nal | s3 | A |
| RdS | Nal | s4 | A |
| RdS | Nov | s1 | C |
| RdS | Nov | s2 | C |
| RdS | Nov | s3 | C |
| RdS | Nov | s4 | C |
| HiT | Nal | s1 | D |
| HiT | Nal | s2 | D |
| HiT | Nal | s3 | D |
| HiT | Nal | s4 | D |
| HiT | Nov | s1 | E (P551) |
| HiT | Nov | s2 | F |
| HiT | Nov | s3 | E |
| HiT | Nov | s4 | E |
