# Supplementary material for: Transformed Recombinant Enrichment Profiling Rapidly Identifies HMW1 as an Intracellular Invasion Locus in Haemophilus influenzae
Source: PLoS Pathog. 2016 Apr 28;12(4):e1005576. doi: 10.1371/journal.ppat.1005576 (PMC4849778; doi:10.1371/journal.ppat.1005576)
Supplement: S9 Table — (DOCX) [file ppat.1005576.s021.docx]

**Table S9.** Donor segments detected in each isolated genotype.

| **Reference** | **Genotype** | **Type** | **Begin** | **End** |
| --- | --- | --- | --- | --- |
| Rd_KW20 | A | hitchhiker | 1,056,042 | 1,065,007 |
| Rd_KW20 | A | Nal^R^ | 1,338,588 | 1,349,925 |
| Rd_KW20 | A | invasion | 1,734,959 | 1,766,413 |
| Rd_KW20 | B | hitchhiker | 862,451 | 877,939 |
| Rd_KW20 | B | hitchhiker | 887,142 | 893,453 |
| Rd_KW20 | B | Nal^R^ | 1,334,937 | 1,344,101 |
| Rd_KW20 | B | invasion | 1,732,805 | 1,750,336 |
| Rd_KW20 | C | Nov^R^ | 585,269 | 588,958 |
| Rd_KW20 | C | hitchhiker | 1,494,874 | 1,495,778 |
| Rd_KW20 | C | invasion | 1,744,519 | 1,750,957 |
| Rd_KW20 | C | hitchhiker | 1,760,431 | 1,760,794 |
| **Rd_KW20** | **ABC** | **min. invasion** | **1,744,519** | **1,750,336** |
| Hi375 | D | hitchhiker | 1,139,838 | 1,143,114 |
| Hi375 | D | invasion | 1,172,910 | 1,187,650 |
| Hi375 | D | Nal^R^ | 1,274,284 | 1,274,653 |
| Hi375 | E | hitchhiker | 374,543 | 375,508 |
| Hi375 | E | Nov^R^ | 454,718 | 460,941 |
| Hi375 | E | invasion | 1,178,771 | 1,183,728 |
| Hi375 | E | hitchhiker | 1,478,173 | 1,482,010 |
| Hi375 | F | Nov^R^ | 448,886 | 460,427 |
| Hi375 | F | invasion | 1,172,475 | 1,185,610 |
| **Hi375** | **DEF** | **min. invasion** | **1,178,771** | **1,183,728** |
| Hi375 | D-EF | D-exclusive | 1,139,838 | 1,142,951 |
| Hi375 | D-EF | D-exclusive | 1,185,743 | 1,187,650 |
| Hi375 | D-EF | D-exclusive (Nov^R^) | 1,274,284 | 1,274,653 |

Genotypes correspond to S8 Table. Coordinates are inclusive and with reference to the recipient. Only SNP positions that were deemed reliable (see Supplementary Results) were used to define recombination breakpoints. The spurious interval that contains the *radA-*adjacent *hmw1_Hi375_* interval is not reported. Donor segment type can be: “**Nov^R^**/**Nal^R^**” for the segment containing the selected donor antibiotic resistance, “**invasion**” for the segment containing the putative invasion allele/locus, or “**hitchhiker**” for segments that are neither of these. “**Min. invasion**” defines the intersection of putative invasion tracts within the same recipient background. Though “hitchhiking” segments are unlikely to be due to the selections, subtle effects on intracellular invasion by these intervals are not ruled out.

In particular, donor variation exclusive to genotype D (“**D-exclusive**”) may carry alleles/loci involved in intracellular invasion (Figure 7). Genes affected by recombination in these intervals (aside from the *gyrA* allele conferring Nal^R^) are NF38_05675 (mannose-6-phosphate isomerase pseudogene), NF38_05680 (hypothetical), NF38_05685 (*qseB* two-component system transcription factor), NF38_05690 (*qseC* two-component system sensor), NF38_09010 (RNase P), NF38_05855 (anaerobic NO production, iron-sulfur cluster repair), and NF38_05860 (*moaA*).
