# Supplementary material for: Transformed Recombinant Enrichment Profiling Rapidly Identifies HMW1 as an Intracellular Invasion Locus in Haemophilus influenzae
Source: PLoS Pathog. 2016 Apr 28;12(4):e1005576. doi: 10.1371/journal.ppat.1005576 (PMC4849778; doi:10.1371/journal.ppat.1005576)
Supplement: S10 Table — (DOCX) [file ppat.1005576.s022.docx]

**Table S10.** Primers used in this study.

| Purpose | Dir | Primer name | Primer number | Sequence (5´-3´) |
| --- | --- | --- | --- | --- |
| RdS | F | *rpsL*-RdKW20-F | 1497 | GATTACGTGTAGATCGCCTTAAAACAGGTA |
| RdS | R | *rpsL*-RdKW20-R | 1798 | CCAAAATAATTTCATTTGCTAATACACGCT |
| Interval | F | hmw1A-7-D | 13CAN | CACTCGCTAGTGGTTGTTAATGATGAA |
| Interval | R | hmw1A-recomb-strat2 | 1273 | TATCCCCCAACTTTAATCGCGTCT |
| Deletion | F | Hmw1A-casset-F | 1199 | GCAAATGATAAAGTAATTTAATTGTTCAACTAACCTTAGGAGAAAATATGATTCCGGGGATCCGTCGACC |
| Deletion | R | Hmw1A-casset-R-strat2 | 1274 | TCTCTCCCCCTTCTTTACCTGAAAGGTCGATAACTGCGCCTGTTTTTATGTAGGCTGGAGCTGCTTCG |
| FlankA | F | radA-hmw1A-375-FragA-Fw | 1463 | ATTGTTTTGAGCCCAGCAAATAACCTC |
| FlankA | R | radA-hmw1A-375-FragA-SmaI-Rv | 1464 | TCCCCCGGGTTACACCTAAAGATAGTAACATAG |
| FlankB | F | radA-hmw1A-375-FragB-SmaI-Fw | 1465 | TCCCCCGGGTATCACAAATTTCACTTTTAATGTAG |
| FlankB | R | radA-hmw1A-375-FragB-Rv | 1466 | GATGTTGCAGTTAGGGTCGCGGCTCC |
| FlankA | F | yrbI-hmw2A-375-FragA-Fw | 1467 | AGGCATAAAAATGCTGATGGATGCGGGTATT |
| FlankA | R | yrbI-hmw2A-375-FragA-SmaI-Rv | 1468 | TCCCCCGGGCCATGCCCAAGGATAGCAATATA |
| FlankB | F | yrbI-hmw2A-375-FragB-SmaI-Fw | 1469 | TCCCCCGGGGTAAAACTTGGCAATGTTACCAAT |
| FlankB | R | yrbI-hmw2A-375-FragB-Rv | 1470 | CTACGGCTGTCCATCGTCAGCAACATTGGTAC |
| Cloning | F | NTHI1981upstr-F1 | 1219 | caattgcacgccacctgcagatgcttg |
| Cloning | R | NTHI1982-R1-HA | 1218 | TTAAGCGTAGTCTGGGACGTCGTATGGGTATTGCCCCATATTTTTCACTGATTTTAG |
| Allele ID | F | HMW1A-2A-NTHi375-Fw | 1456 | GGAATGGATGTAGTACACGGCACA |
| Allele ID | R | HMW1A-NTHi375-Rv | 1458 | CCGCTCCGACCCTCACTCCAAAGA |
| Allele ID | R | HMW2A-375-Rv | 1457 | GCATTGCTGTCAATGGATAAATAA |
| Allele ID | F | HMW1A-86028NP-Fw | 1459 | AATAACTACAAAACTCCAGGGGTG |
| Allele ID | R | HMW1A-86028NP-Rv | 1460 | GGTAATATTGACTTTATCAGAAGA |
| Allele ID | F | HMW2A-86028NP-Fw | 1461 | ATTACGCTTGGTACGGGTTTTTTA |
| Allele ID | R | HMW2A-86028NP-Rv | 1462 | ATCACTGCTACCGGTAGCTGTAAT |
